# Involvement of TRPC Channels in Lung Cancer Cell Differentiation and the Correlation Analysis in Human Non-Small Cell Lung Cancer

**DOI:** 10.1371/journal.pone.0067637

**Published:** 2013-06-28

**Authors:** Hong-Ni Jiang, Bo Zeng, Yi Zhang, Nikoleta Daskoulidou, Hong Fan, Jie-Ming Qu, Shang-Zhong Xu

**Affiliations:** 1 Department of Pulmonary Medicine, Zhongshan Hospital, School of Medicine, Fudan University, Shanghai, China; 2 Centre for Cardiovascular and Metabolic Research, Hull York Medical School, University of Hull, Hull, United Kingdom; 3 Department of Thoracic Surgery, Zhongshan Hospital, School of Medicine, Fudan University, Shanghai, China; 4 Department of Pulmonary Medicine, Huadong Hospital, School of Medicine, Fudan University, Shanghai, China; University of Hawaii Cancer Center, United States of America

## Abstract

The canonical transient receptor potential (TRPC) channels are Ca^2+^-permeable cationic channels controlling the Ca^2+^ influx evoked by G protein-coupled receptor activation and/or by Ca^2+^ store depletion. Here we investigate the involvement of TRPCs in the cell differentiation of lung cancer. The expression of TRPCs and the correlation to cancer differentiation grade in non-small cell lung cancer (NSCLC) were analyzed by real-time PCR and immunostaining using tissue microarrays from 28 patient lung cancer samples. The association of TRPCs with cell differentiation was also investigated in the lung cancer cell line A549 by PCR and Western blotting. The channel activity was monitored by Ca^2+^ imaging and patch recording after treatment with all-*trans*-retinoic acid (ATRA). The expression of TRPC1, 3, 4 and 6 was correlated to the differentiation grade of NSCLC in patients, but there was no correlation to age, sex, smoking history and lung cancer cell type. ATRA upregulated TRPC3, TRPC4 and TRPC6 expression and enhanced Ca^2+^ influx in A549 cells, however, ATRA showed no direct effect on TRPC channels. Inhibition of TRPC channels by pore-blocking antibodies decreased the cell mitosis, which was counteracted by chronic treatment with ATRA. Blockade of TRPC channels inhibited A549 cell proliferation, while overexpression of TRPCs increased the proliferation. We conclude that TRPC expression correlates to lung cancer differentiation. TRPCs mediate the pharmacological effect of ATRA and play important roles in regulating lung cancer cell differentiation and proliferation, which gives a new understanding of lung cancer biology and potential anti-cancer therapy.

## Introduction

Although the survival rate has been improved since the introduction of third-generation anti-neoplastic agents and epidermal growth factor receptor (EGFR) tyrosine kinase inhibitors, lung cancer is still the leading cause of cancer deaths in the world [1], [2], [3], [4], [5]. Therefore, understanding the pathogenesis of lung cancer development and identification of new potential targets are important for therapeutic strategy development.

Ca^2+^ is important in the signalling cascades of tumorigenesis. The transient receptor potential (TRP) ion channel family has been implicated in the regulation of cancer growth and progression via the modulation of Ca^2+^ influx and the downstream signals including gene transcription [6], [7], [8]. Among the six subfamilies (TRPC, TRPM, TRPV, TRPP, TRPML and TRPA) of TRP channels, the canonical TRPs (TRPCs) have been suggested as protein tyrosine kinase or G protein-coupled receptor-operated Ca^2+^ channels (ROCs) or internal Ca^2+^ store-operated channels (SOCs), which mediate the Ca^2+^ entry evoked by many hormones and growth factors. Therefore, the inhibition of these channel activity or expression leads to functional changes in cancer cell proliferation, migration, colony formation and tumour growth [6], [9]. Recently, several studies have demonstrated the existence of TRPC in different types of cancer cells or cancer tissues, such as TRPC1, 3, 6 in breast cancer MCF7 cells [10], [11] and liver cancer HepG2 cells [12], TRPC1, 3, 4 in prostate cancer LNCaP cells [13], [14], TRPC1, 4, 6, 7 in renal cell carcinoma [15], TRPC1, 3, 5, 6 in human malignant gliomas [16], TRPC1, 3–7 in neuroblastoma IMR-32 cells [17], TRPC3 in human astrocytoma 1321N1 cells [18], TRPC6 in esophageal and gastric cancer [19], TRPC1, 3, 4, 6 and their spliced variants in ovarian cancer [9], [20], and TRPC1, 4 in basal cell carcinoma [21]. Moreover, evidences from *in vitro* experiments have shown that overexpression of TRPC channels or silence of gene expression with siRNA can regulate cell proliferation or cell survival, suggesting these genes are important in cancer biology [6], [9], [20].

The expression of TRPC1, 3, 4, 6 in lung cancer has been detected [22], [23] and the association of TRPC3 expression with the prognosis of lung adenocarcinoma has been described [23]. However, the correlation of TRPC expression with the differentiation grade of lung cancer and the underlying mechanism are largely unknown. Here we aimed to identify the expression of TRPCs in human lung cancer and determine the roles of TRPCs in the regulation of cancer cell differentiation and proliferation using specific TRPC channel blocking antibodies. We also examined the potential correlation of TRPC expression with cancer differentiation grade, cell type and smoking by real-time PCR and immunohistochemistry on the lung cancer tissue microarrays. To further examine the relationship of TRPC expression with cell differentiation, ATRA, a potent cell differentiation inducer for many cell types, was used in an *in vitro* lung cancer cell model.

## Materials and Methods

### Patients and Lung Tissue Samples

Twenty-eight patients (17 males and 11 females) aged at 61.1±1.7 years with non-small cell lung cancer (NSCLC) were recruited between November 2008 and December 2009. All patients with NSCLC were diagnosed as clinically staged I or II lung cancer and received operation in the Thoracic Surgery of Zhongshan Hospital. The eligible patients had previously untreated, histologically or cytologically proved NSCLC. The patients received either preoperative chemotherapy or radiotherapy were excluded from this study. The lung cancer tissue and the normal lung tissue surrounding the tumour beyond 2 cm in distance were obtained from same patient. The snap-frozen tissues were used for mRNA analysis and the formalin-fixed tissues for immuocytochemistry study. The project was approved by the Ethics Committee of Zhongshan Hospital of Fudan University, and the patients gave written consent in accordance with the Declaration of Helsinki.

### Lung Cancer Tissue Microarrays

Lung cancer tissue microarrays were made using formalin-fixed cancer tissues [24]. Tissue cores with 2 mm in diameter were collected based on visual alignment with the corresponding hematoxylin and eosin (HE) staining. One core of normal lung tissue and two cores of tumour tissue were taken from each patient and placed into recipient paraffin blocks. The tissue sections with 5-µm thickness were used for immunostaining. All samples on the tissue microarrays were examined by a pathologist with histologically classification and differentiation grade according to the WHO classification [25].

### Cells Culture and Gene Transfection

A549 cell line, a commonly used lung cancer cell model derived from adenocarcinomic human alveolar basal epithelial cells, was grown in DMEM/F12 medium (Invitrogen, Paisley, UK) containing 10% foetal bovine serum (FBS), 100 units/ml penicillin and 100 µg/ml streptomycin, and maintained at 37°C under 95% air and 5% CO_2._ Human TRPC1, TRPC3 and TRPC6 were amplified from the cDNA of human ovarian cancer cells and human TRPC4 were amplified from the cDNA of human aortic endothelial cells with 100% identity to the sequences in the Genbank (accession numbers: X89066 (TRPC1); U47050 (TRPC3), NM_016179 (TRPC4α) and BC093660 (TRPC6). The TRPC cDNAs were subcloned into pcDNA3.1 or pEGFP-C1 vectors and their functional expression has been confirmed as we reported [9]. A549 cells were transfected with TRPC1, 3, 4, and 6 plasmid cDNAs in pcDNA3 vector using Lipofectamine2000 (Invitrogen). The transfected cells were plated in 48-well plate for experiment.

### Giemsa Staining, Mitosis and Cell Proliferation Assays

A549 cells were plated into 3.5-cm dishes with a final density of 4×10^4^ cells per dish. The cells were treated with 1 µM ATRA or vehicle. The culture medium was replenished every 24 h. The cells were fixed with methanol for 10 min and stained with a 1∶9 diluted working Giemsa solution (Sigma) in PBS at pH 6.5 for 45 min, and washed by water and air-dried. The total cell number and mitotic cells were counted on the pictures photographed under 200× magnification. Cell proliferation was also determined using WST-1 assay (Roche, UK) [9].

### RT-PCR and Real-time PCR

Total RNA was extracted from human lung and lung cancer tissues or A549 cells using Trizol reagent (Invitrogen, UK). The RNA was quantified with a nanophotometer (Implen, German). The mRNA (1 µg) was reverse-transcribed to cDNA using Multiscribe Reverse transcriptase (Applied Biosystems, USA) and random primers (Promega, UK). Quantitative RT-PCR was performed using StepOne™ Real-Time PCR System or ABI PRISM 7900 HT Sequence Detection System (Applied Biosystems, UK). The primer set was designed across intron and the sequences were given in the Table S1. Each reaction contained 1×SYBR Universal Master Mix (Applied Biosystems) 10 µl, cDNA 1 µl, and forward and reverse primers 1.5 µl each. The human housekeeping gene glyceraldehyde-3-phosphate dehydrogenase GAPDH was used as an internal standard. Non-template or non-RT was set as negative control. The PCR cycle consisted of an initial cycle of 50°C for 2 min followed by 95°C for 10 minutes, then 50 repeated cycles of 95°C for 15 s, 54°C annealing temperature for 30 s, and the primer extension at 72°C for 30 s.

### Antibodies, Western Blotting and Immunohistochemistry

Rabbit polyclonal anti-TRPC antibodies (T1E3, T5E3, T367E3 and T45E3) were generated against the extracellular third loop (E3) region near the channel pore [26]. The specificity of E3-targeting antibodies was tested by ELISA, Western blotting and functional assays, and fluorescence activated cell sorting (FACS) [9], [26]. The procedure for Western blotting has been described previously [26]. Briefly, cells were lysed in RIPA buffer (Sigma-Aldrich, Poole, UK) and proteins were separated on 10% SDS-PAGE gel before transferring onto nitrocellulose membrane. The blot was incubated with rabbit anti-TRPC antibodies (1∶200) overnight at 4°C, washed with phosphate buffered saline (PBS), and incubated with goat anti-rabbit IgG-HRP at 1∶2000 dilution (Sigma). The rabbit anti-β-actin (Santa Cruz Biotech, USA) at 1∶400 dilution was used as an internal standard for protein quantification. Visualization was carried out using ECLplus detection reagents (GE Healthcare, UK) and exposure to X-ray films. The quantification was analysed using Image J software (NIH, USA). The immunostaining procedure was similar to our reports [9], [27] and the VECTASTAIN ABC system (Vector Laboratories, Peterborough, UK) was used. The rabbit anti-TRPC1, 3, 4 and 6 antibodies purchased from Abcam (Cambridge, UK) were used for human lung tissue and lung cancer section staining. The staining quantification was assessed by scoring positive stained cells and staining intensity ranked as 0 (negative), 1 (weak), 2 (intermediate) and 3 (strong) [28].

### Whole-cell Patch Clamp

The whole cell currents were recorded using Axoclamp 2B or Axopatch B200 patch clamp amplifier and controlled with pClamp 10 software. The procedures for recording TRPC currents were similar to our previous reports [29], [30]. A 1-s ramp voltage protocol from –100 mV to +100 mV was applied at a frequency of 0.2 Hz from a holding potential of 0 mV. Signals were sampled at 3 kHz and filtered at 1 kHz. The glass microelectrode with a resistance of 3–5 MΩ was used. The 200 nM Ca^2+^ pipette solution (115 CsCl, 10 EGTA, 2 MgCl_2_, 10 HEPES, and 5.7 CaCl_2_ in mM, pH was adjusted to 7.2 with CsOH and the osmolarity was adjusted to ∼290 mOsm with mannitol). The calculated free Ca^2+^ was 200 nM using EQCAL (Biosoft, Cambridge, UK). The standard bath solution contained (mM): 130 NaCl, 5 KCl, 8 D-glucose, 10 HEPES, 1.2 MgCl_2_ and 1.5 CaCl_2_. The pH was adjusted to 7.4 with NaOH. The experiment was performed at room temperature (23–25°C).

### Ca^2+^ Imaging

A549 cells were loaded with 2 µM Fura-PE3 AM at 37°C for 30 min in Ca^2+^-free bath solution, followed by a 20-min wash in standard bath solution at room temperature. Fura-PE3 fluorescence was monitored with an inverted epifluorescence microscope (Nikon Ti-E, Japan). A xenon arc lamp provided excitation light, the wavelength of which was selected by a Nikon imaging system controlled by NIS Elements 3.0 software. Dual wavelength excited at 340 nm and 380 nm was used for Fura-PE3 fluorescence, and emission was collected via a 510-nm filter and photographed by Orca-R2 CCD camera (Hamamatsu, Japan). The ratio of Ca^2+^ dye fluorescence at F_340_/F_380_ wavelength was measured [30]. All the experiments were performed at room temperature.

### Reagents and Chemicals

All general salts were purchased from Sigma-Aldrich (Poole, UK). Gadolinium chloride (Gd^3+^), 2-aminoethoxydiphenyl borate (2-APB), trypsin, all-*trans* retinoic acid (ATRA), and PCR primers were purchased from Sigma-Aldrich, and Fura-PE3 AM was from Invitrogen (Paisley, UK).

### Statistics

Data are expressed as mean ± s.e.m. The statistical significance was analysed using ANOVA and the difference among the groups was assessed with Dunnett's *t*-test in the SPSS software. Student’s *t* test was applied for two group comparison. The Ridit analysis was used for the semiquantitative data of immunostaining experiment. The *P* value <0.05 was considered significance.

## Results

### Expression of TRPCs in Lung Cancer

**Figure 1:**
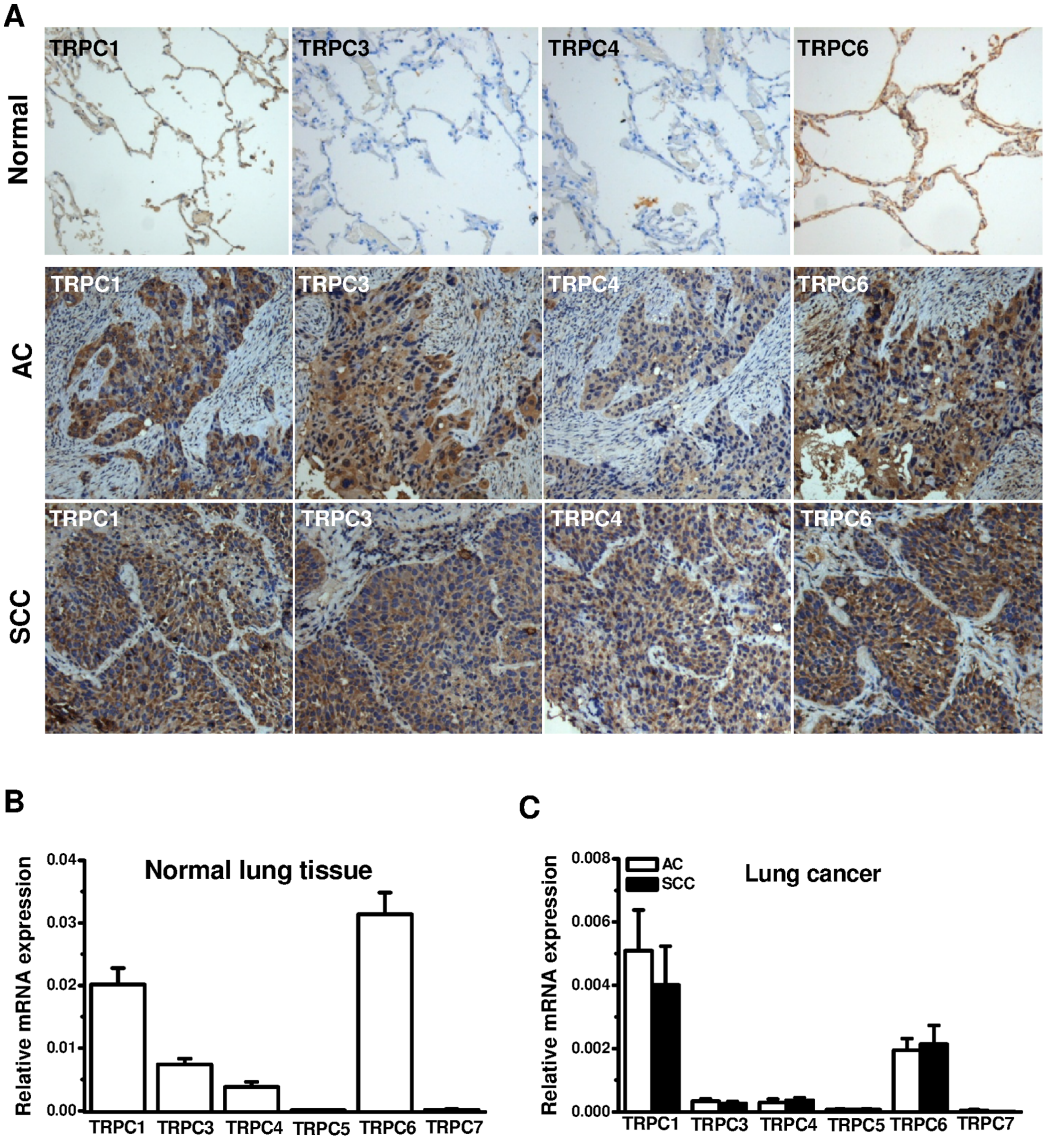
Distribution of TRPC isoforms in human lung and lung cancer. **A,** Examples of human normal lung (*n* = 20) and lung cancer tissue sections (*n* = 28) including adenocarcinoma (AC) and squamous cell carcinoma (SCC) were stained with anti-TRPC1, anti-TRPC3, anti-TRPC4 and anti-TRPC6 antibodies using VECTASTAIN ABC system. The positive staining was shown as brown colour. The nuclei were counter-stained by hematoxylin. **B,** The mRNA was detected by real-time PCR in normal lung tissues using the primers in Table S1. The GAPDH was used as internal house-keeping gene control for quantification (*n* = 25 patients for TRPC1, 4, 5 and 6 groups; *n* = 24 for TRPC3; and *n = *9 for TRPC7). **C,** The mRNA levels in lung cancer tissues (AC: *n* = 9–15; SCC: *n* = 8–11).

The expression of TRPCs in normal human lung and lung cancer tissues was examined by immunostaining (Fig. 1A). In normal lung tissue sections, the alveolar epithelial cells were stained with anti-TRPC1 and anti-TRPC6 antibodies, but the staining for TRPC3 and TRPC4 were negative or very weak. In lung squamous cell carcinoma sections, the squamous cells were strongly stained with anti-TRPC1, anti-TRPC3, anti-TRPC4 and anti-TRPC6 antibodies. Similarly, the positive staining for TRPC1, TRPC3, TRPC4 and TRPC6 was also seen in lung adenocardionoma sections. Using real-time PCR, we quantified the expression of TRPCs in normal lung and cancer tissues. The mRNAs of TRPC1, 3, 4 and 6 were detected in both normal lung and lung cancer tissues. The expression level of TRPC1 and TRPC6 was much higher than that of TRPC3 and TRPC4. The mRNAs for TRPC5 and TRPC7 were undetectable in normal and lung cancer tissues (Fig. 1B–C). These data suggest the existence of TRPC1, 3, 4, 6 isoforms in NSCLC.

### Correlation of TRPC Expression to Cancer Differentiation Grade

**Figure 2:**
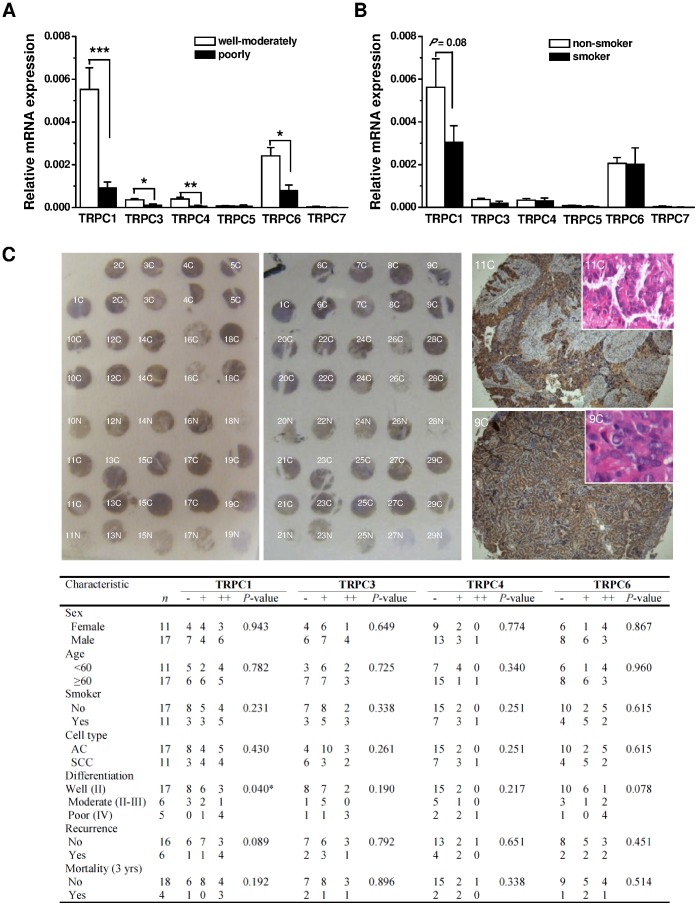
Correlation of TRPC expression to differentiation grade, smoking, cell type, sex and age determined by real-time PCR and immunostaining. **A,** The mRNA expression of TRPCs in lung cancer tissues with well-moderate (grade II (*n* = 17) and grade III (*n* = 6)) or poor (grade IV, *n* = 5) differentiation grade was detected by real-time PCR. GAPDH was used as housekeeping gene control. **B,** The mRNA expression of TRPCs in the lung cancer tissues obtained from smoker (20 cigarettes per day for more than 10 years, *n* = 11) and non-smoker (*n* = 17). **C**, Example of two tissue microarrays with normal lung (N) and lung cancer (C) labels were stained with anti-TRPC1 antibody. The cell type and differentiation grade of each section were characterized by HE-staining. The two examples (11C and 9C) with well-differentiated adenocarcinoma were shown in the insets. The correlation of staining intensity to other factors was shown in the table and the significance was assessed by Ridit analysis. * *P*<0.05, ** *P*<0.01, *** *P*<0.001.

The difference in mRNA levels between cancer differentiation grades, cell types, smoker and non-smoker was detected by real-time PCR. The expression of TRPC1, 3, 4 and 6 channels was significantly lower in the poorly differentiated lung cancer than in the well-moderately differentiated group (Fig. 2A, also see Table S2). However, there was no significant difference between the smoker and non-smoker groups (Fig. 2B). Linear multivariance regression analysis also showed a negative correlation of the mRNA expression of TRPC1, TRPC3, TRPC4 and TRPC6 to lung cancer differentiation grade with standardized β coefficient sequence of TRPC1 (−0.563)>TRPC4 (−0.360) >TRPC6 (+0.271) and TRPC3 (−0.057), respectively. Stepwise regression showed that TRPC1 was a significant variable (P<0.01), but the correlation of cancer differentiation grade to sex, age, smoking, and cancer cell type was not significant.

We also investigated the correlation of lung cancer differentiation grade to the TRPC protein expression levels using semiquantitative immunostaining. Two tissue microarrays with 20 normal lung tissues and 28 NSCLC samples including 15 cases with adenocarcinoma, 11 cases with squamous cell carcinoma, and 2 cases with mixed cell types of adenosquamous carcinoma were constructed (Fig. 2C). The lung cancer cell type was confirmed by HE-staining. The TRPC1, TRPC3, and TRPC6 in the adjacent microarray tissue sections were strongly stained with anti-TRPC1, 3, and 6 antibodies with a percentage of positively stained cancer sections of 71.4%, 75.0% and 71.4%, respectively, while mild staining was seen for TRPC4 with a positive staining percentage of 32.1%. For normal lung sections on the microarrays, the positive staining percentage for TRPC1, 3, 4 and 6 were 70%, 70%, 45%, and 85%, respectively. The Ridit analysis showed that the staining intensity of TRPC1 was associated with the differentiation grade (Fig. 2C). However, linear multivariance regression analysis showed no significant correlation of the protein staining scores of TRPC to the lung cancer differentiation grade, cell type, smoking, age and sex.

### Upregulation of TRPC Expression by Chronic Treatment with ATRA

**Figure 3:**
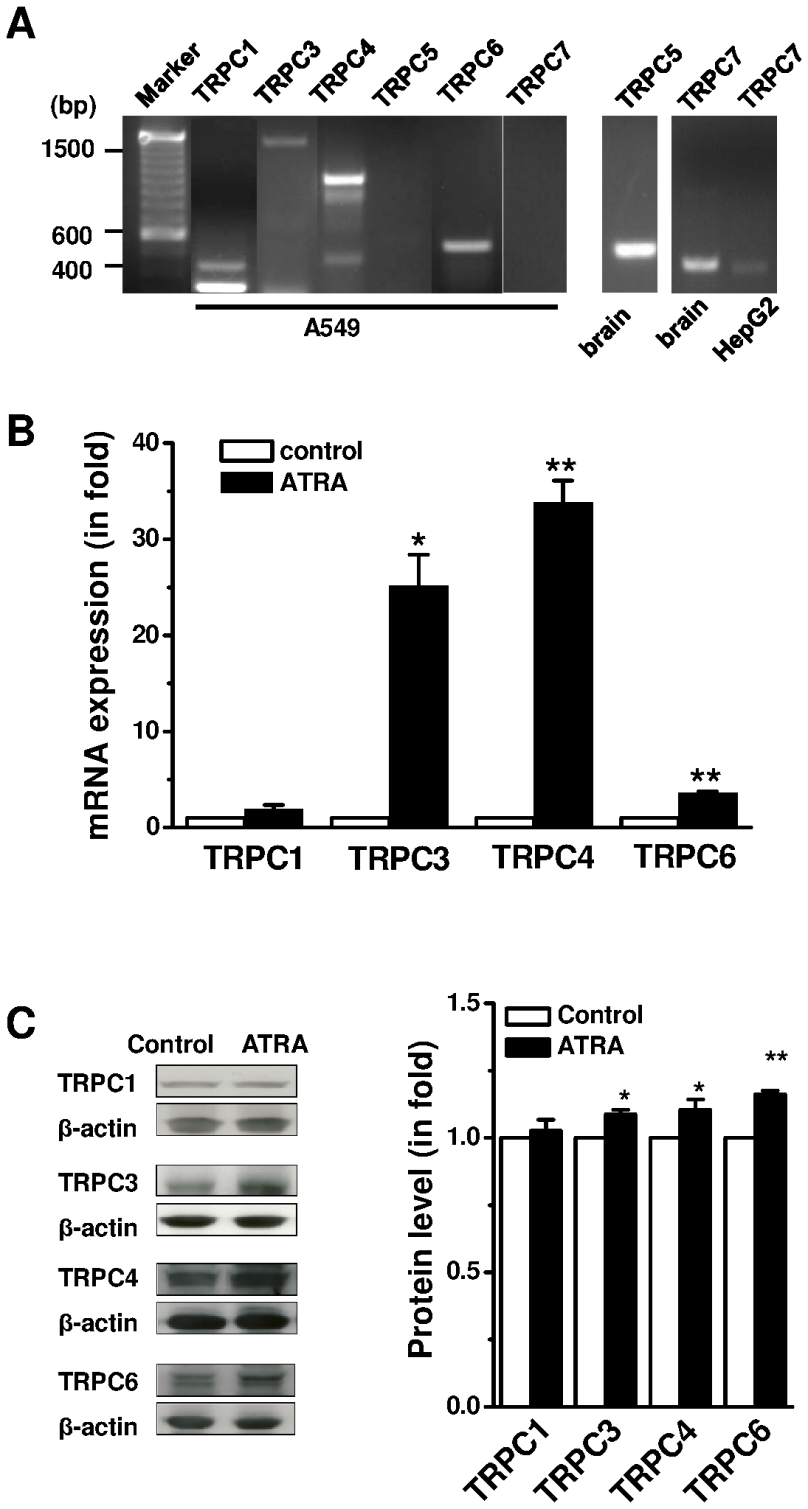
Upregulation of TRPCs by ATRA in A549 cells. **A,** The mRNAs of TRPC1, 3, 4 and 6 were detected in A549 cells by RT-PCR using the primer set in Table S1. The PCR bands for TRPC5 and TRPC7 were negative in A549 cells, but positive in human brain or HepG2. **B,** The mRNA was detected by real-time PCR in the A549 cells treated with all-*trans* retinoic acid (ATRA, 1 µM) for 96 hours. The β-actin was used as a housekeeping gene for relative quantification. The 2^(−ΔΔCt)^ method was used for calculation. **C,** The TRPC proteins were quantified by Western blotting using anti-TRPC1 (T1E3), anti-TRPC3, anti-TRPC4 (T45E3) and anti-TRPC6 antibodies. Anti-β-actin antibody was used for relative protein quantification (*n* = 3 independent experiments and each experiment with triplicate samples).

TRPC1, 3, 4 and 6 were detected in A549 cells, but TRPC5 and TRPC7 were undetectable although the primer sets for TRPC5 and TRPC7 can amplify the mRNA isolated from brain or HepG2 cells (Fig. 3A). The two TRPC1 bands in the gel were α and β isoforms, and the bands for TRPC4 were α, β, γ and δ isoforms, respectively, as we described in ovarian cancer cells [9]. The mRNA and protein levels for TRPC3, TRPC4 and TRPC6 were significantly increased by chronic treatment with 1 µM ATRA for 96 hours (Fig. 3B–C), however, the regulation on TRPC1 expression was not significant. These data further suggest that the expression of some TRPC isoforms is associated with cell differentiation.

### Effects of ATRA on Ca^2+^ Release and Influx in A549 Cells and TRPC Channel Activity

**Figure 4:**
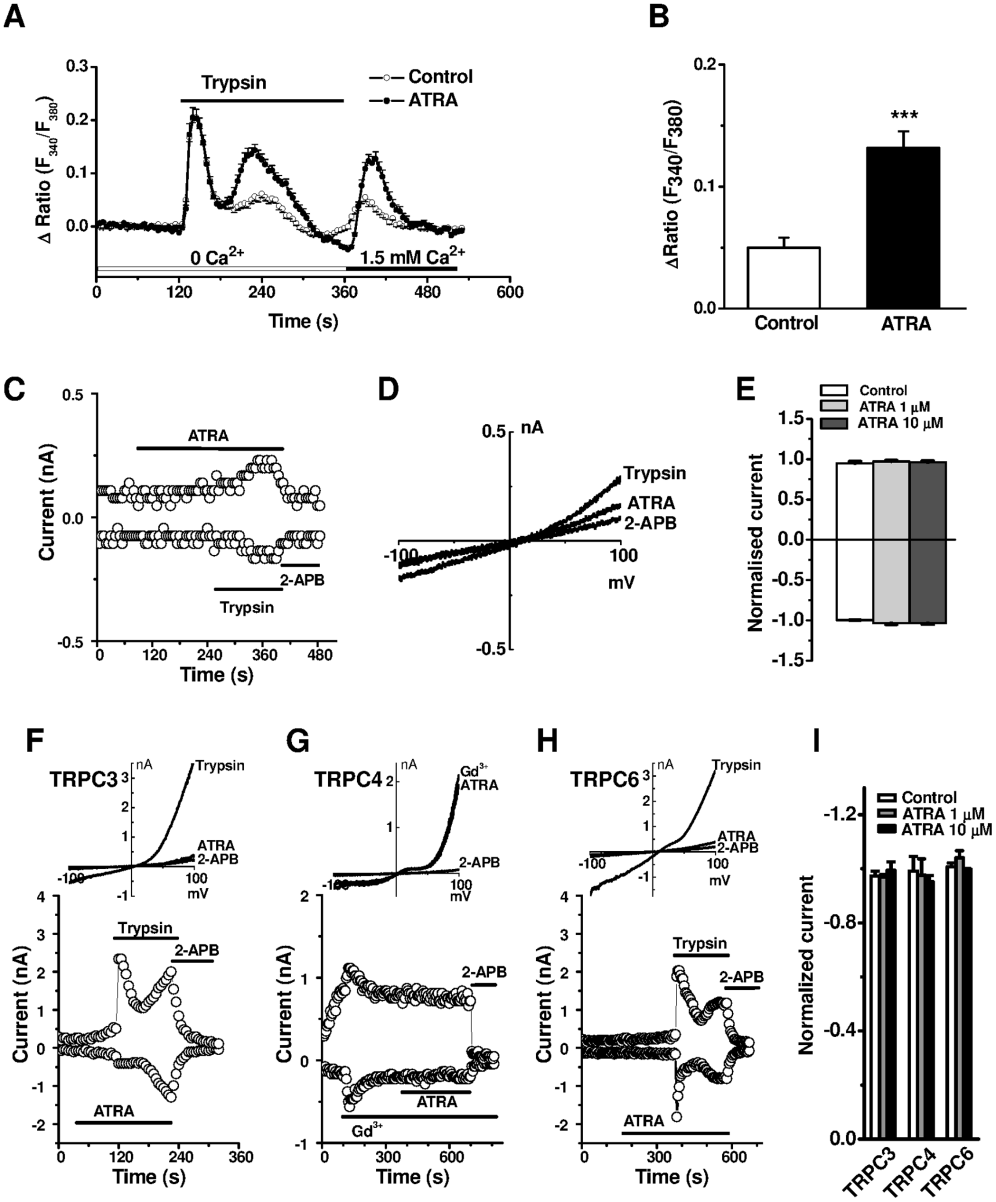
Effect of ATRA on Ca^2+^ influx in A549 cells and TRPC channels. **A,** A549 cells were loaded with 2 µM Fura-PE3/AM and the Ca^2+^ was measured as the ratio (F_340_/F_380_) of Ca^2+^ dye fluorescence. Ca^2+^ release was evoked by trypsin (0.2 nM) and Ca^2+^ influx was introduced by 1.5 mM Ca^2+^ in the perfusion solution. The cells were incubated with 1 µM ATRA for 96 hours and the control cells were incubated with same volume of vehicle. **B,** Mean ± s.e.m. data for the Ca^2+^ entry after the store depletion by trypsin as shown in (A). *n* = 21–32 for each group, *** *P*<0.001. **C,** Whole-cell currents sampled at the command voltage of ±80 mV in A549 cells before and after perfusion with ATRA (1 µM), trypsin (0.2 nM) and 2-APB (100 µM). **D,** *IV* curves for (C). **E,** Mean data for the effect of ATRA. **F–H,** Effect of ATRA on whole-cell currents of TRPC3, TRPC4 and TRPC6 in the HEK293 cells overexpressing individual TRPC isoforms. **I,** Mean ± s.e.m. data measured at −80 mV, and *n = *4–6 for each group.

A549 cells were chronically treated with ATRA (1 µM) for 4 days with every 24-hour refreshment of cell culture medium. The dynamics of intracellular Ca^2+^ was monitored by Fura-PE3/AM. Trypsin at 0.2 nM induced a robust Ca^2+^ release in Ca^2+^ free solution, which was followed by a second Ca^2+^ peak in A549 cells. Perfusion with 1.5 mM Ca^2+^ after the store-depletion with trypsin increased the Ca^2+^ influx in the ATRA-treated cells (Fig. 4A–B), suggesting the chronic treatment with ATRA enhanced the Ca^2+^ influx, but had no effect on the Ca^2+^ release signal. Using whole-cell patch recording, the current of A549 cells was not changed by acute perfusion with ATRA (Fig. 4C–E). To investigate the potential direct effect of ATRA on TRPC channels, the HEK-293 cells inducibly expressing TRPCs were used for whole cell patch recording. The currents of TRPC3, 6 and TRPC4 currents were activated by trypsin or Gd^3+^ (100 µM) respectively. The current-voltage relationship (*IV*) curves for these currents were similar to our previous report [29]. ATRA at 1 µM and 10 µM had no stimulating or blocking effect on these channels, but all these currents were blocked by the TRPC blocker 2-APB, suggesting that ATRA had no acute direct effect on Ca^2+^ current or Ca^2+^ influx through TRPC channels (Fig. 4F–I). The chronic augmentation of Ca^2+^ influx in the ATRA treated A549 cells could be solely contributed by TRPC gene upregulation.

### Cell Differentiation Regulated by TRPC Channel Activity

**Figure 5:**
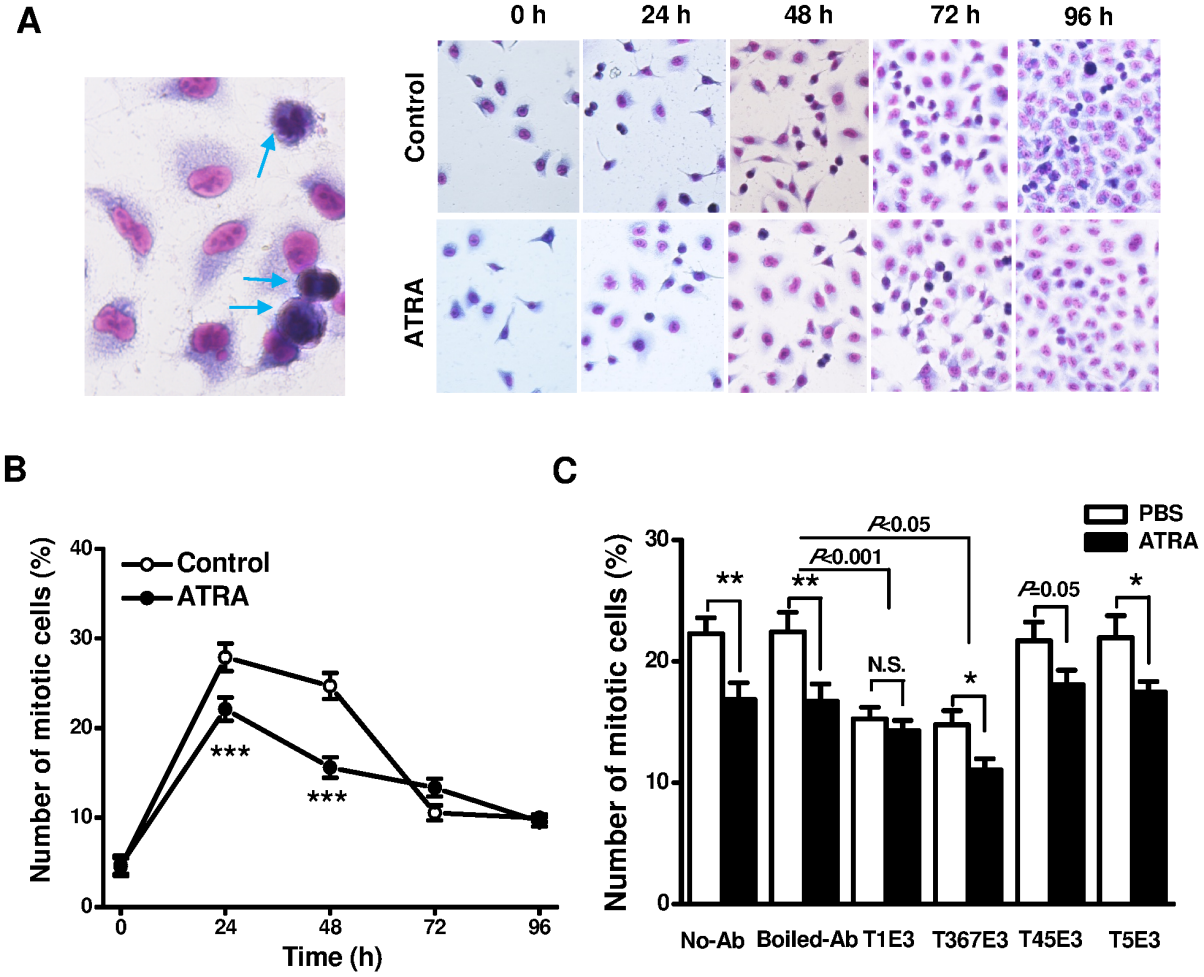
Effect of ATRA on cell mitosis in A549 cells. **A,** Example of Giemsa staining of A549 cells and the treatment with or without ATRA for 0–96 hours. The mitotic cells were indicated by arrow. **B,** Percentage of mitotic cells in control and ATRA-treated groups (*n* = 32–40 microscopic fields for each group containing 6 culture dishes, *** *P*<0.001). **C,** Effect of TRPC channel blocking antibodies on cell mitosis. The medium without antibody (No-Ab) and the boiled antibody (Boiled-Ab) as controls (*n* = 18 microscopic fields from 6 culture wells for each group, N.S: non-significant, * *P*<0.05, and ** *P*<0.01). The delta (Δ) changes of ATRA-induced inhibition were also compared among the boiled-Ab (Control) and blocking antibody treated groups.

The differentiation of A549 lung cancer cells was assessed by Giemsa staining that can visualize chromosomes. Cell with nuclear mitosis displayed dark blue nucleus or twin nuclei staining (Fig. 5). ATRA (1 µM) significantly inhibited cell mitosis at 24 hours and 48 hours cell culture. The percentage of mitotic cells became less at 72 hours and 96 hours cell culture and the difference between the two groups showed no significance (Fig. 5B). The loss of statistic difference after 72 hours cell culture could be due to cell contact inhibition that occurred in the confluent cells after 3 to 4 days cell culture. Therefore, we assessed the effect of TRPC channel blockers on mitosis within 48-hour culture. The specificity and function of the TRPC pore blocking antibodies have been demonstrated in the previous studies [9], [31], [32], [33]. Inhibition of TRPC1, TRPC3 and TRPC6 channels by T1E3 and T367E3 antibodies significantly inhibited the mitosis, while the effects of T1E3 and T367E3 showed less effective after treatment with ATRA comparing to the groups treated with the boiled antibody or the group without addition of antibody.

### TRPC Channel and A549 Cell Proliferation

**Figure 6:**
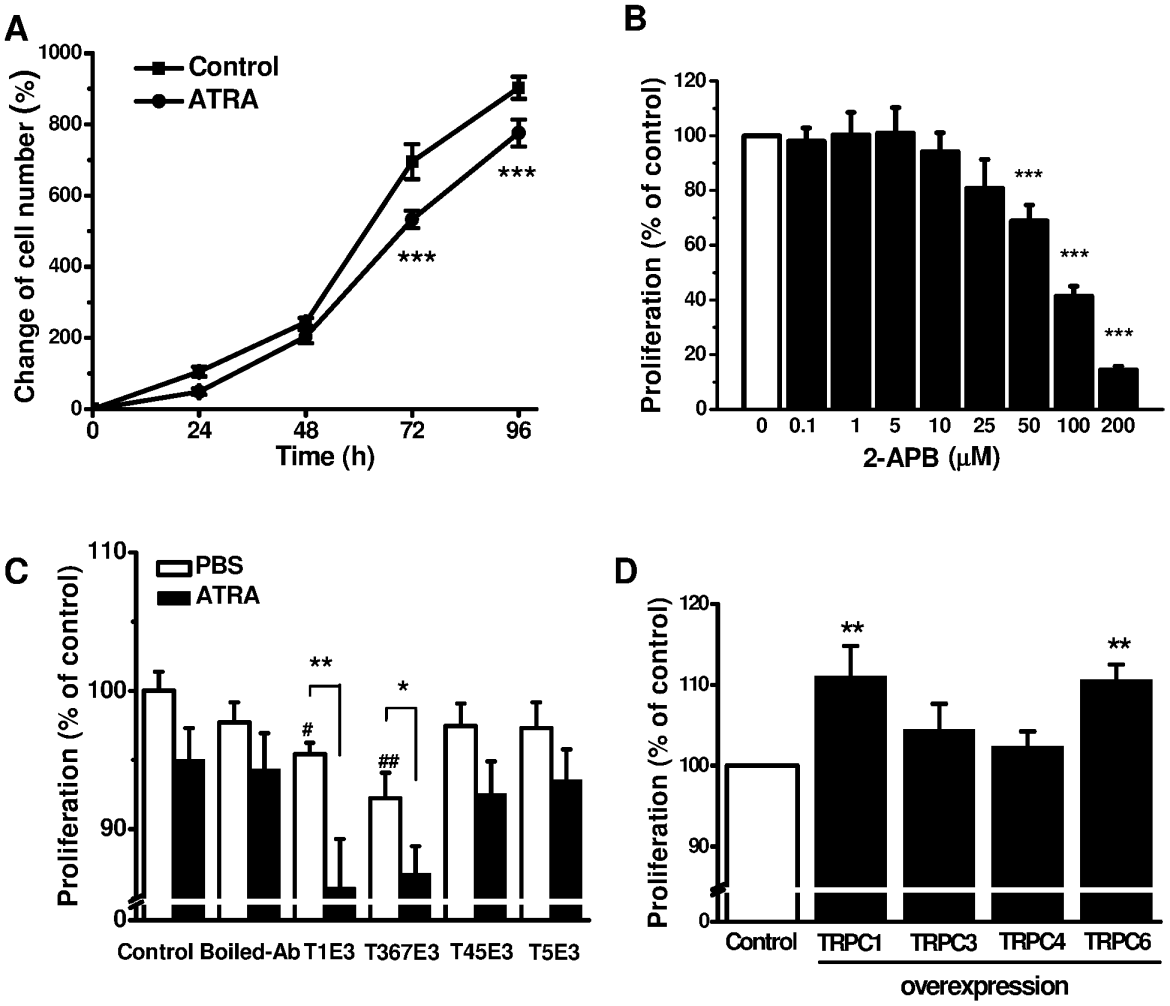
Role of TRPC channels in A549 cell proliferations and the effect of ATRA. **A,** the time course for the effect of ATRA on A549 cell proliferation. **B,** A549 cells were incubated with 2-APB at different concentrations for 24 hours (*n* = 8 wells for each groups) and the cell proliferation was assayed by WST-1 cell proliferation assay kit. **C,** A549 cells treated with specific E3-targeting TRPC blocking antibodies or combination with ATRA for 48 hours (*n* = 6–7 for each group; comparison with the control (^#^); * or ^#^ *P*<0.05, ** or ^##^ *P*<0.01). **D,** A549 cell proliferation was assessed by WST-1 after transfection with TRPC1, 3, 4, and 6 plasmid cDNAs for 48 hours (*n* = 8 for each group, ** *P*<0.01).

Cell differentiation and proliferation are two closely related cellular processes, therefore we also observed effect of TRPC channel activity on A549 cell proliferation. The cell number was increased by ∼ 8-folds after 96-hour cell culture. ATRA (1 µM) significantly inhibited the cell proliferation after 72-hour and 96-hour cell culture (Fig. 6A), suggesting the effect of ATRA on cell proliferation is a slow-onset process. However, the anti-proliferative effect by blocking TRPC channel activity was much faster and the significant difference was achieved within 24 hours after incubation with 2-APB, a non-selective TRPC channel blocker. The EC_50_ for 2-APB was 87.9 µM (Fig. 6B). Using the blocking antibodies T1E3 and T367E3 to specifically block TRPC1 and TRPC3/6 in A549 cells also inhibited the proliferation of the cells without ATRA treatment, but the inhibitory effect was more pronounced when the cells were treated with 1 µM ATRA for 48 hours (Fig. 6C), suggesting the activity of TRPC channel may be augmented by ATRA. To further demonstrate the involvement of TRPC channels in lung cancer cell proliferation, A549 cells were transfected with TRPC1, 3, 4, and 6 plasmid cDNAs. The cell proliferation was increased in the A549 cells overexpressing TRPC1 and TRPC6, but not significant for the cells overexpressing TRPC3 and TRPC4 (Fig. 6D). These results indicate that lung cancer cell proliferation is regulated by the activity of TRPC channels.

## Discussion

In this study, we have shown that TRPC1, TRPC3, TRPC4 and TRPC6 exist in human lung cancer including the adenocarcinoma, squamous cell carcinoma and the adenocarcinoma-derived cell line A549. The expression level of TRPC channels is correlated to the differentiation grade of lung cancer. ATRA upregulates TRPC gene expression in A549 cells. Inhibition of TRPC channel activity shows antiproliferative effect. These findings are important for understanding the roles of TRPC channels in lung cancer cell differentiation and proliferation.

TRPC channels have been detected in many tissues [32], [34], [35], [36] including cancer tissues, such as breast cancer [37], ovarian cancer [9], [20], hepatoma [12], prostate cancer [13], basal cell carcinoma [21], renal cell carcinoma [15], malignant gliomas [16], glioblastoma [8], and gastric tumors [19]. The expression level of each TRPC isoform in different type of cancer cells or tissues are variable, suggesting the contribution or biological importance of each TRPC isoform in different cancer type could be different. For example, the expression of TRPC7 is negative in A549 and SKOV-3, but positive in HepG2 cells in this study and in the differentiated neuroblastoma cells [17]. Moreover, the alternative spliced variants may also contribute to the functionality of TRPC channels, such as TRPC1 and TRPC4 spliced isoforms in ovarian cancer SKOV3 cells [9]. In addition, the expression level of TRPC channels may depend on the differentiation status of the cancer cells, because we found the mRNA expression of TRPC1, TRPC3, TRPC4 and TRPC6 in the low differentiation grade lung cancer was lower than that in the well differentiated cancer, which is consistent to our observation in ovarian cancer [9] and the low level expression of TRPC4 and TRPC6 in immature stem cells [38]. Although our data showed that the mRNA levels of TRPCs in normal lung were higher than that in lung cancer, it is difficult to do such comparison due to variation of cell types, because the normal lung samples contains much more non-epithelial cells than that in the solid tumour samples, such as vascular cells, alveolar macrophages, fibroblasts, and blood cells. The immunostaining on the lung cancer tissue microarrays showed less significant, which could be due to the low sensitivity of methodology comparing to the high sensitivity of real-time PCR. On the other hand, the relationship of TRPC expression with cell differentiation was further confirmed in the *in vitro* lung cancer cell culture model by application of the cell differentiation regulator ATRA, which significantly enhances the expression of TRPC3, 4, and 6. However, the TRPC5 and TRPC7 were still undetectable after ATRA treatment. The TRPC1 mRNA level was not significantly changed by ATRA treatment in the A549 cells, which is inconsistent with the changes of protein expression in the immunostaining data. This could be due to the cell line used in the study. Other lung cancer cell lines should be considered to be used in the future studies.

ATRA has been used as a chemopreventative agent in a variety of tumours. In patients with advanced non-small-cell lung cancer, the addition of ATRA to chemotherapy could increase response rate and survival [39]. In our *in vitro* studies, ATRA at 1 µM slightly inhibited cell proliferation after 3–4 days of cell culture, but had no significant short-term (1–2 days of culture) effect. Unlike the sensitivity of ATRA in acute promyelocytic leukemia, lung cancer A549 cells have less sensitivity to ATRA and the significant inhibition on proliferation only happened at higher concentrations, which is similar to the previous observations [40], [41]. However, after chronic treatment with ATRA, the A549 cell proliferation was more sensitive to TRPC channel blockers, suggesting the upregulation of TRPC channels could lead to enhanced Ca^2+^ influx via TRPC channels, and thus the inhibitory effect of channel blockers on cell proliferation is augmented.

The role of TRPC channels in cancer growth has been demonstrated in prostate cancer and ovarian cancer [9], [13]. Using isoform-specific blocking antibodies, we found that the blockade of TRPC1, TRPC4 and TRPC6 channels causes a significant inhibition of A549 cell proliferation, suggesting that TRPC1, TRPC4 and TRPC6 are important for lung cancer cell proliferation. The specificity of these antibodies used in this study has been well demonstrated and confirmed by ELISA, western blotting, *in vitro* functional testing on Ca^2+^ influx and FACS-based assay [9], [31], [32], [33] and confirmed by other groups [42], [43]. On the contrary, over-expression of TRPC1 and TRPC6 increased the A549 proliferation, however the effect of overexpression of TRPC3 and TRPC4 has not achieved significance in the lung cancer cells. Inhibition of TRPC channel activity could arrest the cell cycle progression through G2/M to G1, which we have recently demonstrated in ovarian cancer SKOV cells [9], whilst ATRA treatment leads to G0/G1 arrest in mouse embryonic palatal mesenchymal cells and cancer cells [44], [45]. Therefore, the antiproliferative effect of ATRA and TRPC channel blockers in A549 cells could be explained as a synergistic action through two different phases in the cell cycle. Upregulation of TRPCs by ATRA may partially offset its anti-proliferative action, which may give a new explanation of ATRA resistance in some cancer therapy. Therefore, combined use of TRPC blockers with ATRA could be a more effective approach for anticancer therapy.

Other TRP subfamilies have also been demonstrated in the cancer development, such as TRPM1 in melanoma [46], TRPM7 in human retinoblastoma cells [47] and human head and neck carcinoma cells [48], TRPM8 and TRPV6 in prostate cancer [49]. The store-operated channel molecules, such as STIM and ORAI proteins may also be important in the regulation of cancer growth though Ca^2+^ signalling pathways [50].

In conclusion, we found that TRPC1, TRPC3, TRPC4 and TRPC6 channels are expressed in lung cancer. The mRNA expression level is related to the cancer differentiation grade and the ATRA treatment. Inhibition of channel activity or reduction of channel expression leads to anti-proliferative effect. Therefore, the regulation of TRPC channels could be a new aspect of the pharmacology of ATRA and the channels could be considered as new potential targets for lung cancer therapy.

## Supporting Information

Table S1 **Primer sequences.**
(DOC)

Table S2 **Analysis of TRPC mRNA expression in the patients with lung cancer.**
(DOCX)
