# Correction: Involvement of TRPC Channels in Lung Cancer Cell Differentiation and the Correlation Analysis in Human Non-Small Cell Lung Cancer

**DOI:** 10.1371/journal.pone.0315242

**Published:** 2024-12-05

**Authors:** Hong-Ni Jiang, Bo Zeng, Yi Zhang, Nikoleta Daskoulidou, Hong Fan, Jie-Ming Qu, Shang-Zhong Xu

The Fig 1A Normal TRPC3 panel is incorrect. An updated Fig 1 presenting the correct panel and the original data underlying Fig 1 (S1 and S2 Files) are provided with this notice.

**Fig 1:**
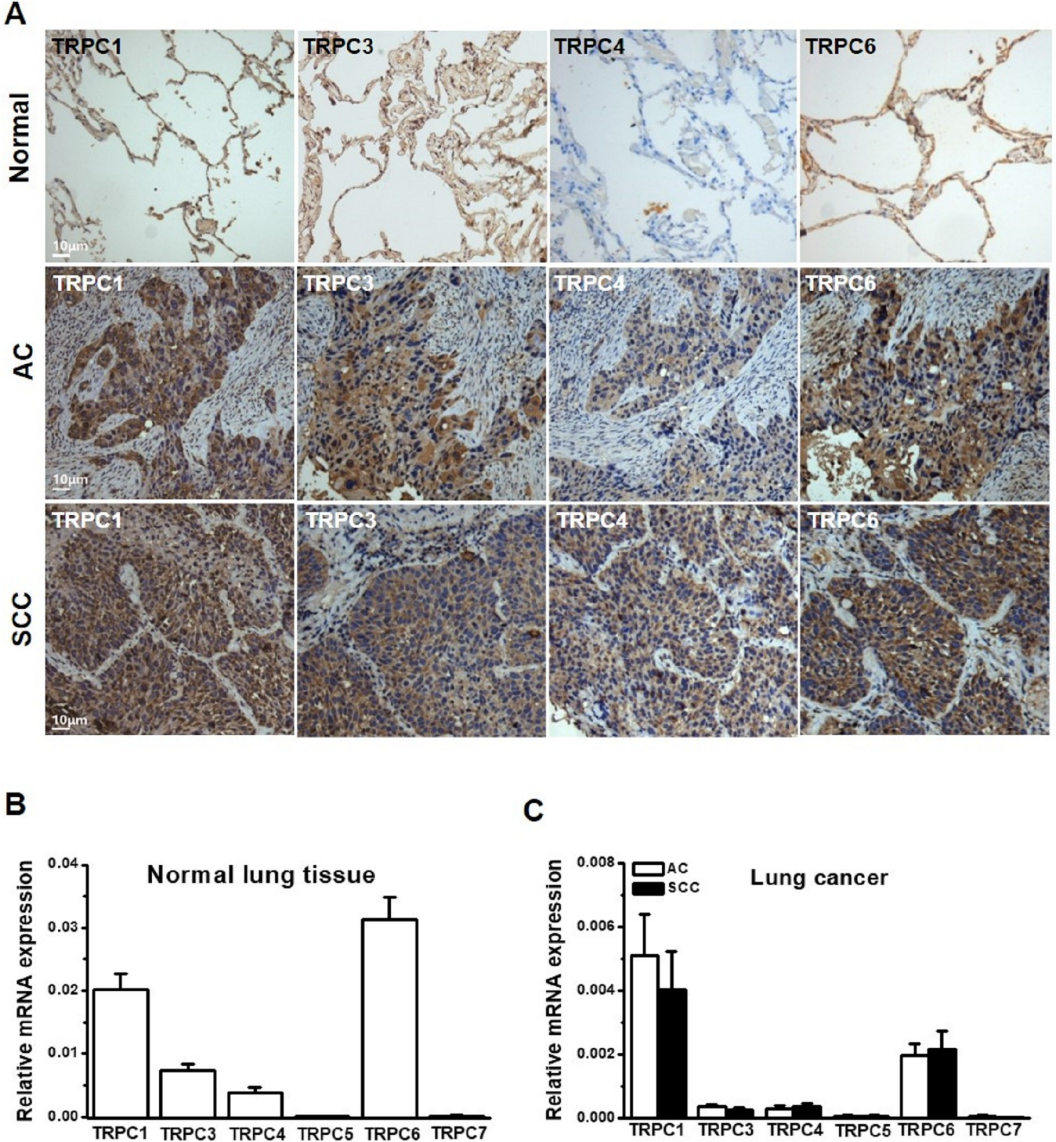
Distribution of TRPC isoforms in human lung and lung cancer. **A,** Examples of human normal lung (*n*  =  20) and lung cancer tissue sections (*n*  =  28) including adenocarcinoma (AC) and squamous cell carcinoma (SCC) were stained with anti-TRPC1, anti-TRPC3, anti-TRPC4 and anti-TRPC6 antibodies using VECTASTAIN ABC system. The positive staining was shown as brown colour. The nuclei were counter-stained by hematoxylin. **B,** The mRNA was detected by real-time PCR in normal lung tissues using the primers in Table S1. The GAPDH was used as internal house-keeping gene control for quantification (*n*  =  25 patients for TRPC1, 4, 5 and 6 groups; *n*  =  24 for TRPC3; and *n*  =  9 for TRPC7). **C,** The mRNA levels in lung cancer tissues (AC: *n*  =  9–15; SCC: *n*  =  8–11).

The Fig 2 “11C” panel (top right) is incorrect. An updated Fig 2 presenting the correct panel, and the original data underlying Fig 2 (S3 and S4 Files) are provided with this notice. The authors clarify that the purpose of the image in Fig 2C is to show the paradigm of the preparation of the tissue chip sections only, and clarify that the table presented in Fig 2C is not entirely derived from the images of the tissue microarrays shown in Fig 2C.

**Fig 2:**
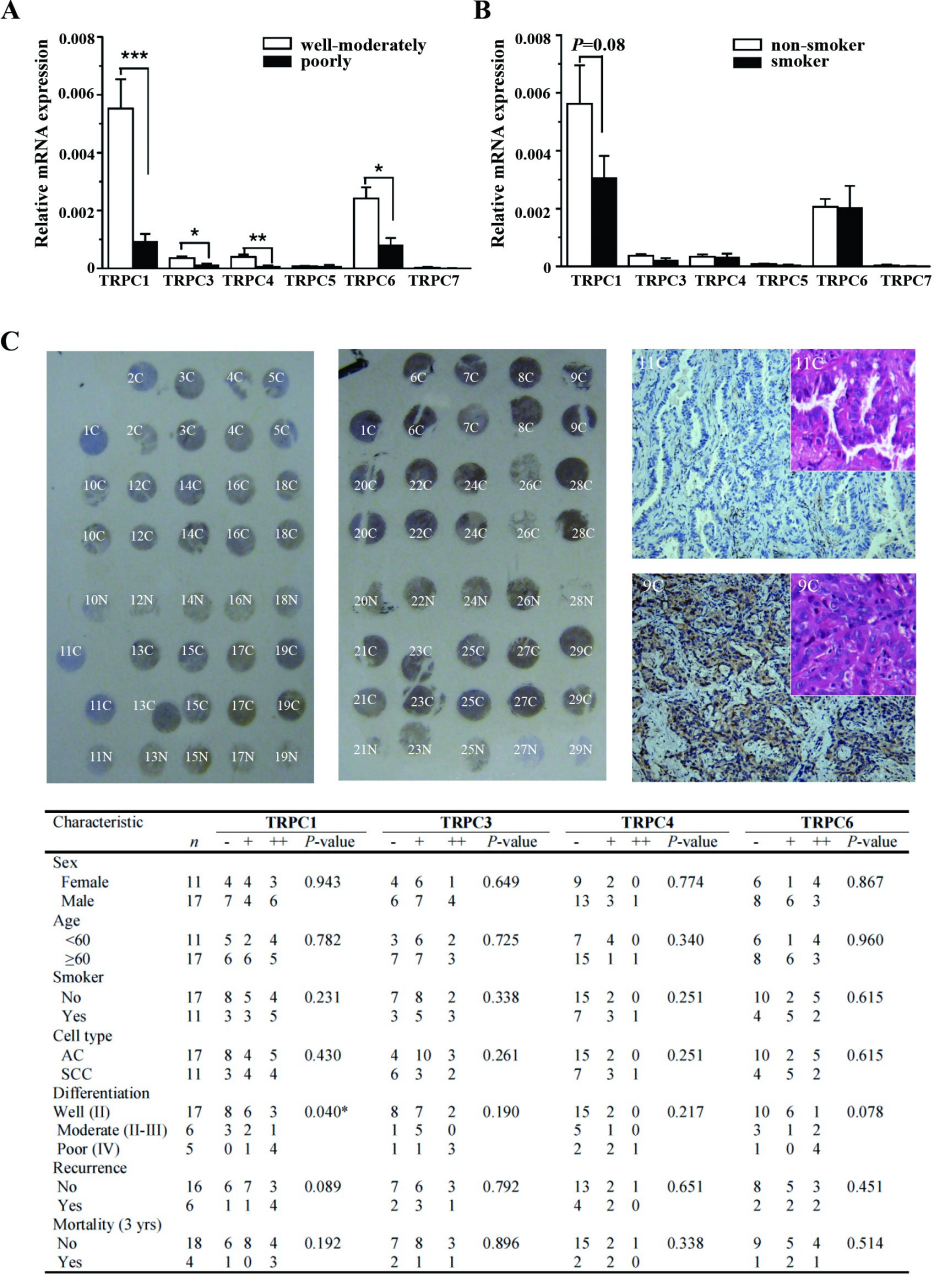
Correlation of TRPC expression to differentiation grade, smoking, cell type, sex and age determined by real-time PCR and immunostaining. **A,** The mRNA expression of TRPCs in lung cancer tissues with well-moderate (grade II (*n*  =  17) and grade III (*n*  =  6)) or poor (grade IV, *n*  =  5) differentiation grade was detected by real-time PCR. GAPDH was used as housekeeping gene control. **B,** The mRNA expression of TRPCs in the lung cancer tissues obtained from smoker (20 cigarettes per day for more than 10 years, *n*  =  11) and non-smoker (*n*  =  17). **C,** Example of two tissue microarrays with normal lung (N) and lung cancer (C) labels were stained with anti-TRPC1 antibody. The cell type and differentiation grade of each section were characterized by HE-staining. The two examples (11C and 9C) with well-differentiated adenocarcinoma were shown in the insets. The correlation of staining intensity to other factors was shown in the table and the significance was assessed by Ridit analysis. * *P*<0.05, ** *P*<0.01, *** *P*<0.001.

Fig 3A was prepared using spliced data from different blots. An updated Fig 3 presenting the Fig 3A results alongside the correct marker lanes and with gel splicing clearly marked and the original data underlying Fig 3 (S5–S7 Files) are provided with this notice.

**Fig 3:**
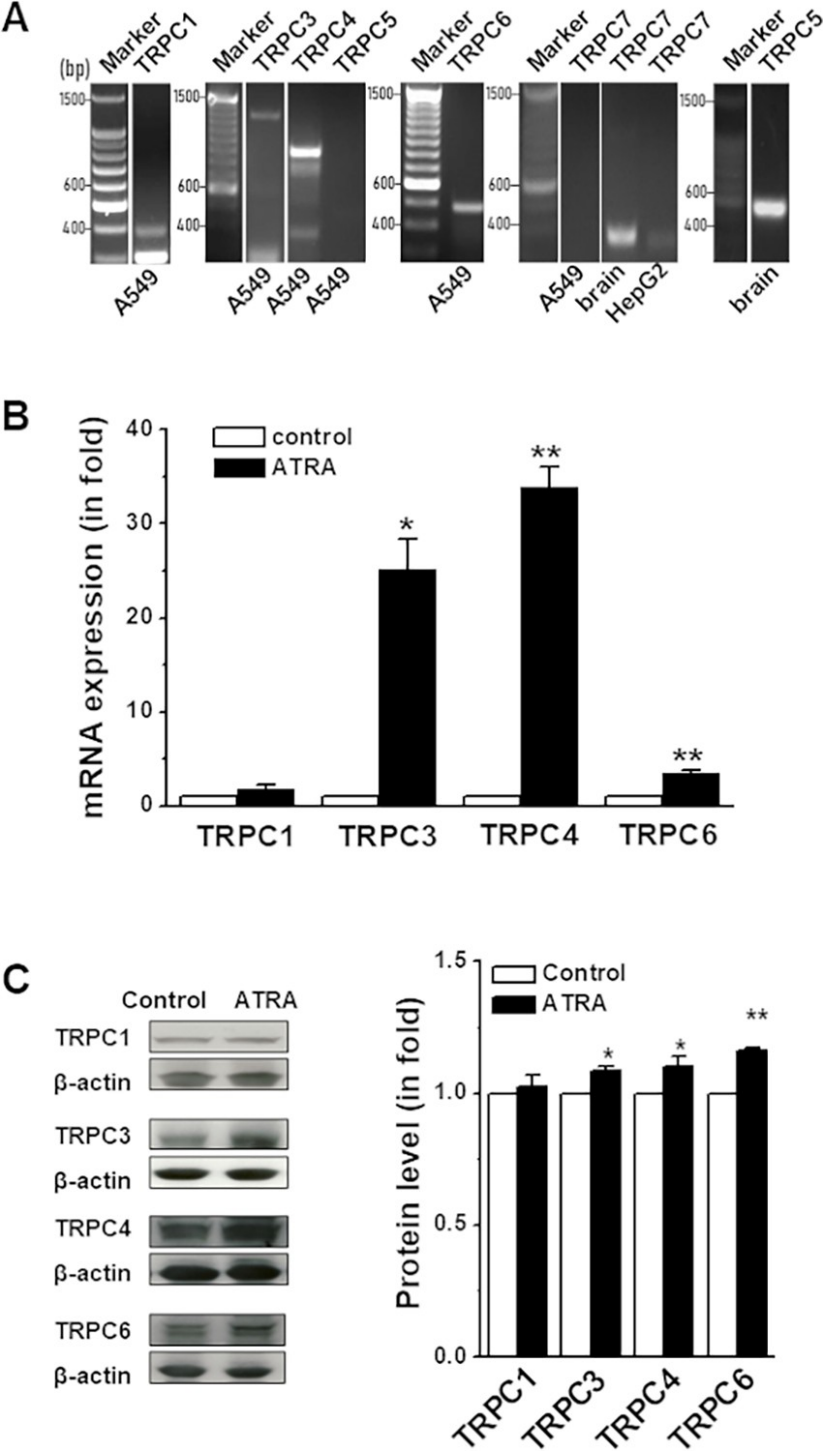
Upregulation of TRPCs by ATRA in A549 cells. **A,** The mRNAs of TRPC1, 3, 4 and 6 were detected in A549 cells by RT-PCR using the primer set in Table S1. The PCR bands for TRPC5 and TRPC7 were negative in A549 cells, but positive in human brain or HepG2. **B,** The mRNA was detected by real-time PCR in the A549 cells treated with all-*trans* retinoic acid (ATRA, 1 µM) for 96 hours. The β-actin was used as a housekeeping gene for relative quantification. The 2^(−ΔΔCt)^ method was used for calculation. **C,** The TRPC proteins were quantified by Western blotting using anti-TRPC1 (T1E3), anti-TRPC3, anti-TRPC4 (T45E3) and anti-TRPC6 antibodies. Anti-β-actin antibody was used for relative protein quantification (*n*  =  3 independent experiments and each experiment with triplicate samples).

## Supporting information

S1 File Image data underlying the Fig 1A results. (ZIP)

S2 File Individual level data underlying the Fig 1B results. (ZIP)

S3 File Image data underlying the Fig 2C results. (ZIP)

S4 File Individual level data underlying the Fig 2A and Fig 2B results. (ZIP)

S5 File Image data underlying the Fig 3A results. (JPG)

S6 File Image data underlying the Fig 3C results. (PPTX)

S7 File Individual level data underlying the Fig 3B and Fig 3C results. (ZIP)
