# Supplementary material for: Involvement of TRPC Channels in Lung Cancer Cell Differentiation and the Correlation Analysis in Human Non-Small Cell Lung Cancer
Source: PLoS One. 2013 Jun 28;8(6):e67637. doi: 10.1371/journal.pone.0067637 (PMC3695899; doi:10.1371/journal.pone.0067637)
Supplement: Table S1 — Primer sequences. (DOC) [file pone.0067637.s001.doc]

**Table S1. Primer sequences**

| **Gene** |  | **Primers (5’ to 3’)** |
| --- | --- | --- |
| β-actin  (NM_001101.3) | F  R  F  R | ACAGAGCCTCGCCTTTGC  GGAATCCTTCTGACCCATGC  CCACTCCTCCACCTTTGAC  ACCCTGTTGCTGTAGCCA |
| TRPC1  (NM_003304) | F  R  F  R | GATGTGCTTGGGAGAAATGC  CAAGACGAAACCTGGAATGC  ATCAAAAGGCAAGGTCAAACGG  ACAGATCTTGGCGCAGTTCGT |
| TRPC3  (NM_001130698) | F  R  F  R | AACAAGCAAGGGTGACCTTC  GAGGCATTGAACACAAGCAG  GCATTCTCAATCAGCCAACACG  TCCTCAGTTGCTTGGCTCTTGT |
| TRPC4  (NM_016179.2) | F  R  F  R | ATGAGGAACCTGGTGAAGCGATA  GCATTCGCAGATTGTATTGTGGA  CAATGTCATCTCTCTGGTTGTTC  CCTGTAACCCCAGTGTGTCC |
| TRPC5  (NM_012471.2) | F  R  F  R | TGAGAACGAGAACCTGGAG  TACTCGGCCTTGAACTCATTC  TGATCGGCAATAAGCTGATAGG  CAATGTGAAAGCCAGACACGA |
| TRPC6  (NM_004621.5) | F  R  F  R | TACGATGGTCATTGTTTTGC  GATTGAAGGGTACAGGAAGTG  CATTTACTGGTTTGCTCCATGCA  GTGCTGGTTTCATTAGGAAGGAG |
| TRPC7  (NM_020389.1) | F  R  F  R | ATCTTCGTGGCCTCCTTCAC  AACGCTGGGTTGTATTTGGC  CTTACTACCGAGGTGCCAAATACAA  CGCCGTAGAGAACGTAGCCAAT |

Note: F: forward, R: reverse.

Primers for real-time PCR were highlighted in green colour.
