# Supplementary material for: Involvement of TRPC Channels in Lung Cancer Cell Differentiation and the Correlation Analysis in Human Non-Small Cell Lung Cancer
Source: PLoS One. 2013 Jun 28;8(6):e67637. doi: 10.1371/journal.pone.0067637 (PMC3695899; doi:10.1371/journal.pone.0067637)
Supplement: Table S2 — Analysis of TRPC mRNA expression in the patients with lung cancer. (DOCX) [file pone.0067637.s002.docx]

**Table S2:** Analysis of TRPC mRNA expression in the patients with lung cancer

| Characteristic | ***n*** | **TRPC1 mRNA**  ≥Cp† <Cp *P*-value | | | | | ***n*** | | **TRPC3 mRNA**  ≥Cp <Cp *P*-value | | | | **TRPC4 mRNA**  ***n*** ≥ Cp <Cp *P*-value | | | | | | ***n*** | **TRPC6 mRNA**  ≥Cp <Cp *P*-value | | | | | |
| --- | --- | --- | --- | --- | --- | --- | --- | --- | --- | --- | --- | --- | --- | --- | --- | --- | --- | --- | --- | --- | --- | --- | --- | --- | --- |
| Sex |  |  |  |  |  |  |  | |  |  |  |  |  |  | |  |  |  |  |  |  |  | | | |
| Female | 11 | 6 | | 5 | **NS** | |  | 9 | 6 | 3 | NS | |  | 10 | 4 | 6 | NS |  | 10 | 6 | 4 | NS | | |  |
| Male | 17 | 8 | | 9 |  |  |  | 15 | 6 | 9 |  | |  | 15 | 7 | 8 |  |  | 15 | 7 | 8 |  | | |  |
| Age |  |  | |  |  |  |  |  |  |  |  | |  |  |  |  |  |  |  |  |  |  | | |  |
| ≤60 | 14 | 8 | | 6 | NS | |  | 11 | 7 | 4 | NS | |  | 12 | 6 | 6 | NS |  | 12 | 8 | 4 | NS | | |  |
| >60 | 14 | 6 | | 8 |  |  |  | 13 | 4 | 9 |  | |  | 13 | 7 | 6 |  |  | 13 | 5 | 8 |  | | |  |
| Smoker |  |  | |  |  |  |  |  |  |  |  | |  |  |  |  |  |  |  |  |  |  | | |  |
| No | 17 | 9 | | 8 | NS | |  | 14 | 9 | 5 | NS | |  | 15 | 8 | 7 | NS |  | 15 | 10 | 5 | NS | |  |  |
| Yes | 11 | 5 | | 6 |  |  |  | 10 | 3 | 7 |  | |  |  | 5 | 5 |  |  | 10 | 3 | 7 |  | |  |  |
| Cell type |  |  | |  |  |  |  |  |  |  |  | |  |  |  |  |  |  |  |  |  |  | |  |  |
| AC | 15 | 9 | | 6 | NS | |  | 11 | 6 | 5 | NS | |  | 12 | 7 | 5 | NS |  | 12 | 9 | 3 | NS | |  |  |
| SCC | 11 | 5 | | 6 |  |  |  | 11 | 6 | 5 |  | |  | 11 | 6 | 5 |  |  | 11 | 4 | 7 |  | |  |  |
| Differentiation |  |  | |  |  |  |  |  |  |  |  | |  |  |  |  |  |  |  |  |  |  | |  |  |
| Well (II) | 17 | 9 | | 8 | <0.05 | | | 13 | 7 | 6 | NS | |  | 14 | 9 | 5 | <0.05 |  | 14 | 7 | 7 | <0.01 | |  |  |
| Moderate (II-III) | 6 | 5 | | 1 |  |  |  | 6 | 4 | 2 |  | |  | 6 | 4 | 2 |  |  | 6 | 6 | 0 |  | |  |  |
| Poor (IV) | 5 | 0 | | 5 |  |  |  | 5 | 1 | 4 |  | |  | 5 | 0 | 5 |  |  | 5 | 0 | 5 |  | |  |  |
| Recurrence |  |  | |  |  |  |  |  |  |  |  | |  |  |  |  |  |  |  |  |  |  | |  |  |
| No | 16 | 9 | | 7 | NS | |  | 13 | 7 | 6 | NS | |  | 14 | 7 | 7 | NS |  | 14 | 6 | 8 | NS | |  |  |
| Yes  Mortality (3 yrs) | 6 | 4 | | 2 |  |  |  | 6 | 3 | 3 |  | |  | 6 | 3 | 3 |  |  | 6 | 3 | 3 |  |  |  |  |
| No | 18 | 10 | | 8 | NS | |  | 15 | 8 | 7 | NS | |  | 16 | 6 | 10 | NS |  | 16 | 7 | 9 | NS |  |  |  |
| Yes | 4 | 3 | | 1 |  |  |  | 4 | 2 | 2 |  | |  | 4 | 3 | 1 |  |  | 4 | 2 | 2 |  |  |  |  |

Note: Chi-square analysis was used. NS: no significance (*P*>0.05). †Cp: Cut-off point for the mRNA levels. The values were set at the median for each group, i.e., TRPC1 (0.0036), TRPC3 (0.00026), TRPC4 (0.00021) and TRPC6 (0.0018).
