# Supplementary figures and images for: Correction: Involvement of TRPC Channels in Lung Cancer Cell Differentiation and the Correlation Analysis in Human Non-Small Cell Lung Cancer
Source: PLoS One. 2024 Dec 5;19(12):e0315242. doi: 10.1371/journal.pone.0315242 (PMC11620368; doi:10.1371/journal.pone.0315242)

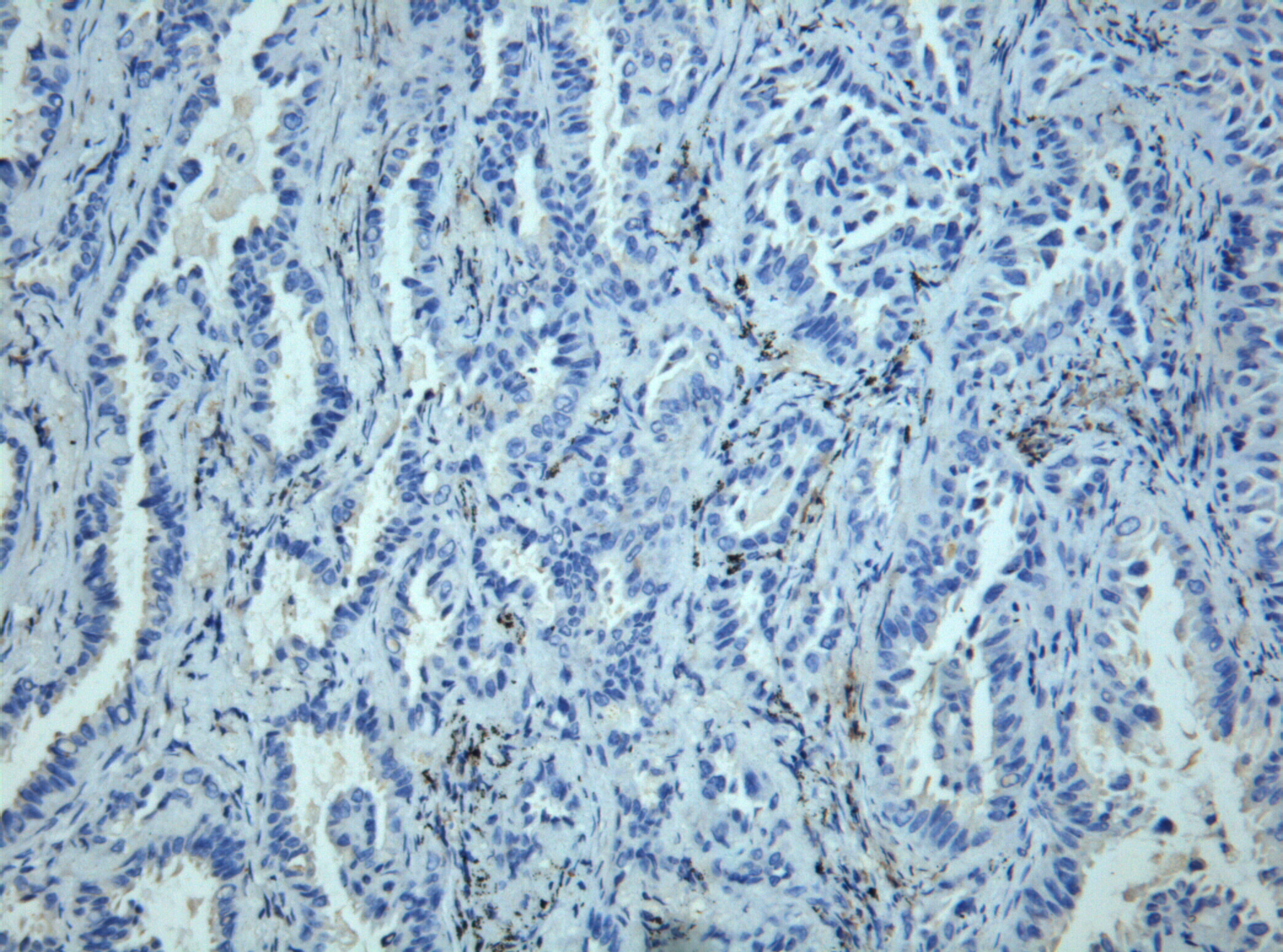

Supplement: S1 File — (ZIP) [file pone.0315242.s001.zip › IHC-TRPC1/11c-.jpg]

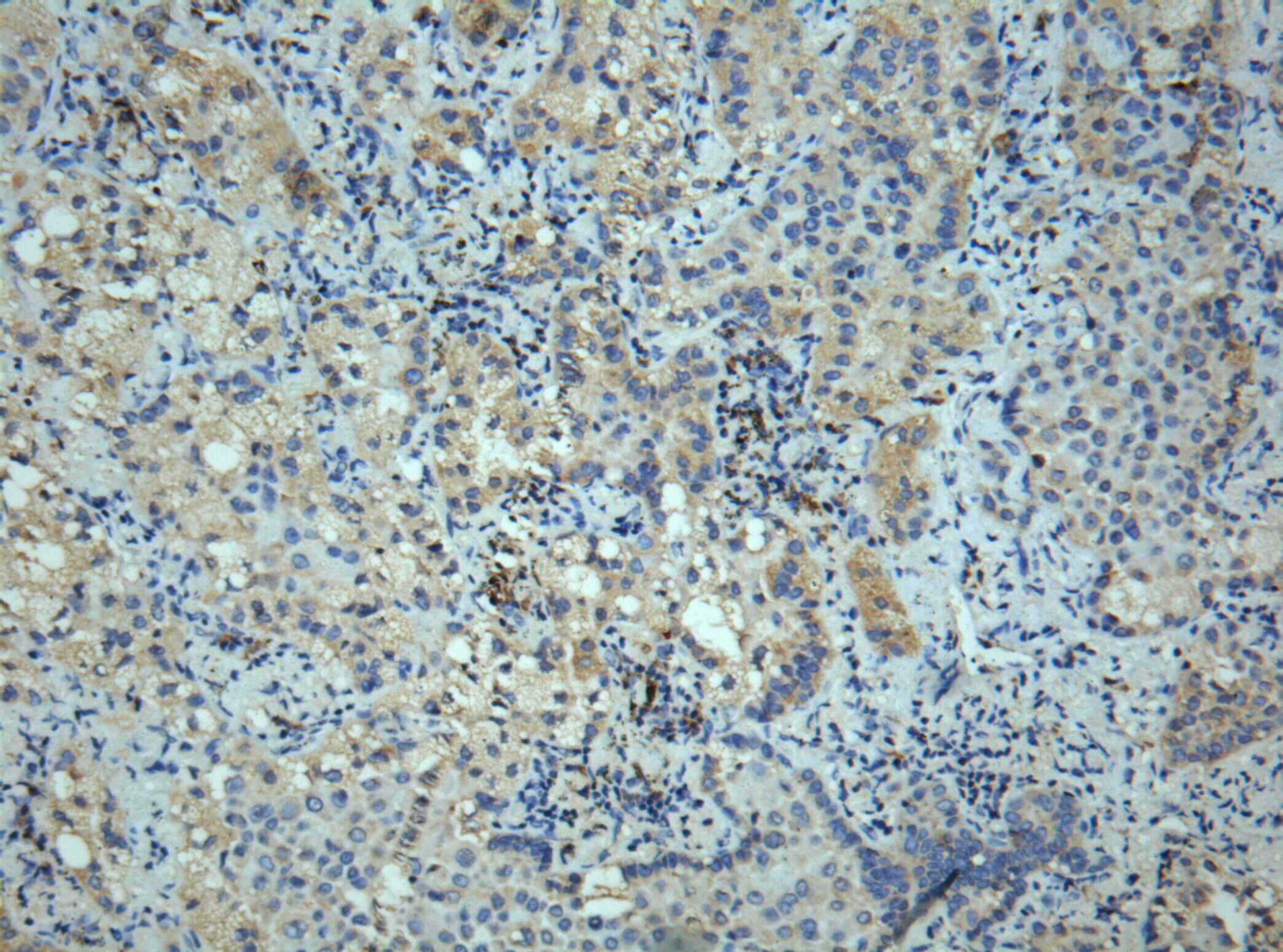

Supplement: S1 File — (ZIP) [file pone.0315242.s001.zip › IHC-TRPC1/12c+.jpg]

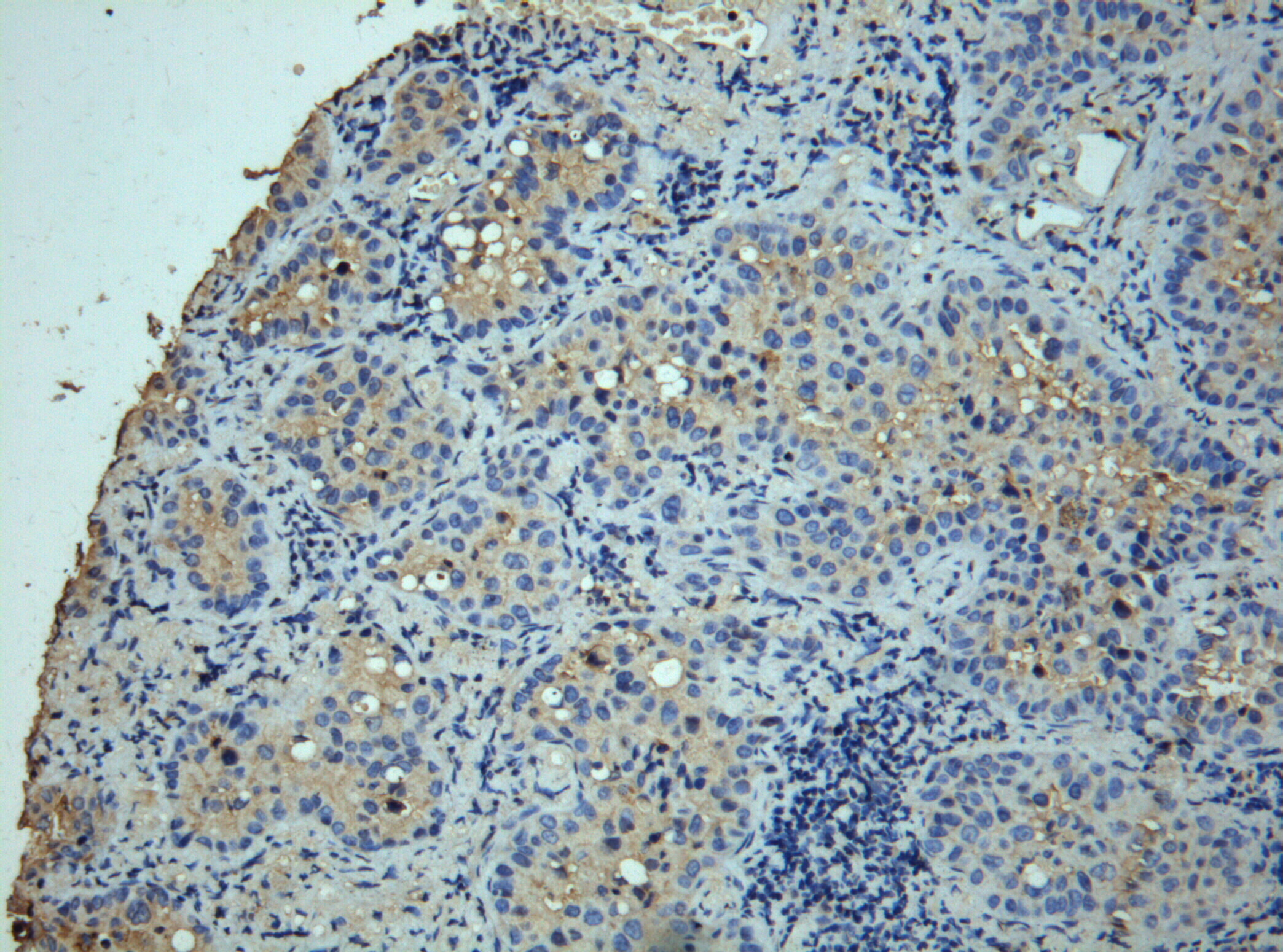

Supplement: S1 File — (ZIP) [file pone.0315242.s001.zip › IHC-TRPC1/13c+.jpg]

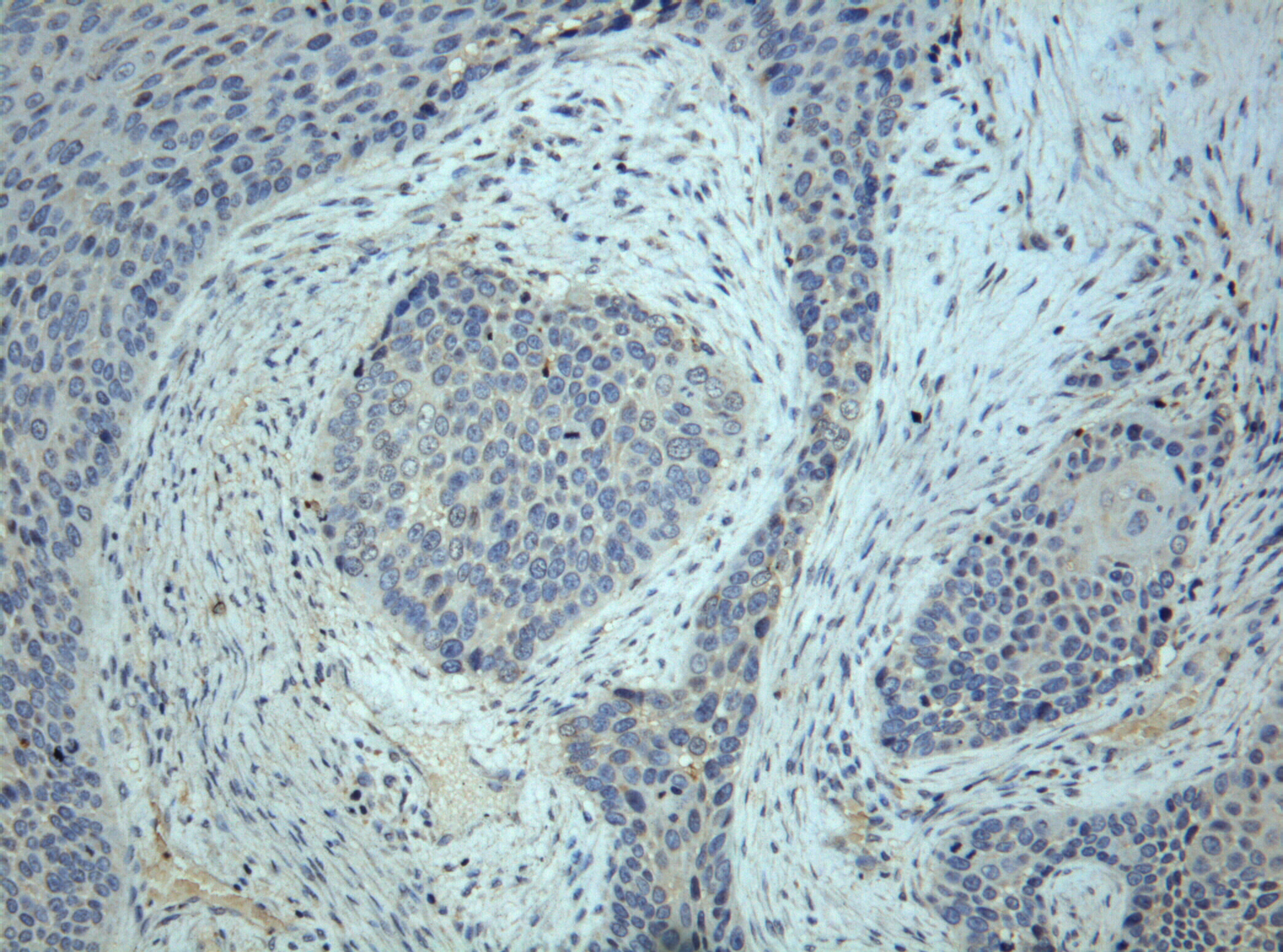

Supplement: S1 File — (ZIP) [file pone.0315242.s001.zip › IHC-TRPC1/15c++.jpg]

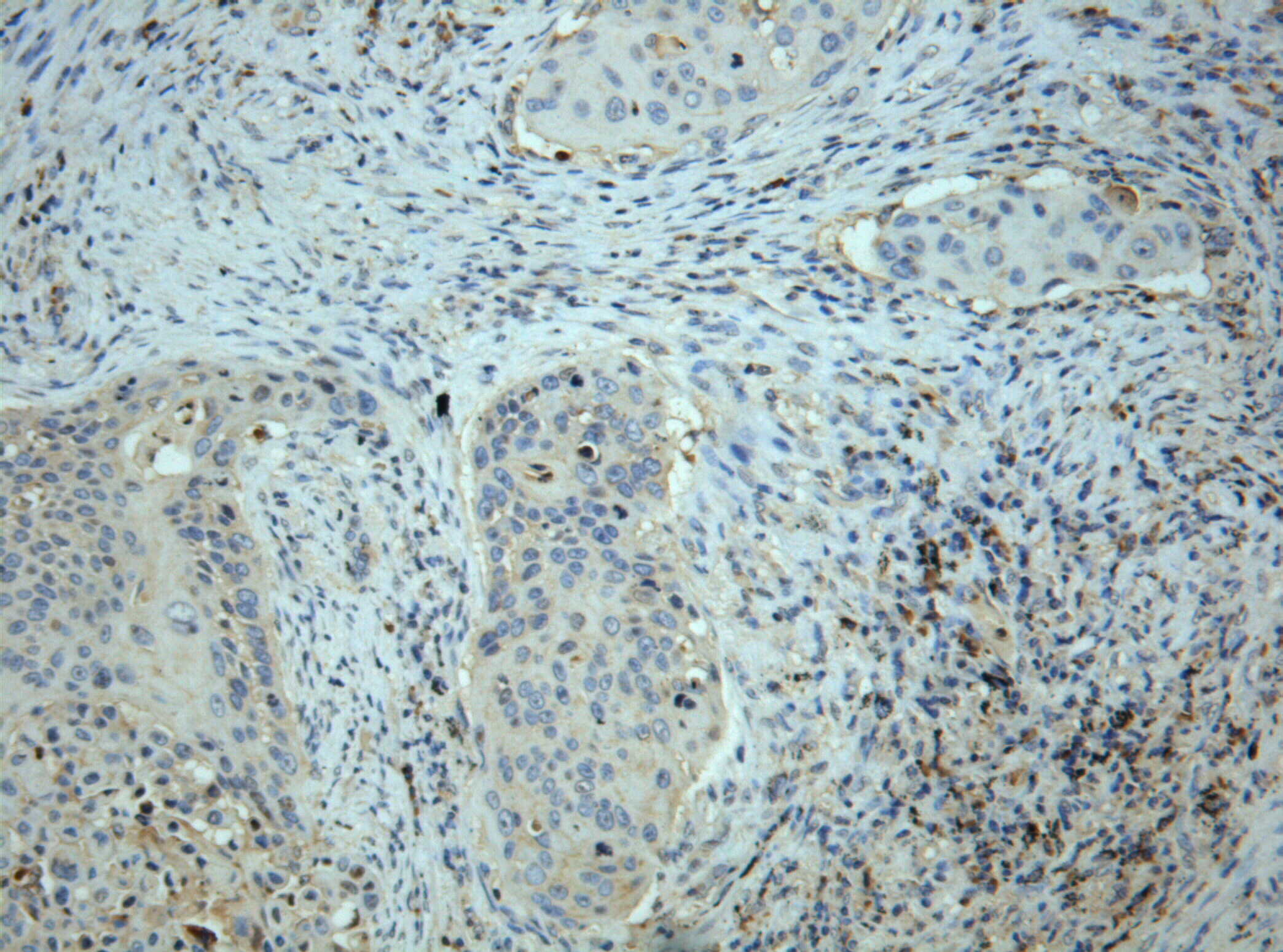

Supplement: S1 File — (ZIP) [file pone.0315242.s001.zip › IHC-TRPC1/16c+.jpg]

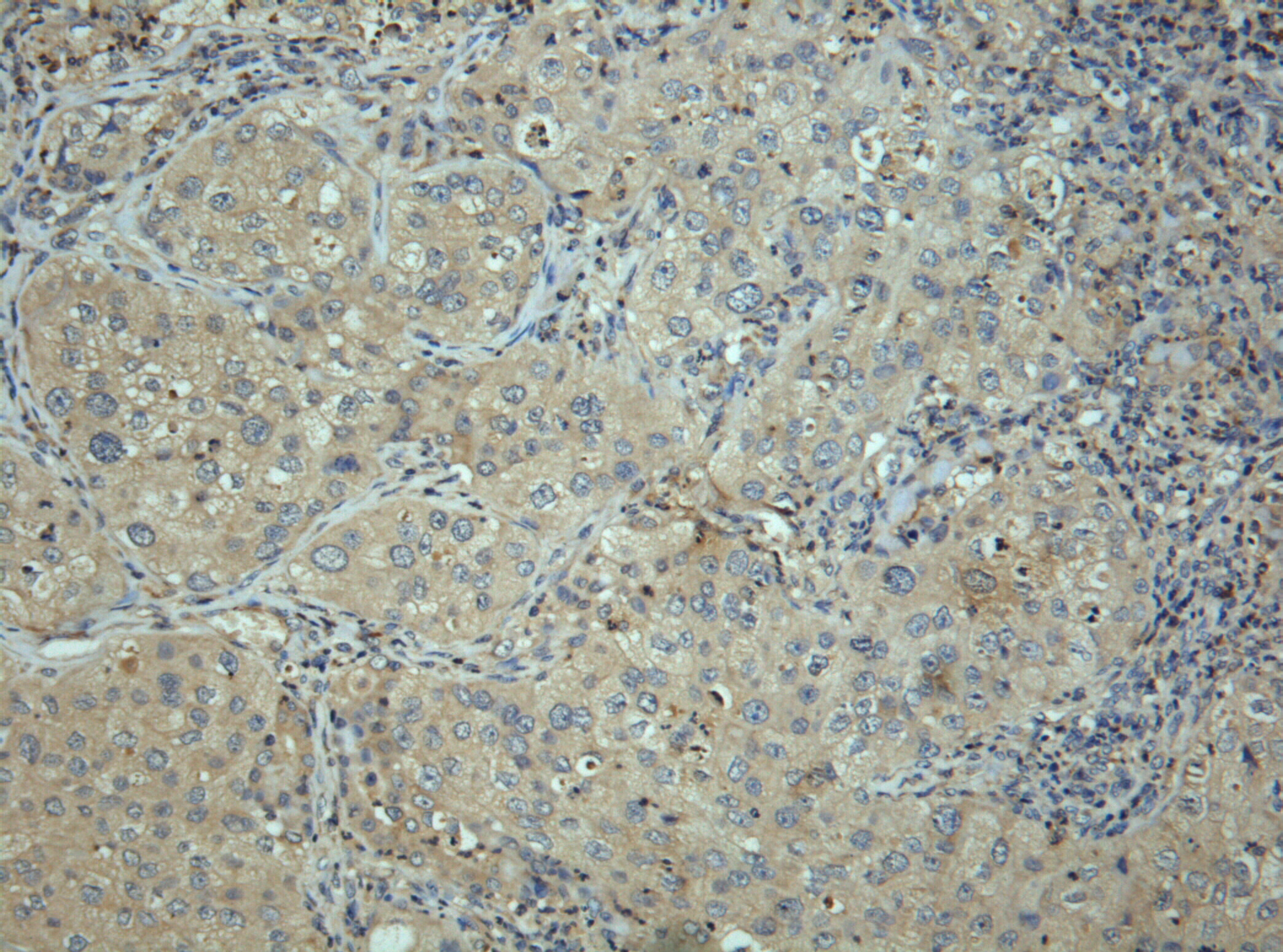

Supplement: S1 File — (ZIP) [file pone.0315242.s001.zip › IHC-TRPC1/17c++.jpg]

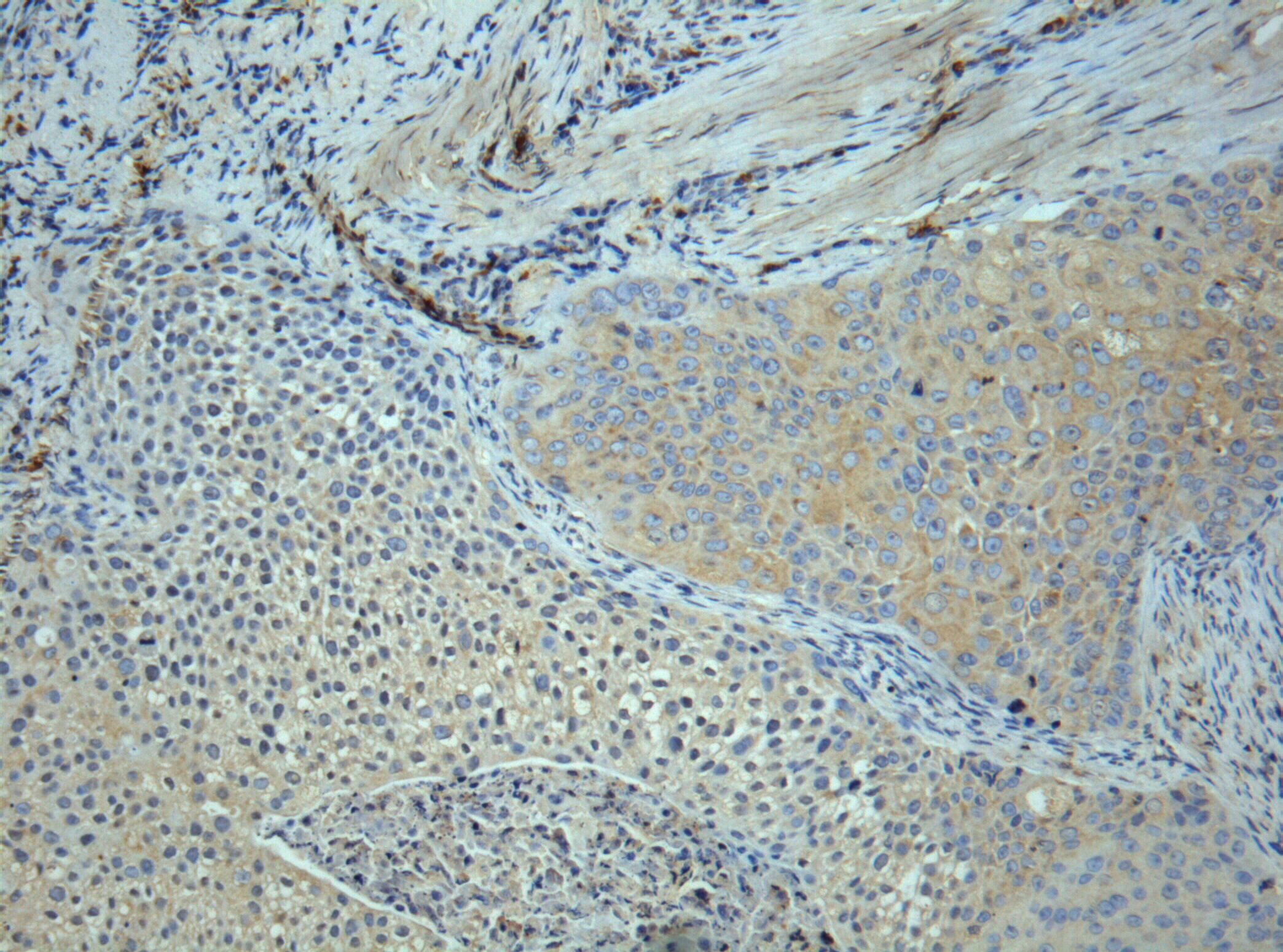

Supplement: S1 File — (ZIP) [file pone.0315242.s001.zip › IHC-TRPC1/18c++.jpg]

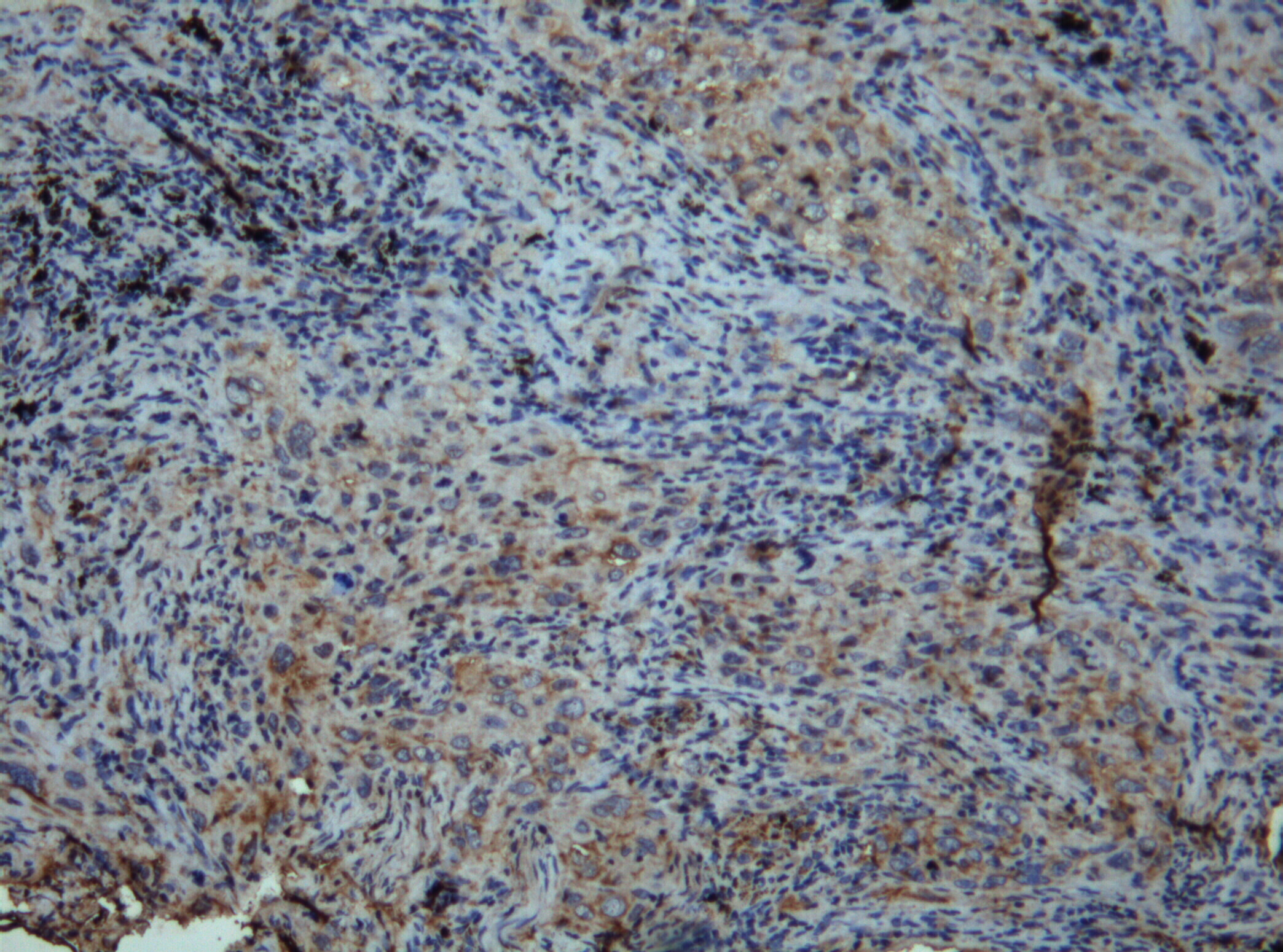

Supplement: S1 File — (ZIP) [file pone.0315242.s001.zip › IHC-TRPC1/20C++.jpg]

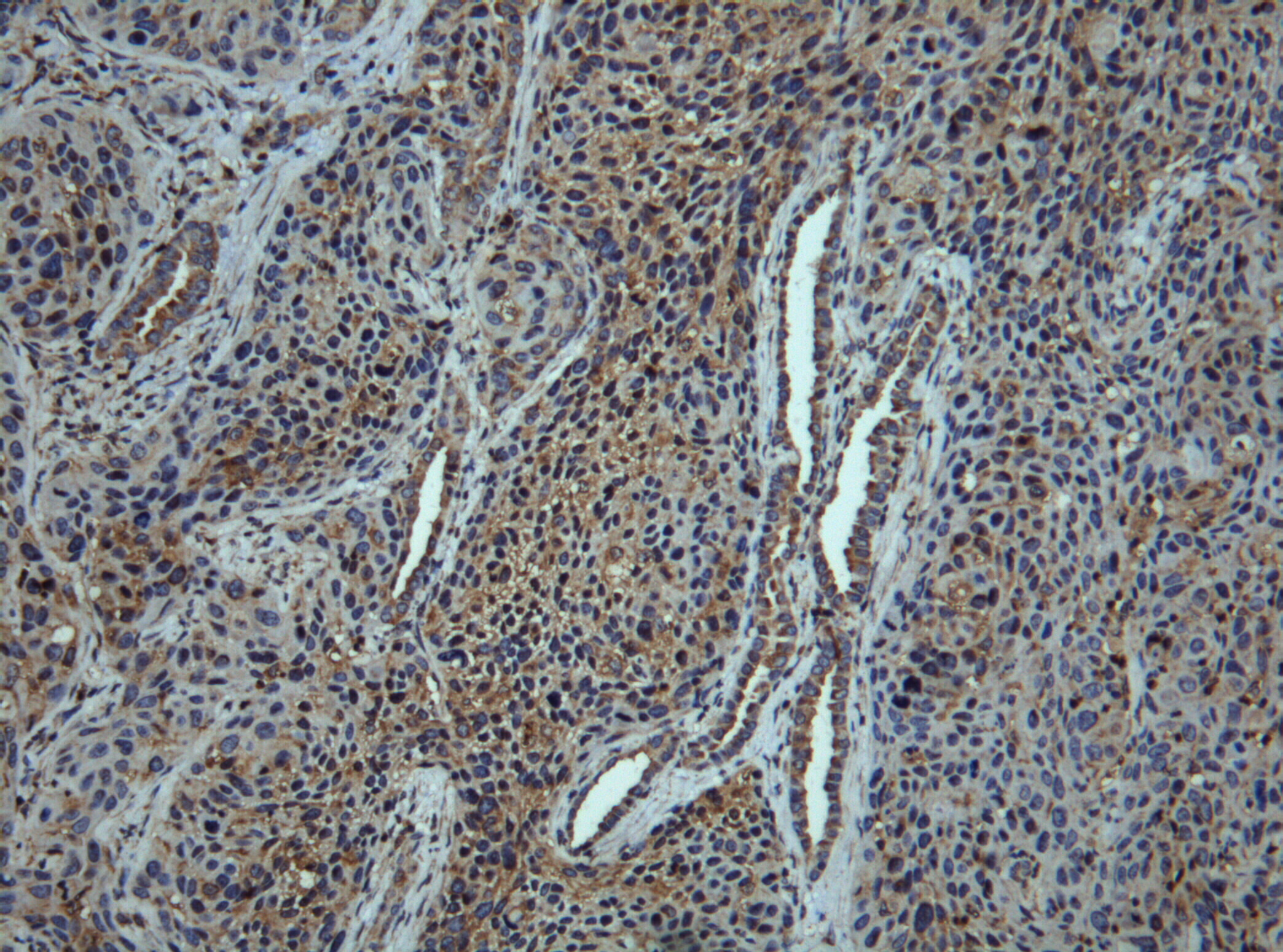

Supplement: S1 File — (ZIP) [file pone.0315242.s001.zip › IHC-TRPC1/21c++.jpg]

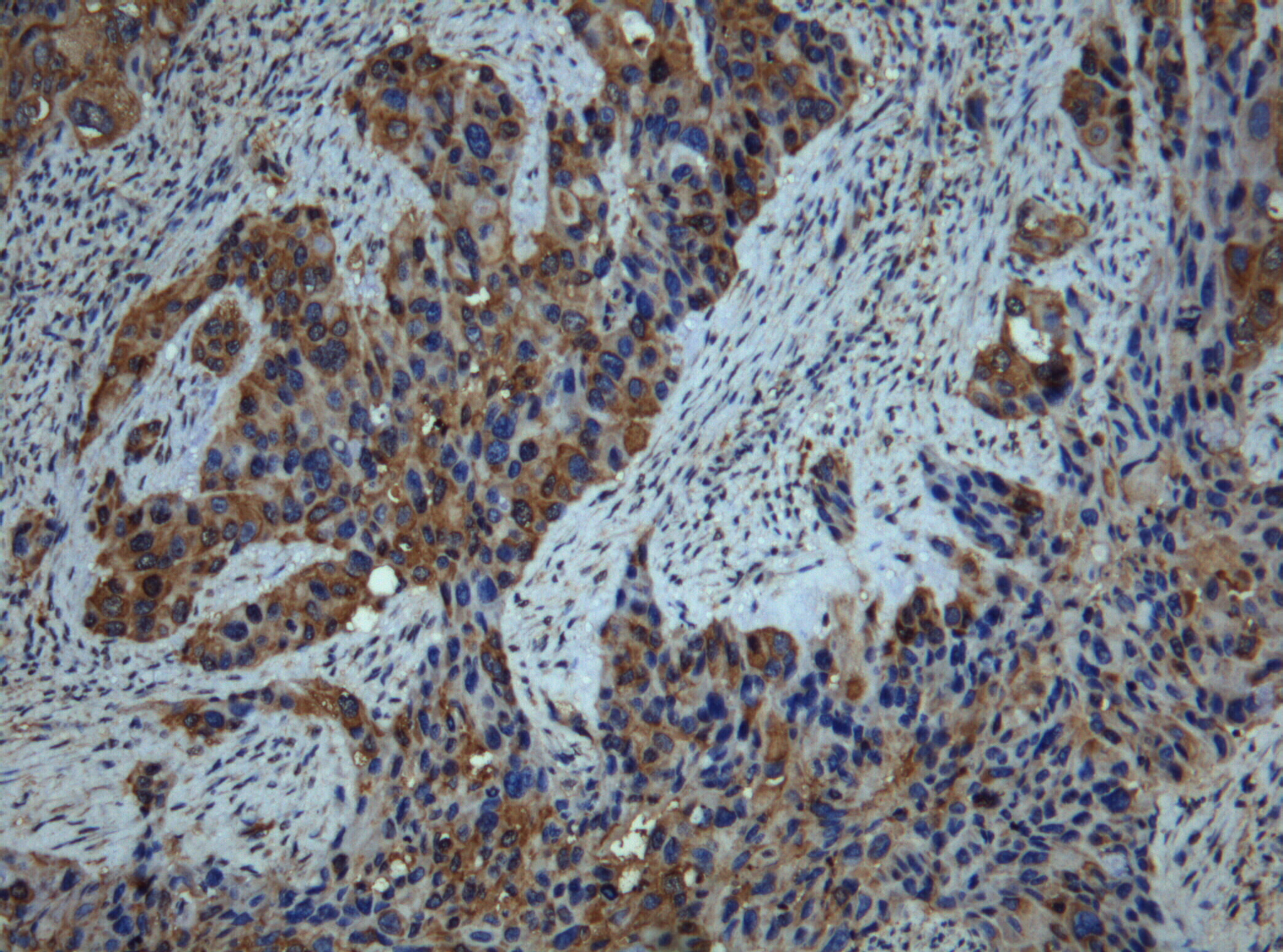

Supplement: S1 File — (ZIP) [file pone.0315242.s001.zip › IHC-TRPC1/22c++.jpg]

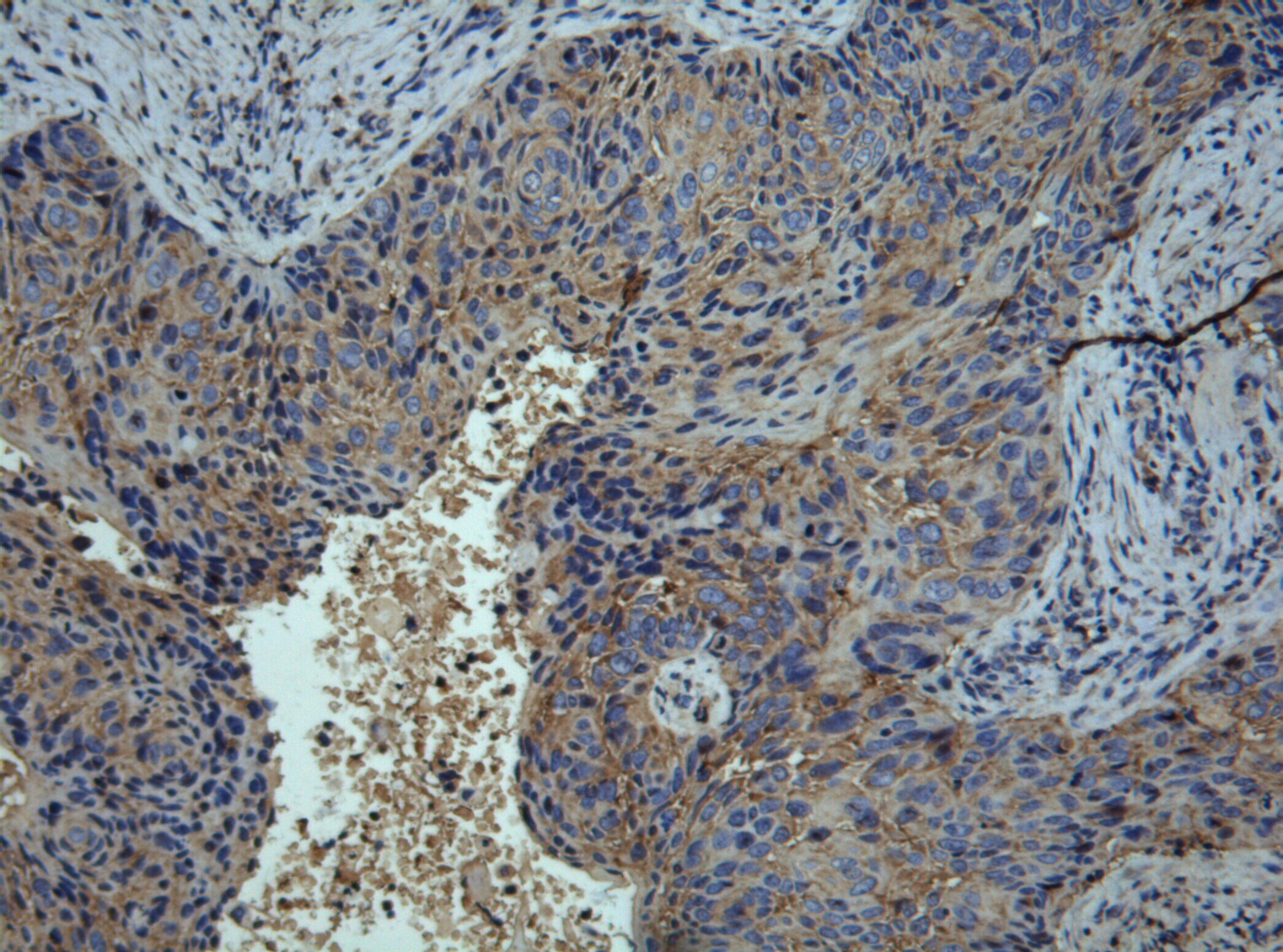

Supplement: S1 File — (ZIP) [file pone.0315242.s001.zip › IHC-TRPC1/23c++.jpg]

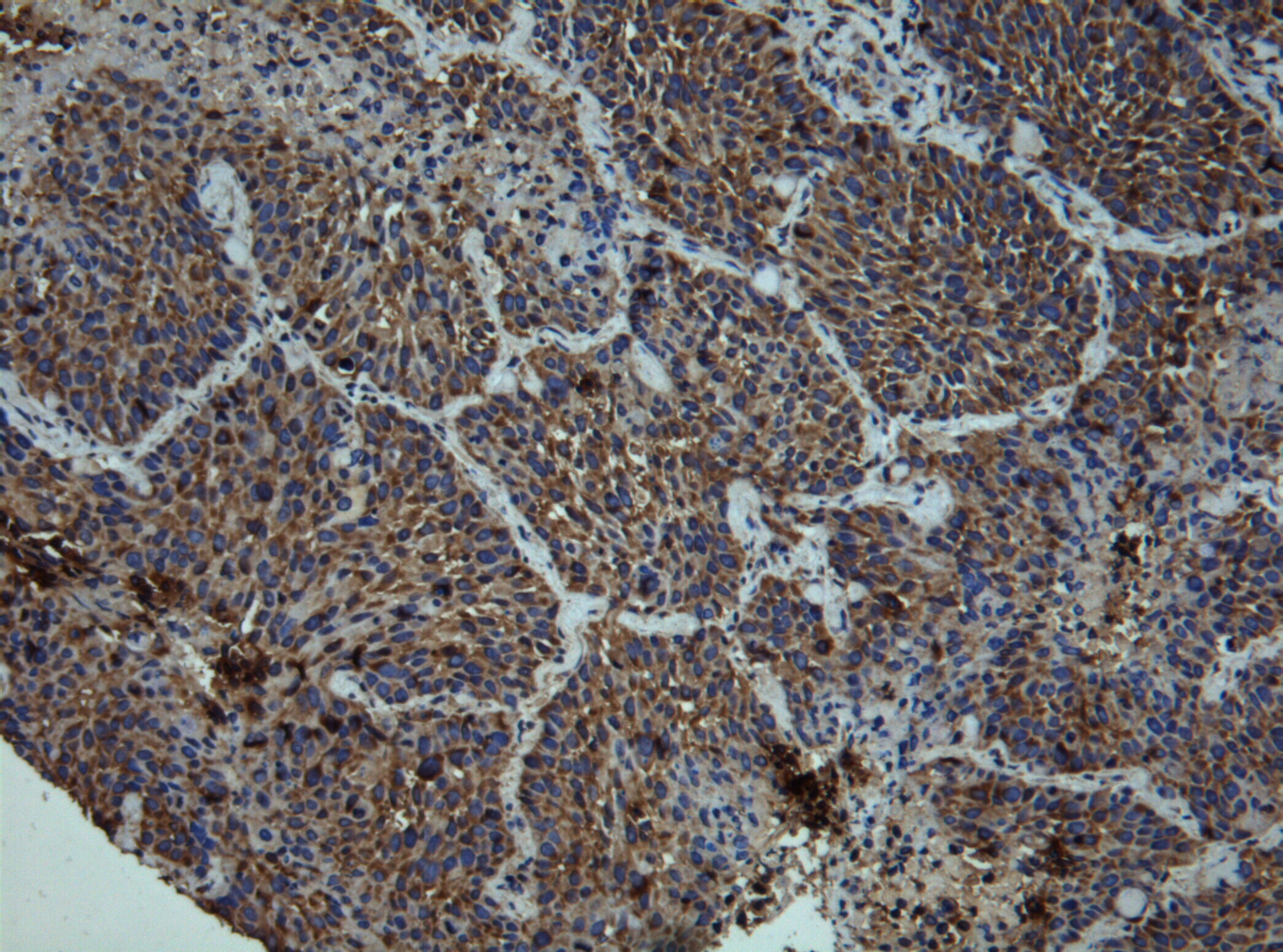

Supplement: S1 File — (ZIP) [file pone.0315242.s001.zip › IHC-TRPC1/24c++.jpg]

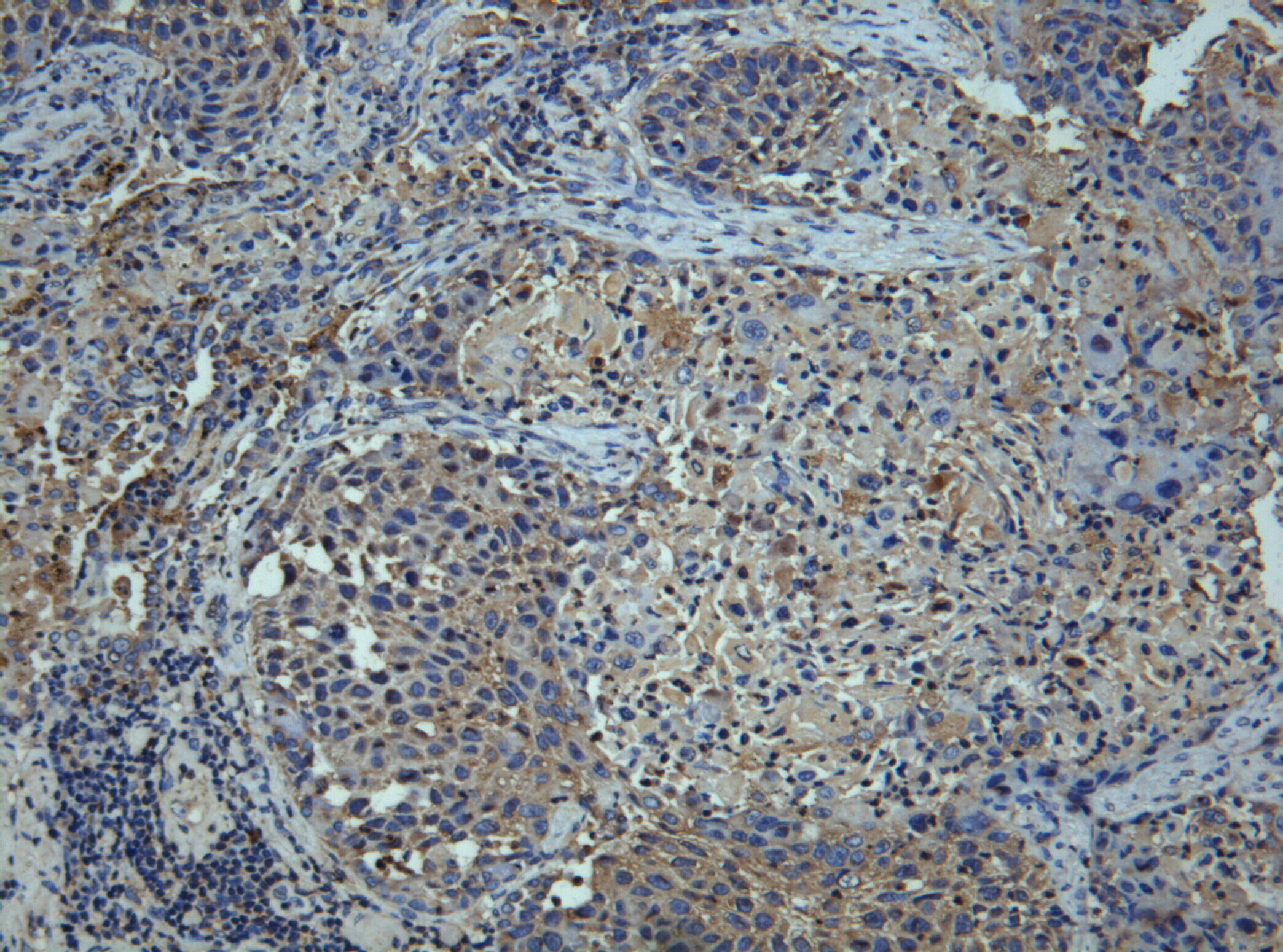

Supplement: S1 File — (ZIP) [file pone.0315242.s001.zip › IHC-TRPC1/25c++.jpg]

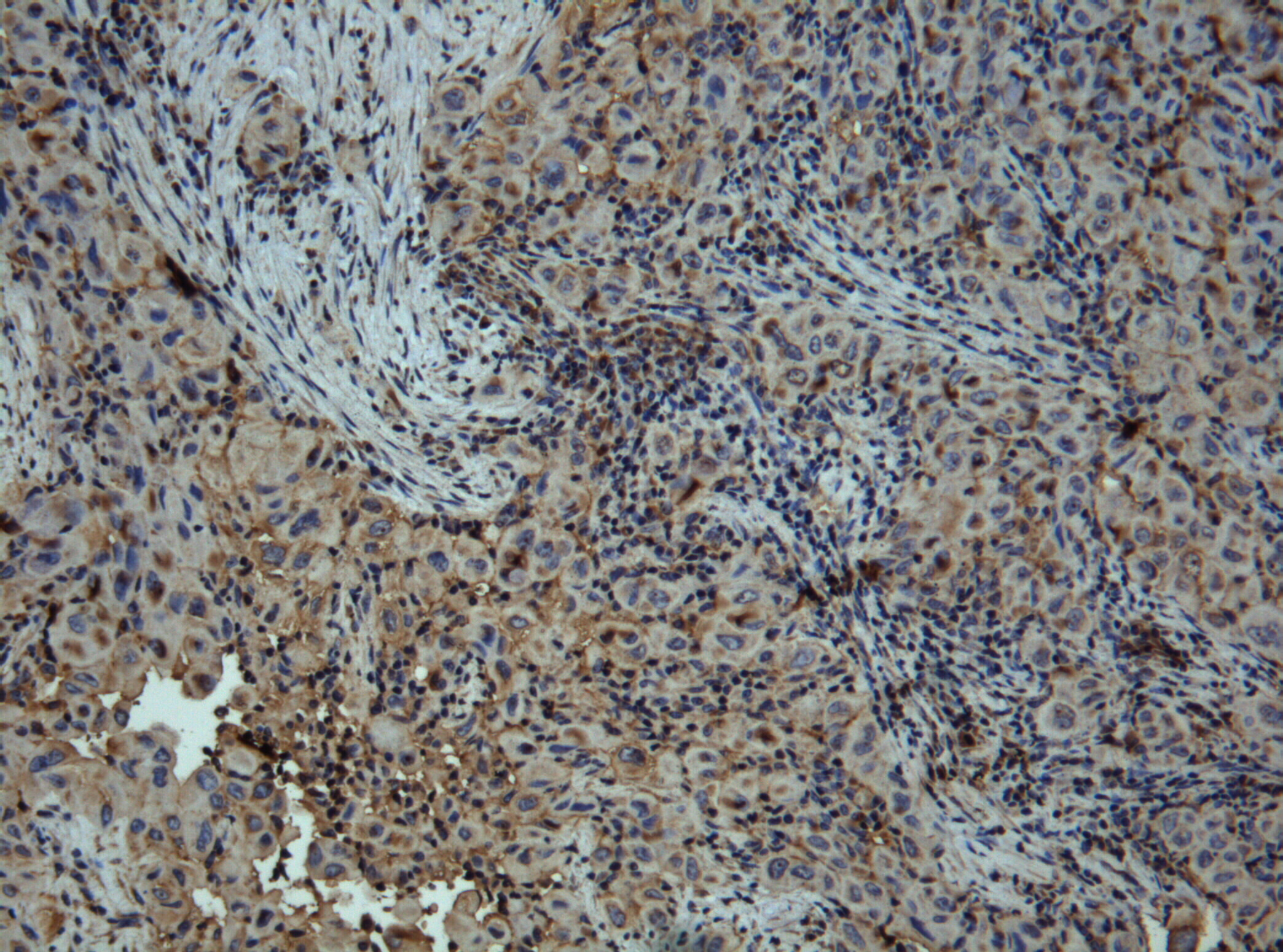

Supplement: S1 File — (ZIP) [file pone.0315242.s001.zip › IHC-TRPC1/27c++.jpg]

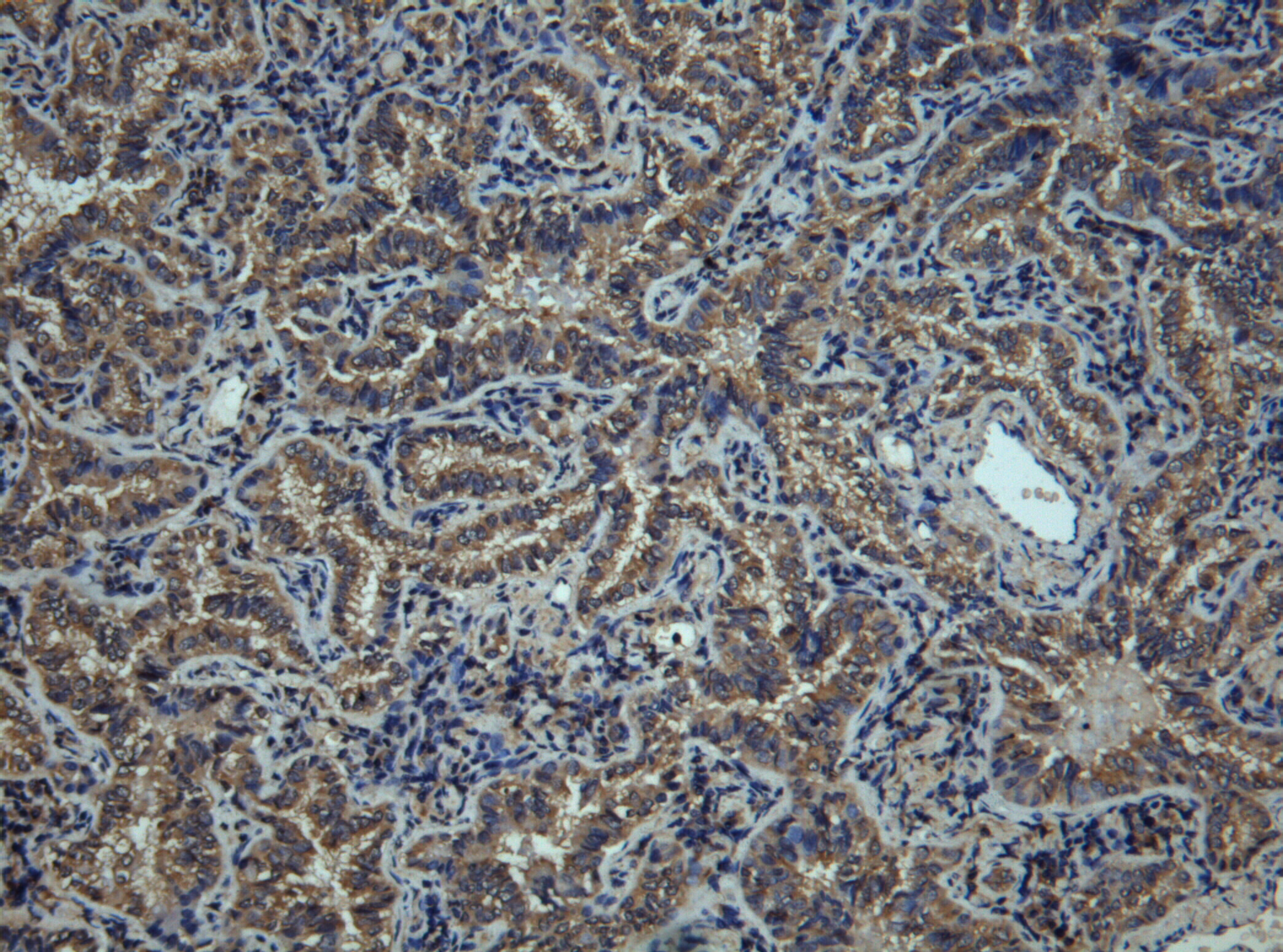

Supplement: S1 File — (ZIP) [file pone.0315242.s001.zip › IHC-TRPC1/28c ++~+++.jpg]

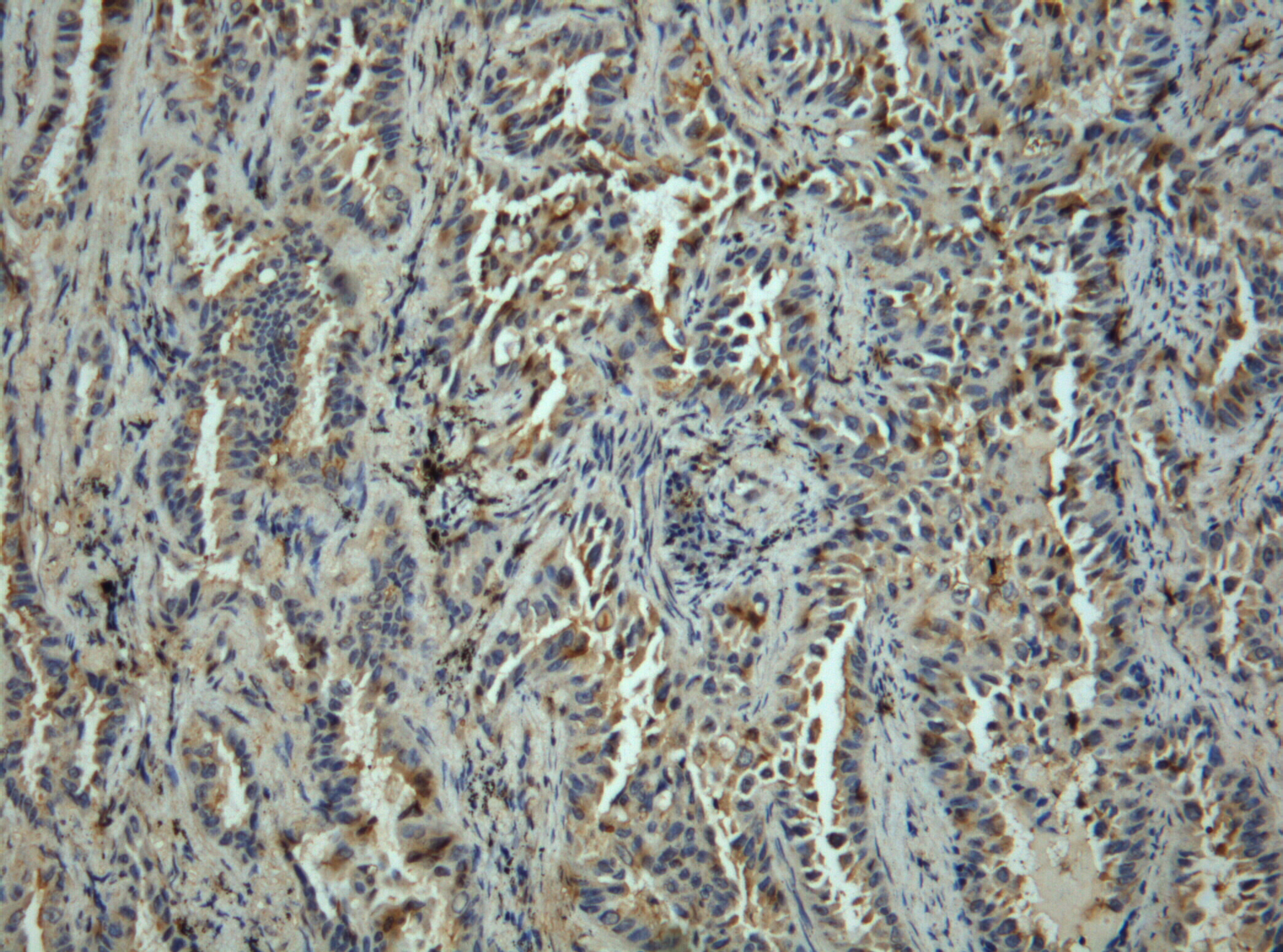

Supplement: S1 File — (ZIP) [file pone.0315242.s001.zip › IHC-TRPC3/11c++.jpg]

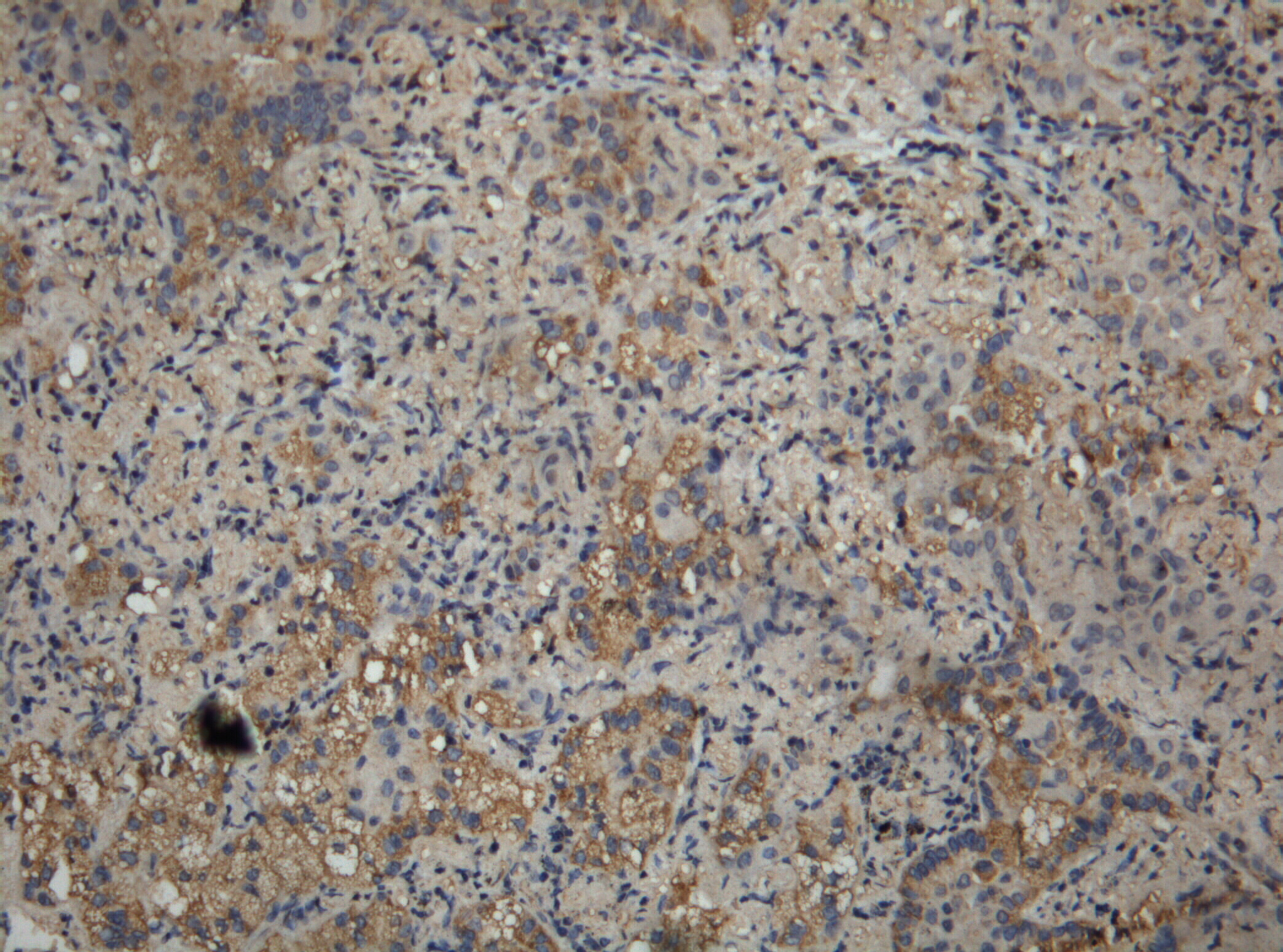

Supplement: S1 File — (ZIP) [file pone.0315242.s001.zip › IHC-TRPC3/12c++.jpg]

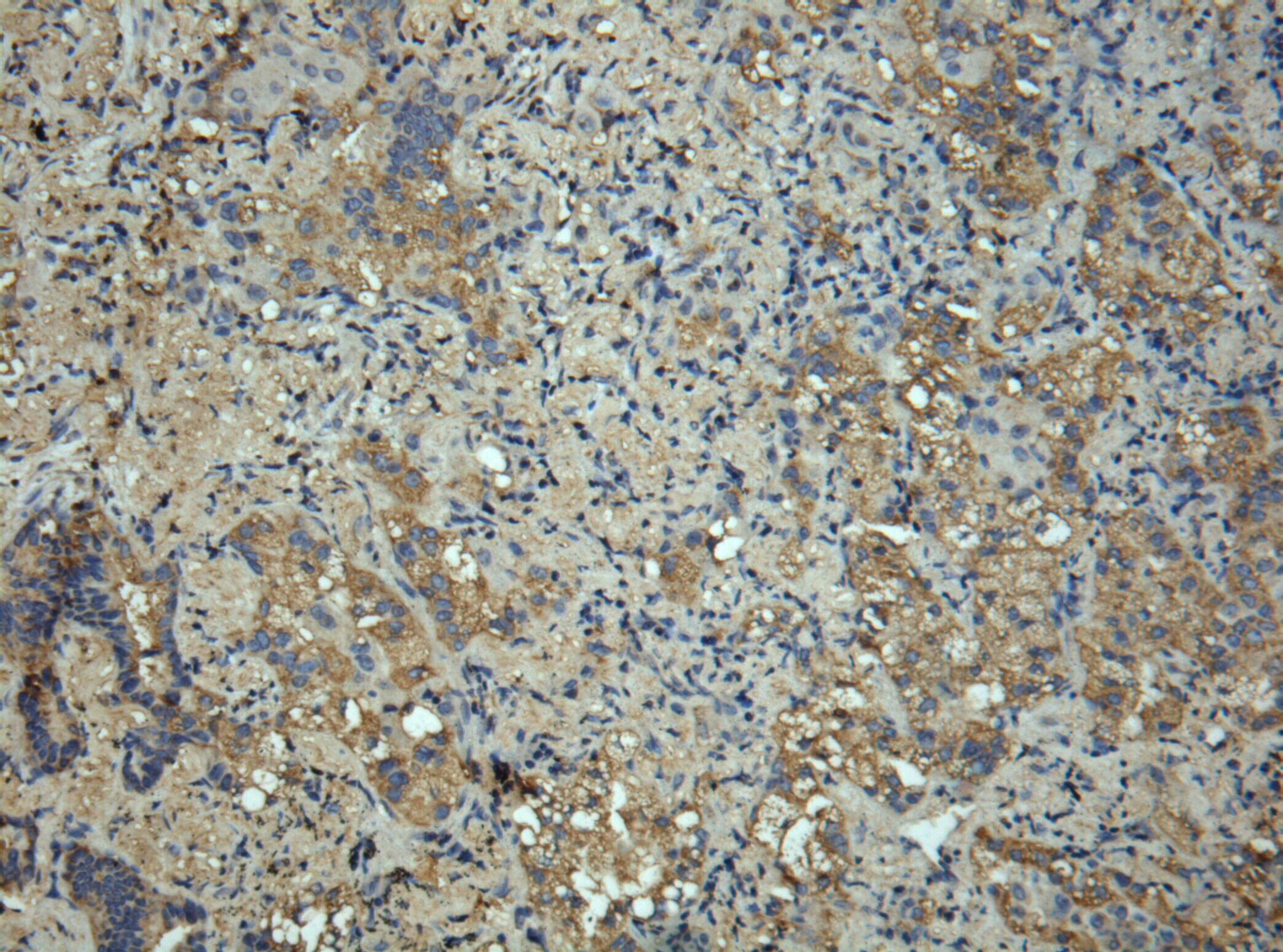

Supplement: S1 File — (ZIP) [file pone.0315242.s001.zip › IHC-TRPC3/14c++.jpg]

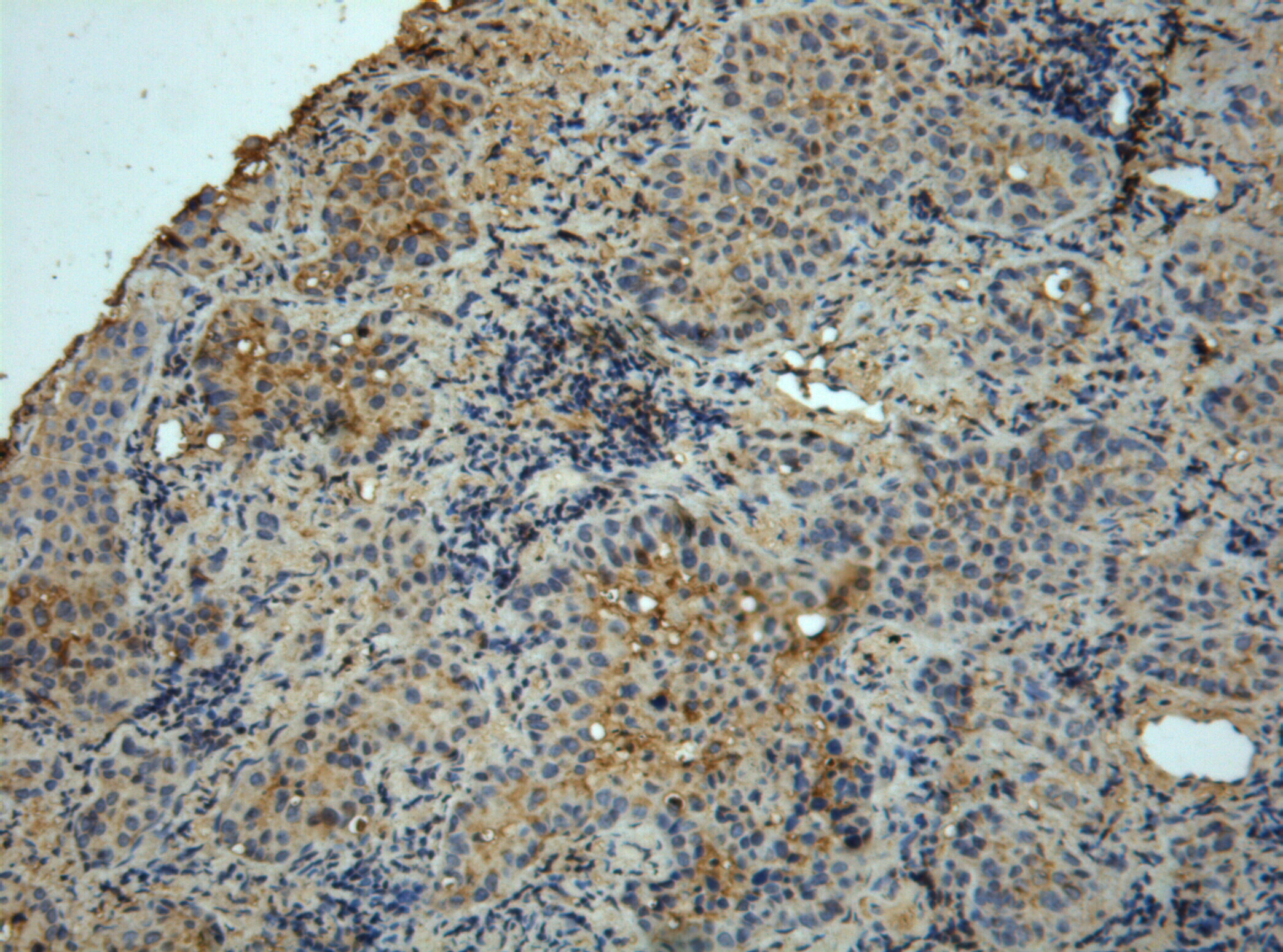

Supplement: S1 File — (ZIP) [file pone.0315242.s001.zip › IHC-TRPC3/15c++.jpg]

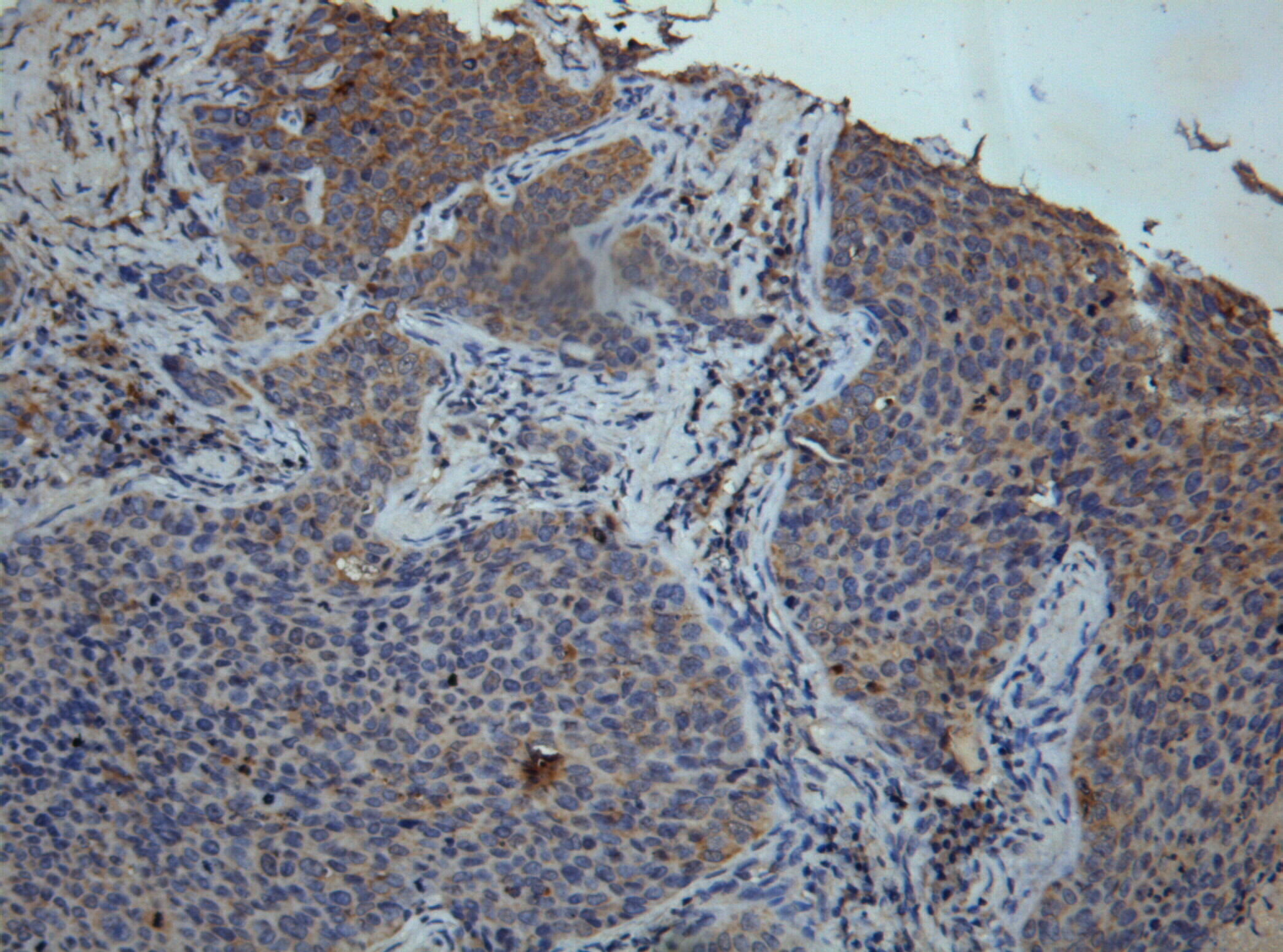

Supplement: S1 File — (ZIP) [file pone.0315242.s001.zip › IHC-TRPC3/16c+.jpg]

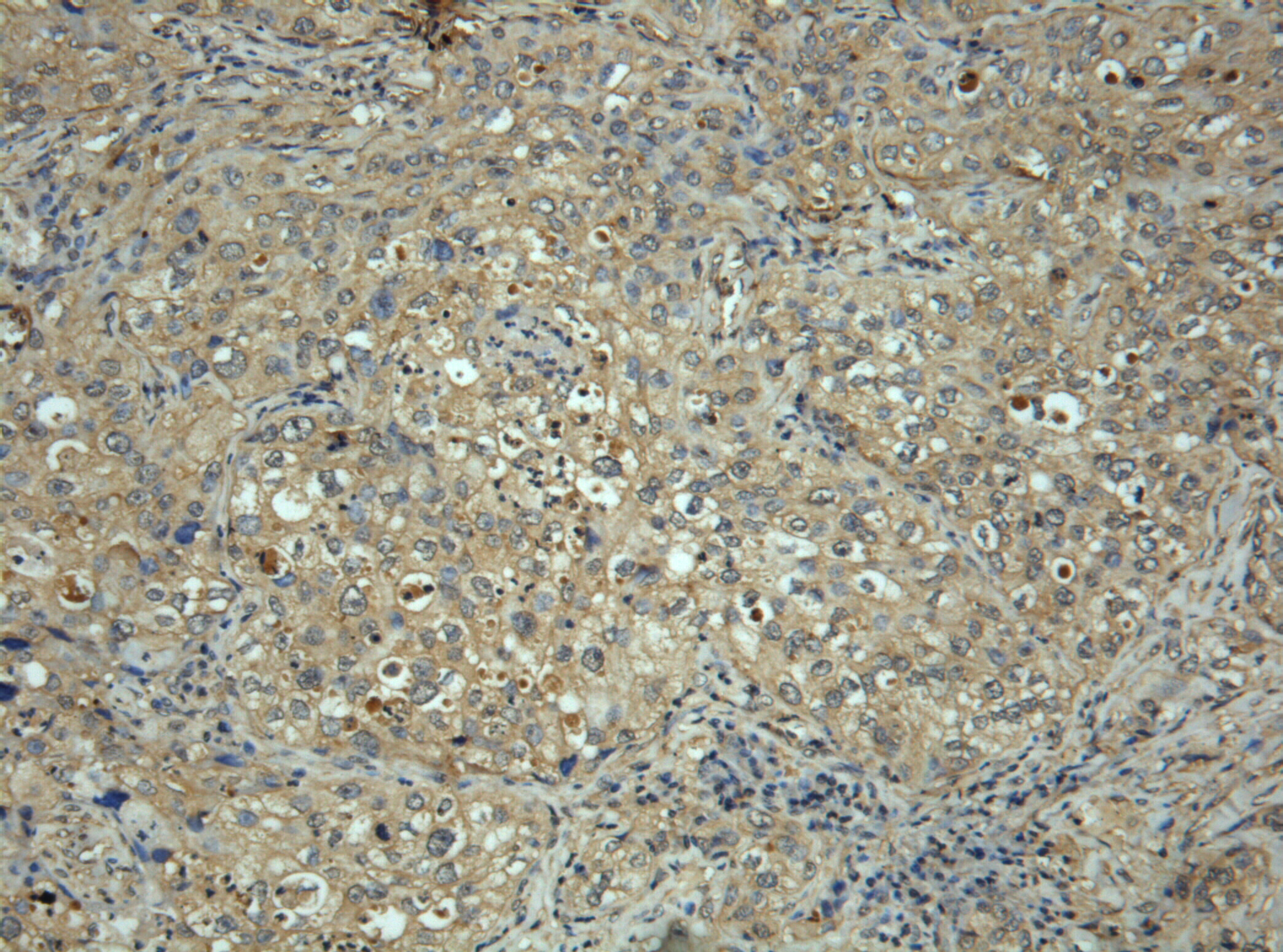

Supplement: S1 File — (ZIP) [file pone.0315242.s001.zip › IHC-TRPC3/17c++.jpg]

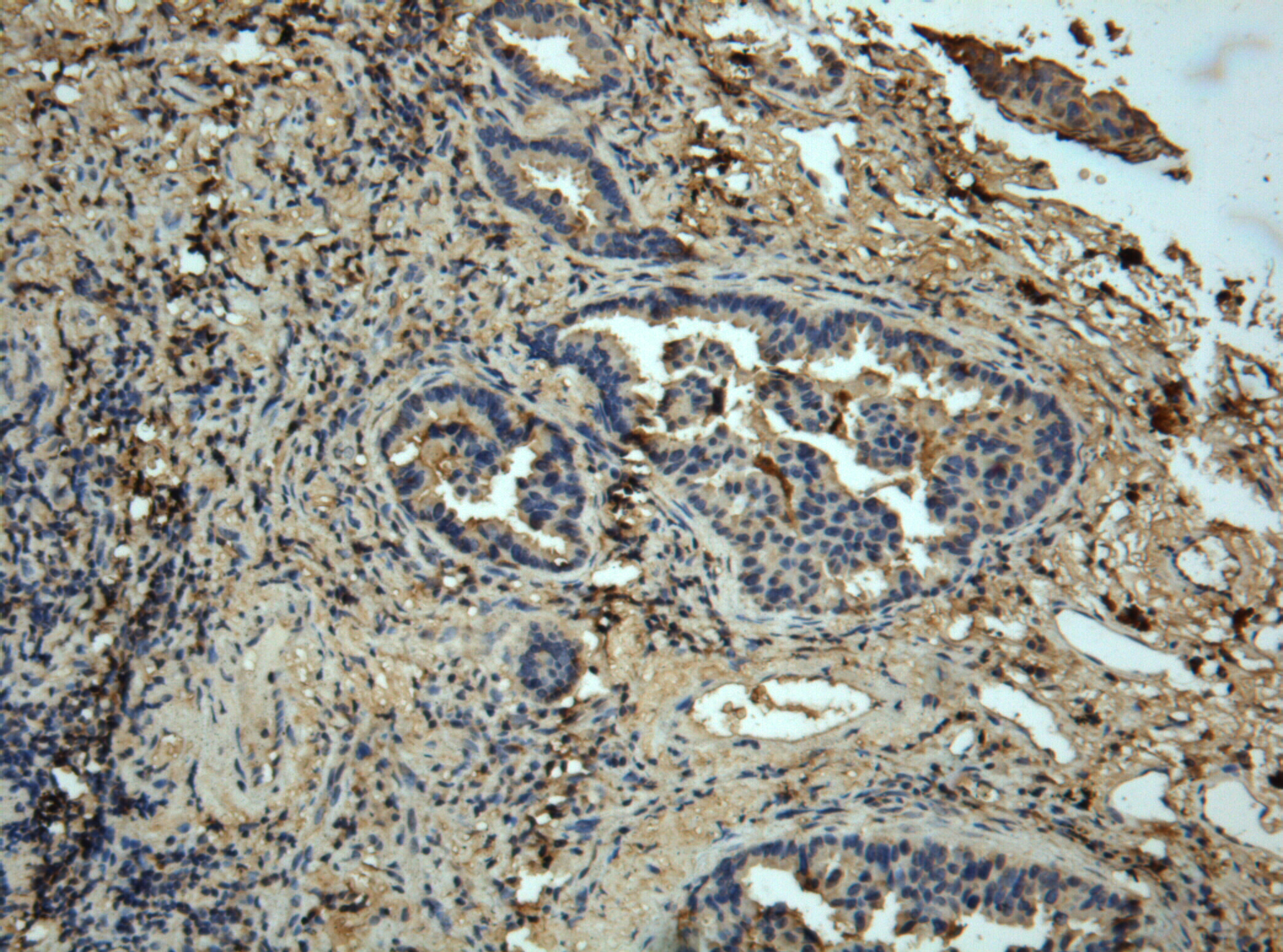

Supplement: S1 File — (ZIP) [file pone.0315242.s001.zip › IHC-TRPC3/18c+.jpg]

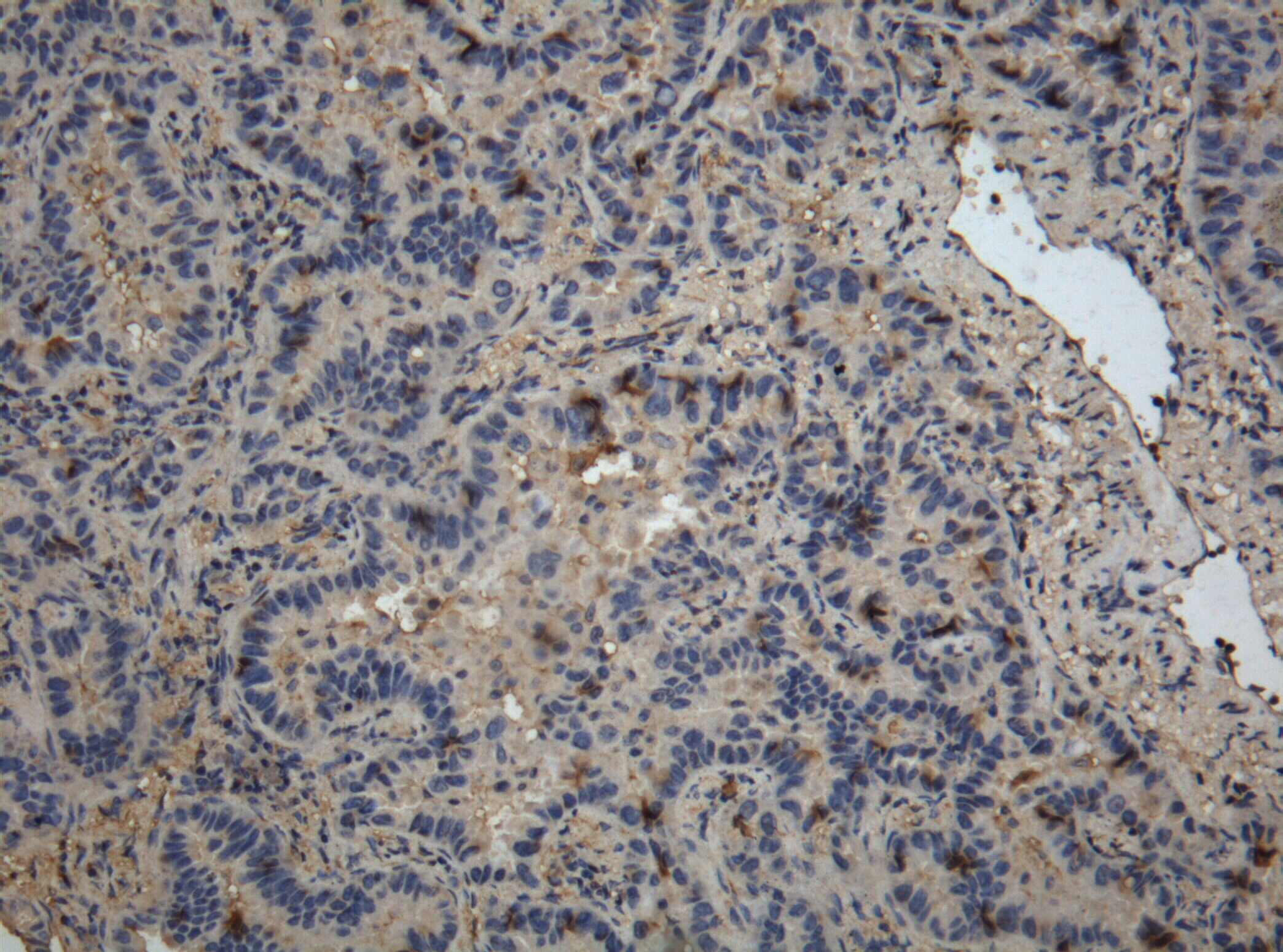

Supplement: S1 File — (ZIP) [file pone.0315242.s001.zip › IHC-TRPC3/1c+.jpg]

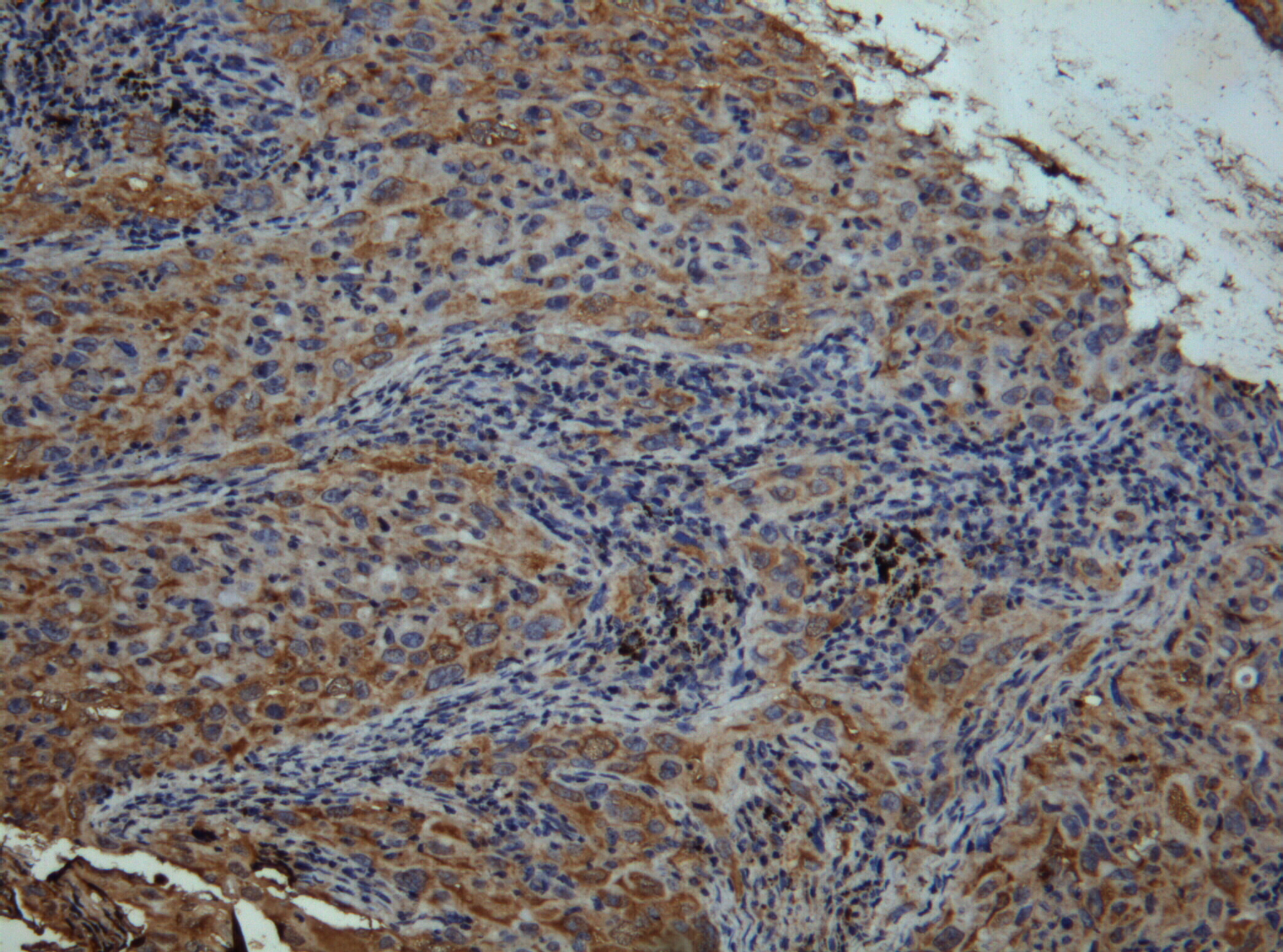

Supplement: S1 File — (ZIP) [file pone.0315242.s001.zip › IHC-TRPC3/20c++.jpg]

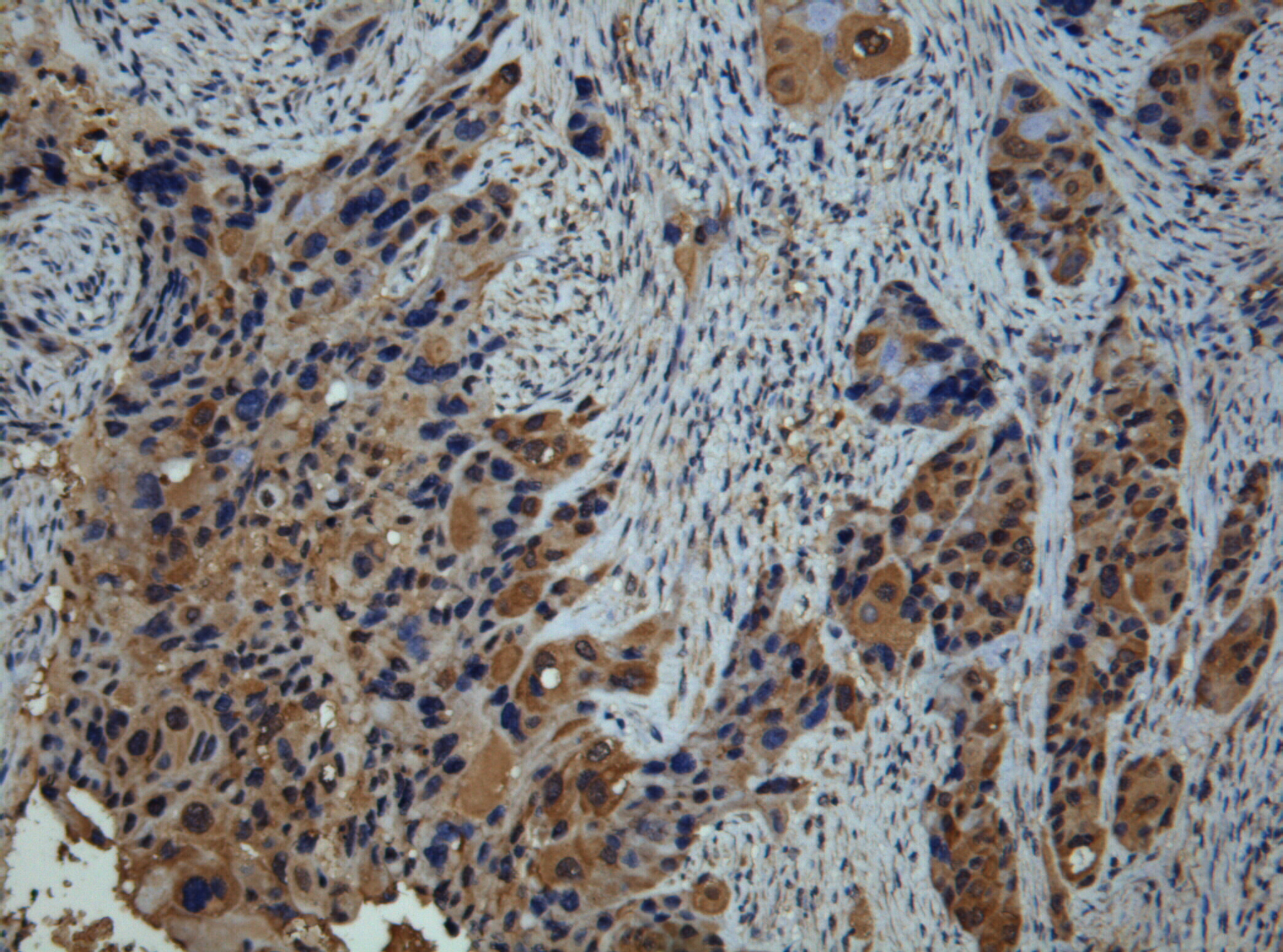

Supplement: S1 File — (ZIP) [file pone.0315242.s001.zip › IHC-TRPC3/22c++.jpg]

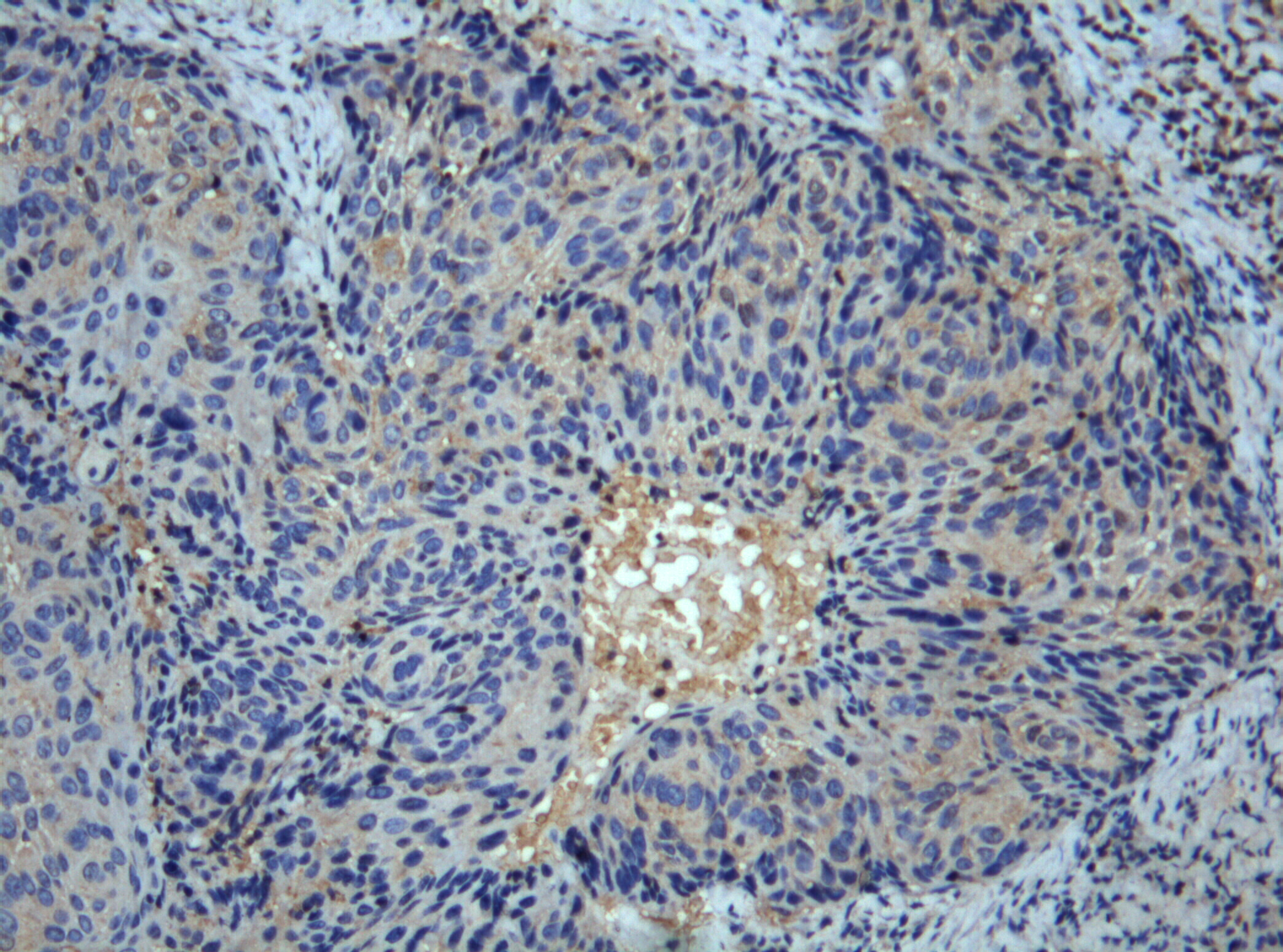

Supplement: S1 File — (ZIP) [file pone.0315242.s001.zip › IHC-TRPC3/23c+.jpg]

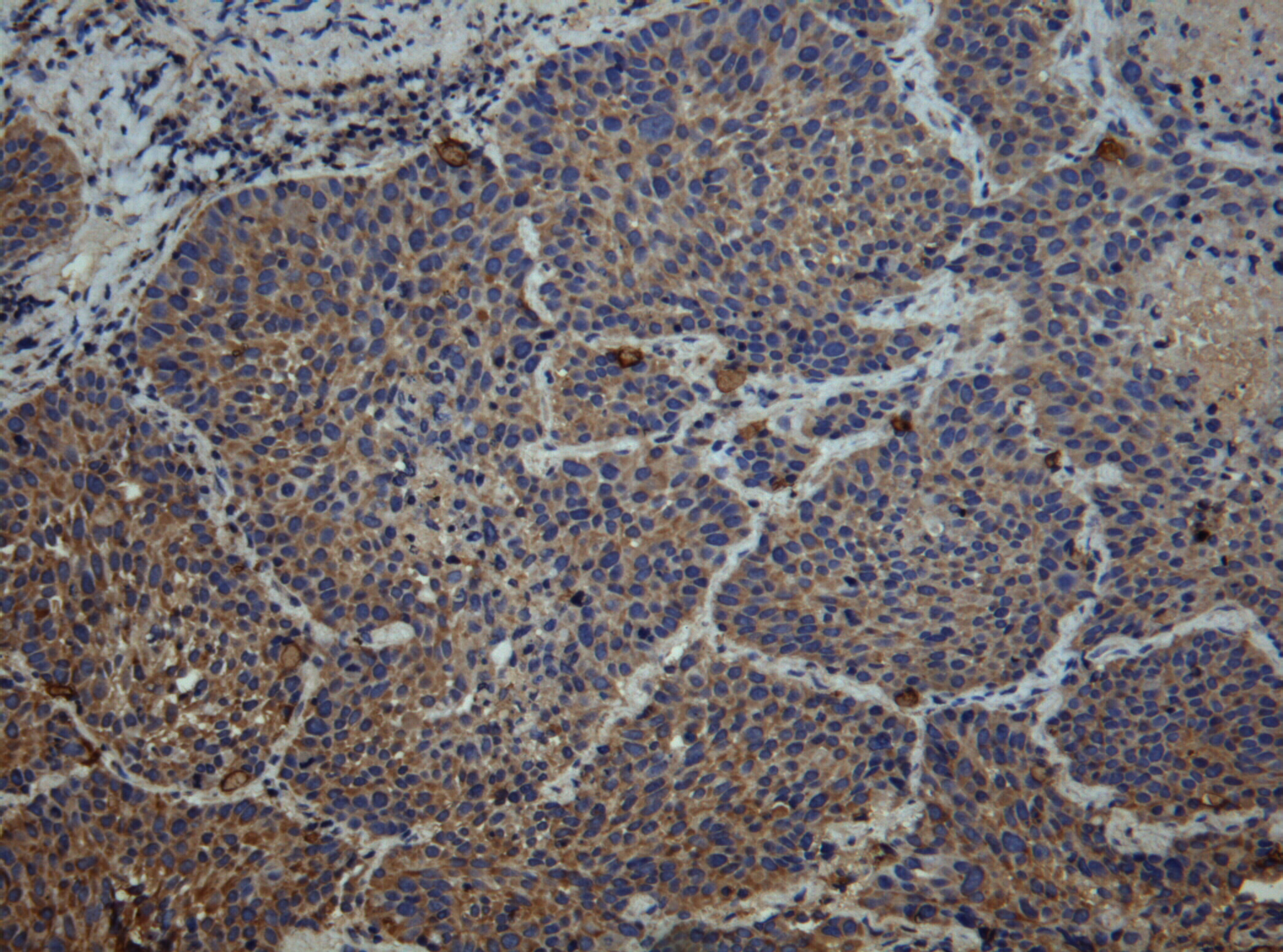

Supplement: S1 File — (ZIP) [file pone.0315242.s001.zip › IHC-TRPC3/24c++.jpg]

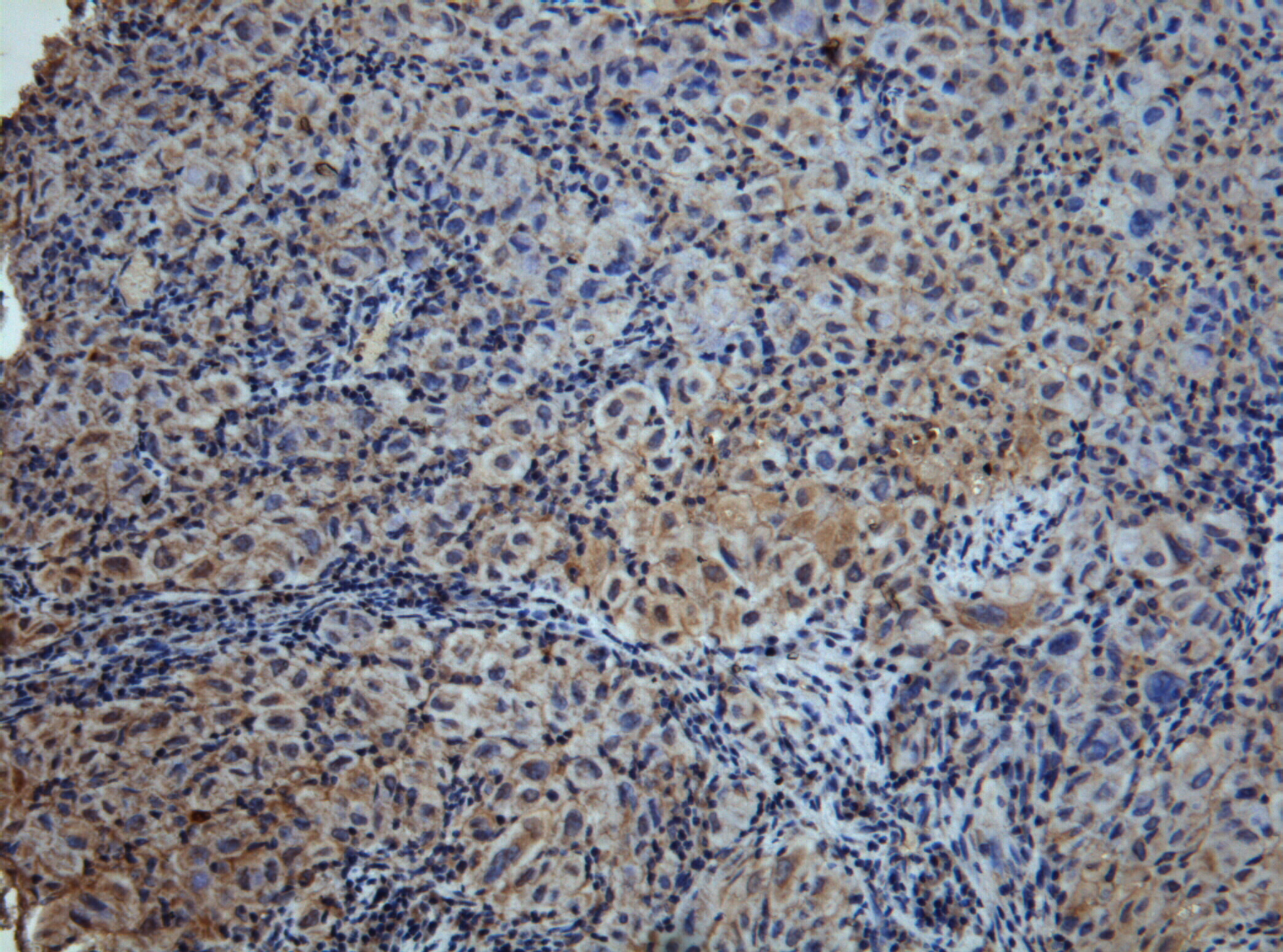

Supplement: S1 File — (ZIP) [file pone.0315242.s001.zip › IHC-TRPC3/27c++.jpg]

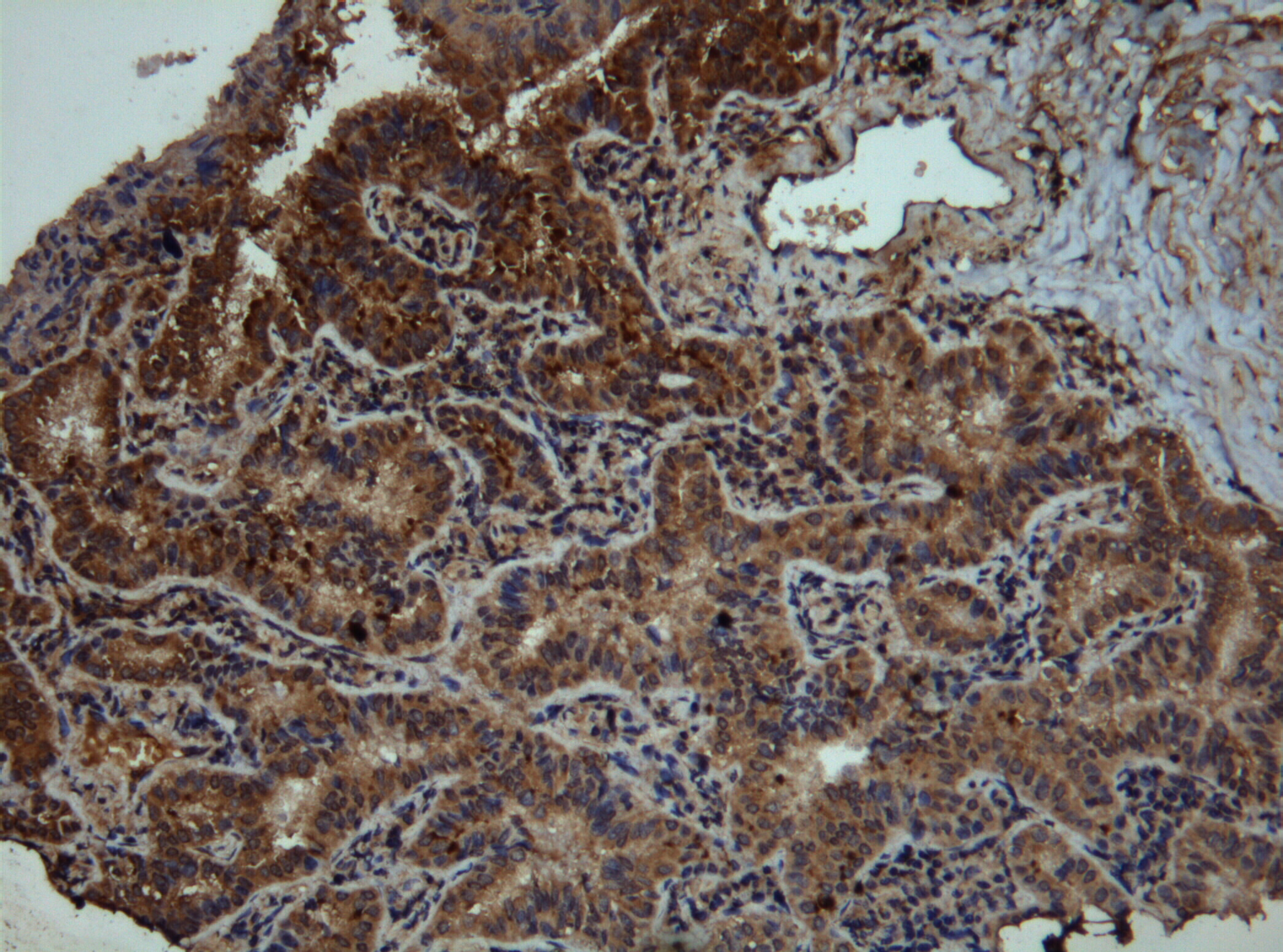

Supplement: S1 File — (ZIP) [file pone.0315242.s001.zip › IHC-TRPC3/28C++.jpg]

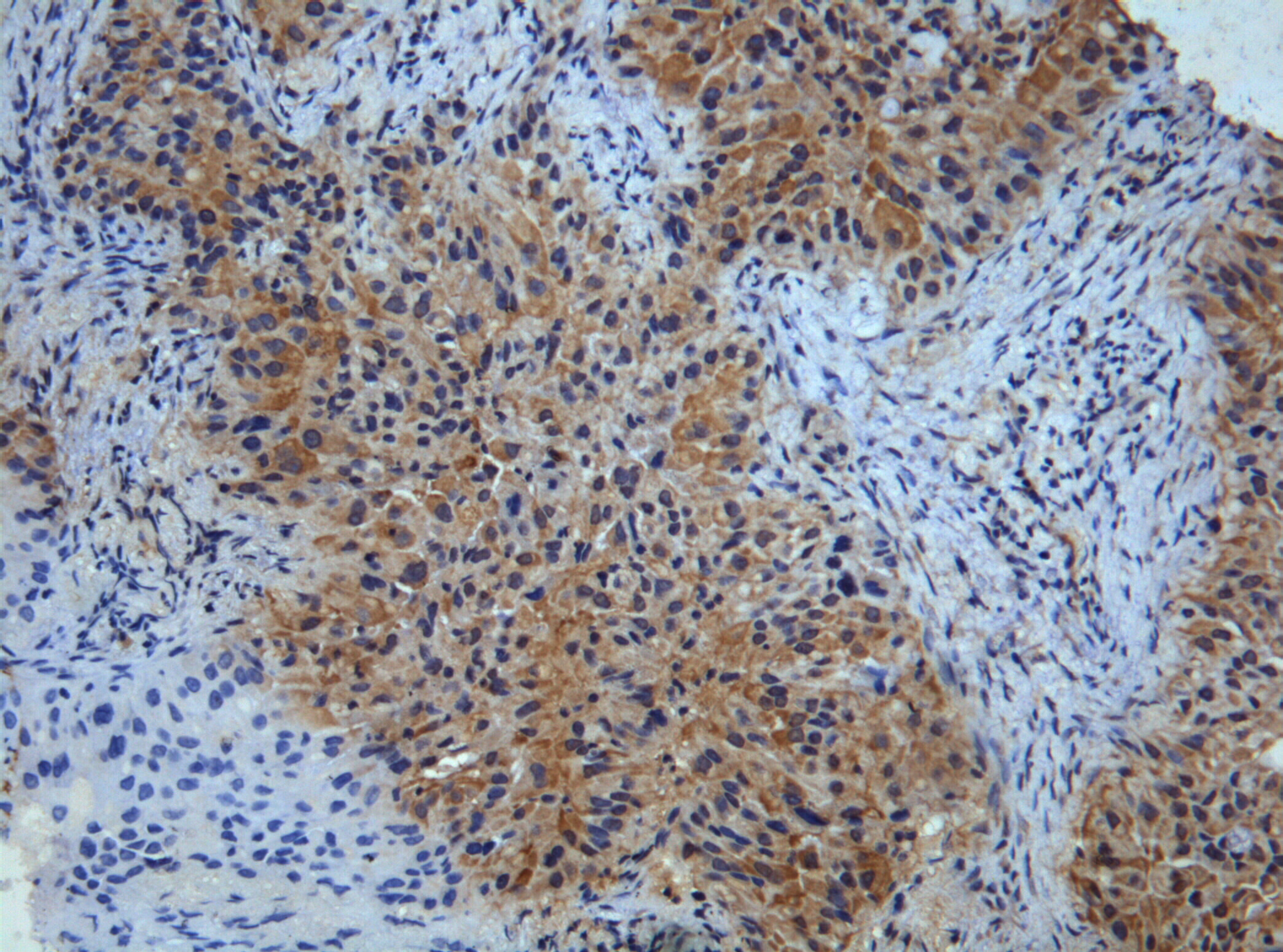

Supplement: S1 File — (ZIP) [file pone.0315242.s001.zip › IHC-TRPC3/29c++.jpg]

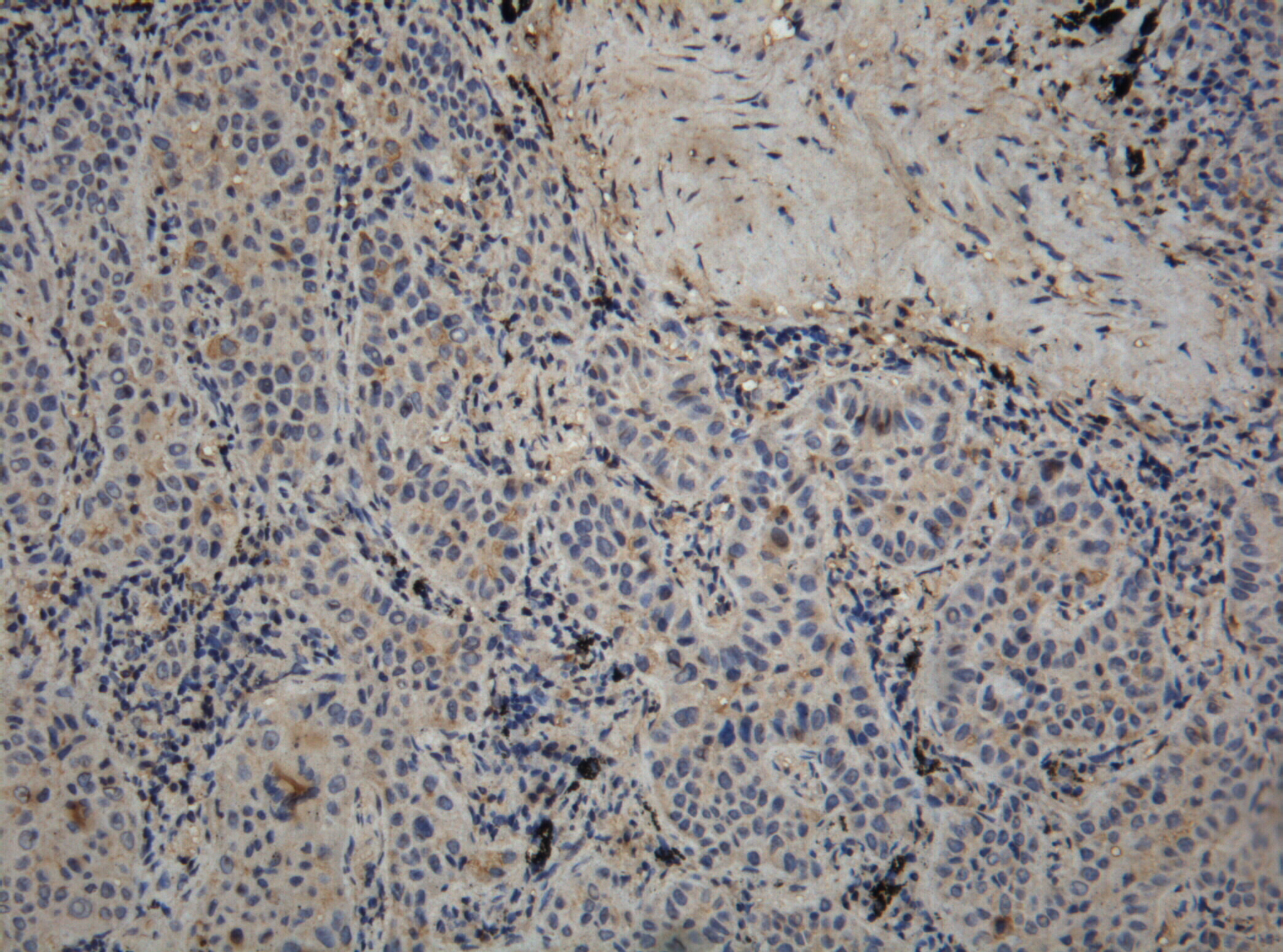

Supplement: S1 File — (ZIP) [file pone.0315242.s001.zip › IHC-TRPC3/3c+.jpg]

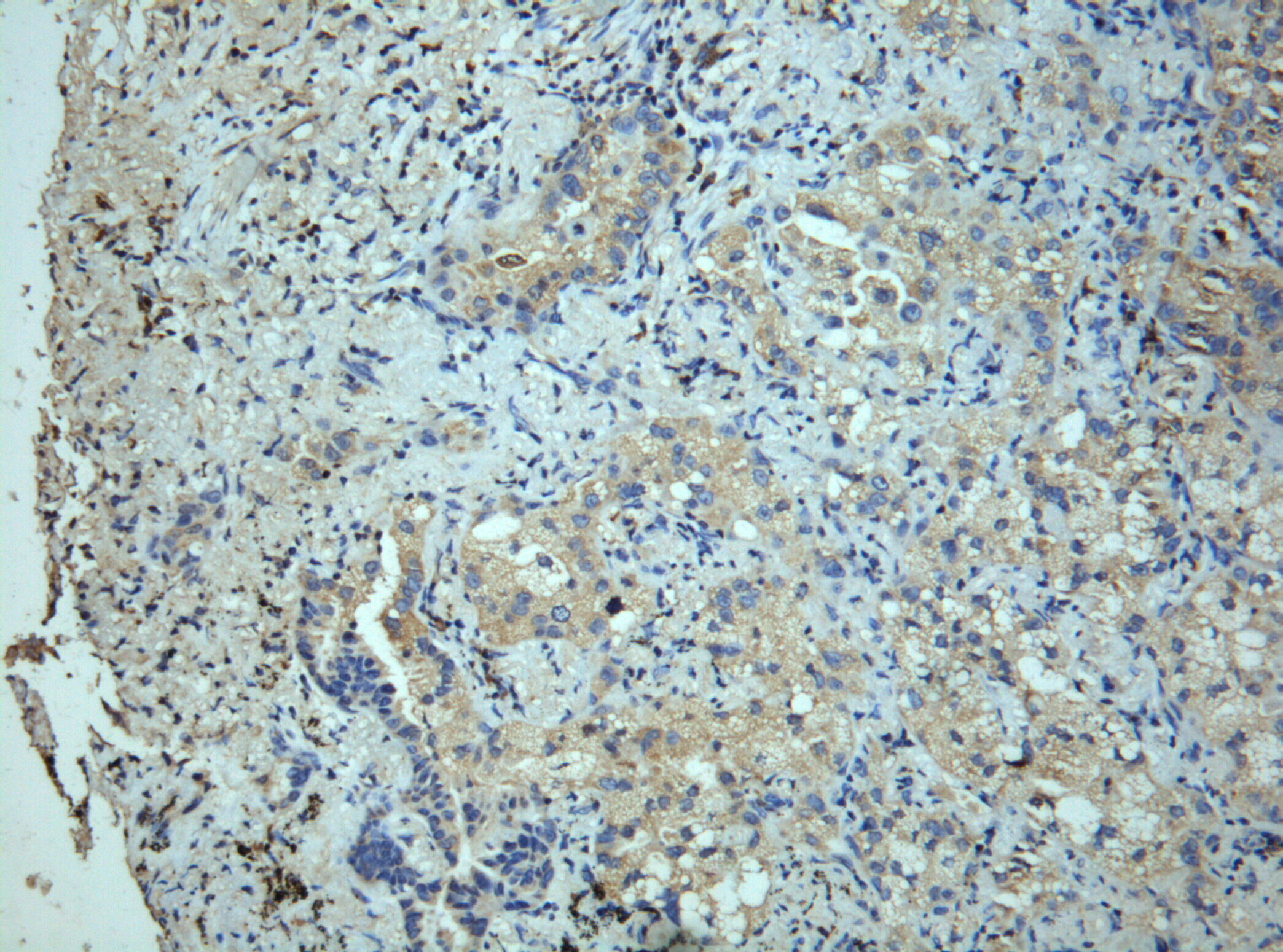

Supplement: S1 File — (ZIP) [file pone.0315242.s001.zip › IHC-TRPC6/12c+.jpg]

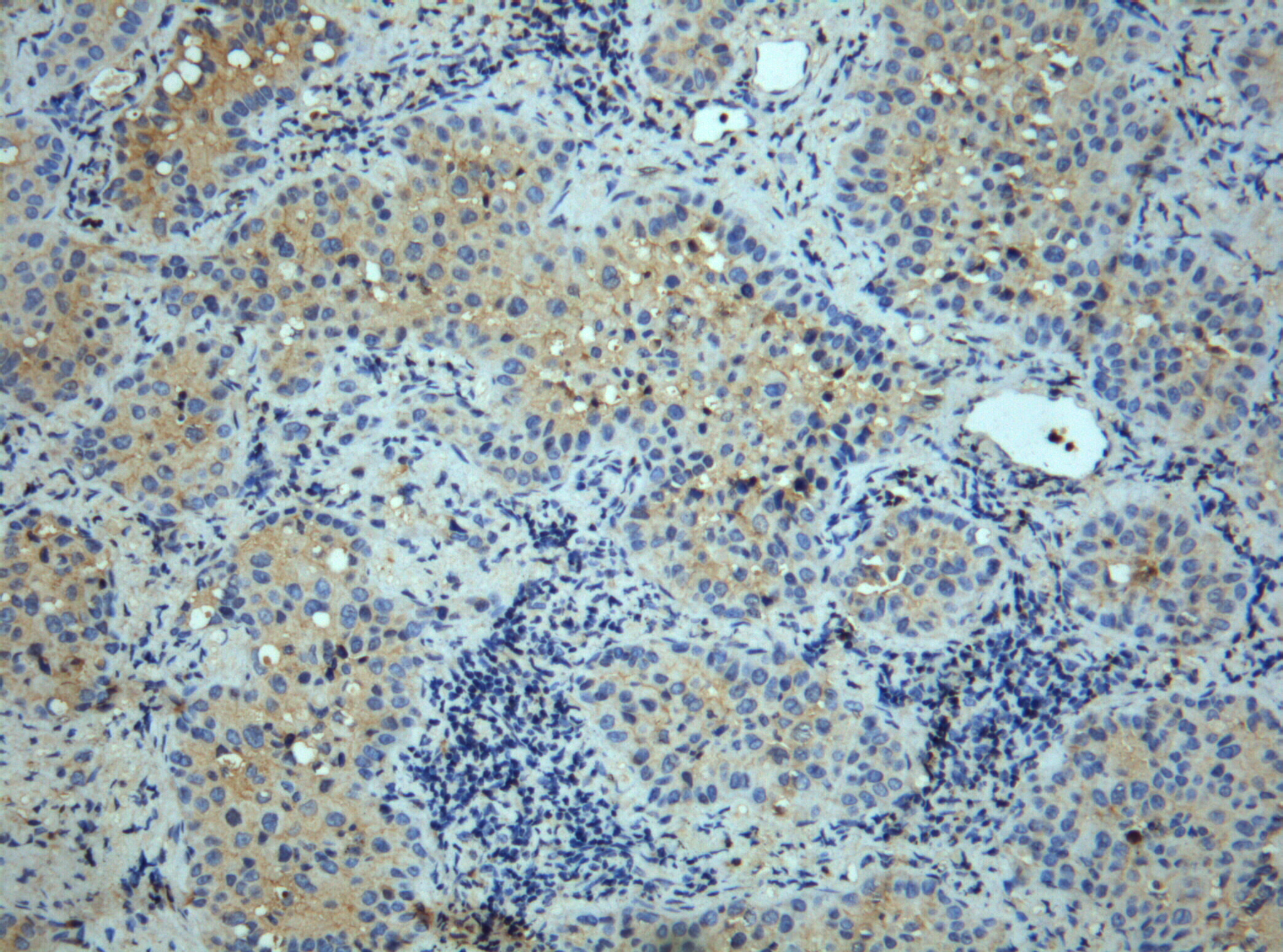

Supplement: S1 File — (ZIP) [file pone.0315242.s001.zip › IHC-TRPC6/13c+.jpg]

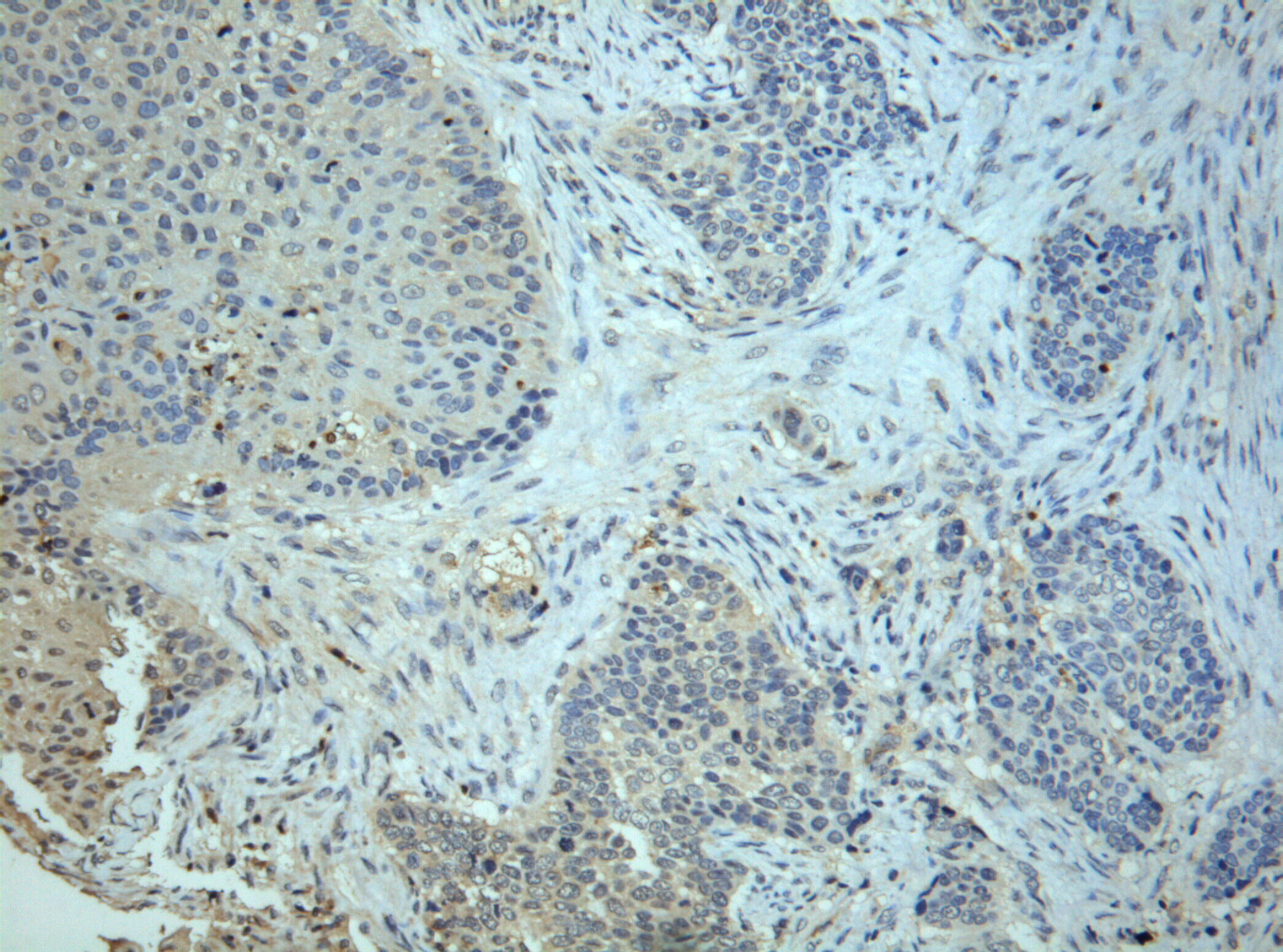

Supplement: S1 File — (ZIP) [file pone.0315242.s001.zip › IHC-TRPC6/15C++.jpg]

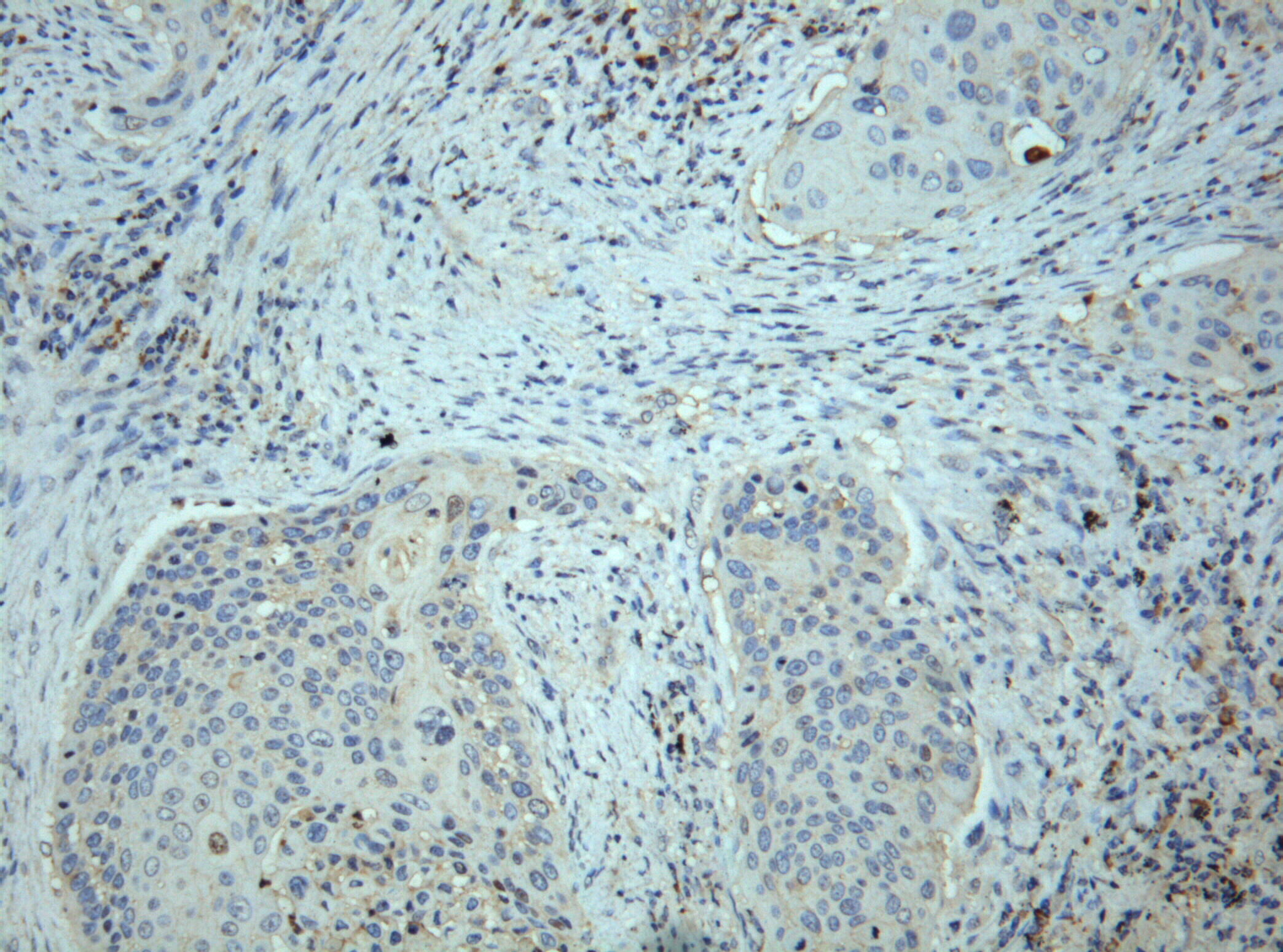

Supplement: S1 File — (ZIP) [file pone.0315242.s001.zip › IHC-TRPC6/16c+.jpg]

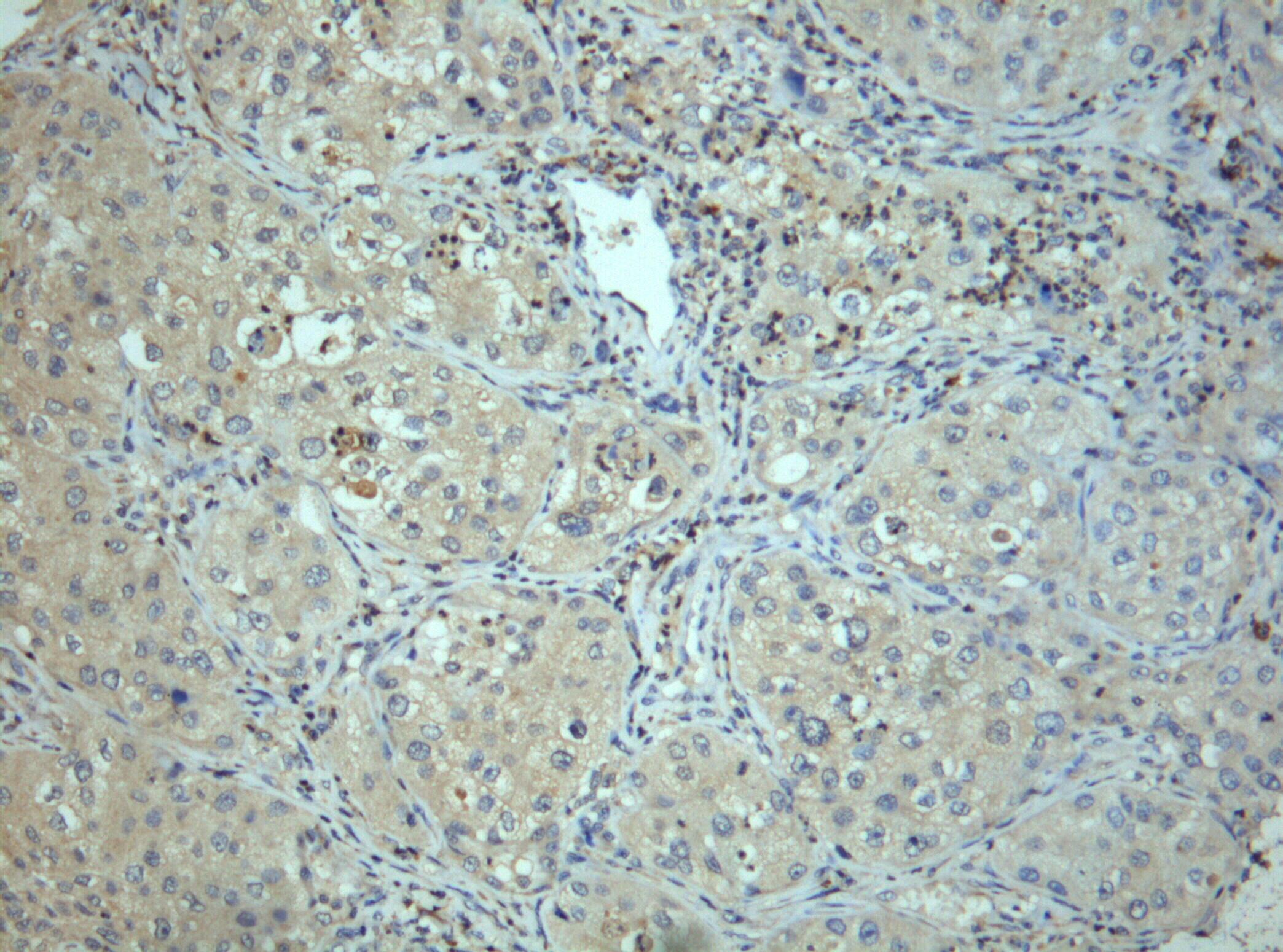

Supplement: S1 File — (ZIP) [file pone.0315242.s001.zip › IHC-TRPC6/17c++.jpg]

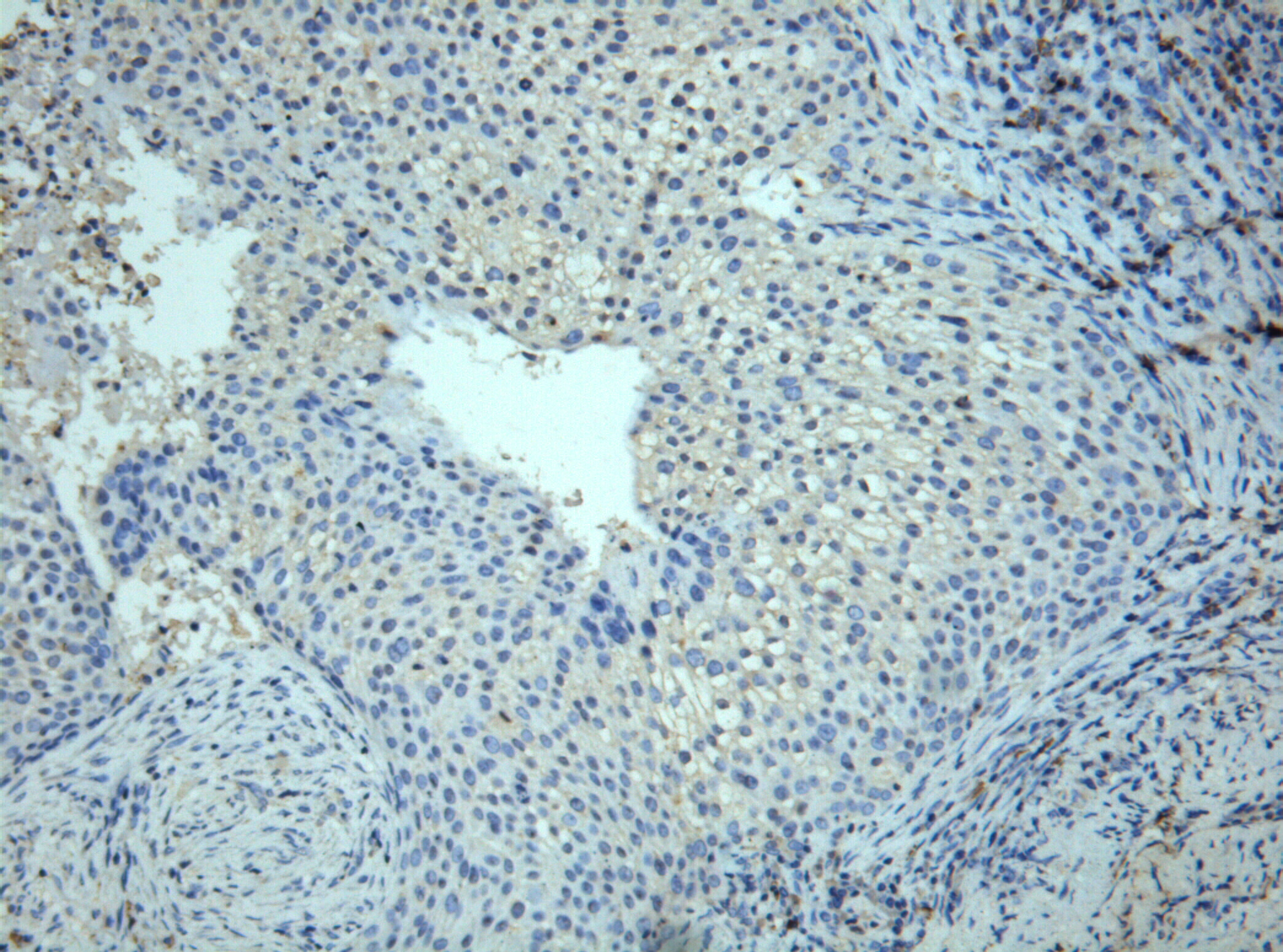

Supplement: S1 File — (ZIP) [file pone.0315242.s001.zip › IHC-TRPC6/18c+.jpg]

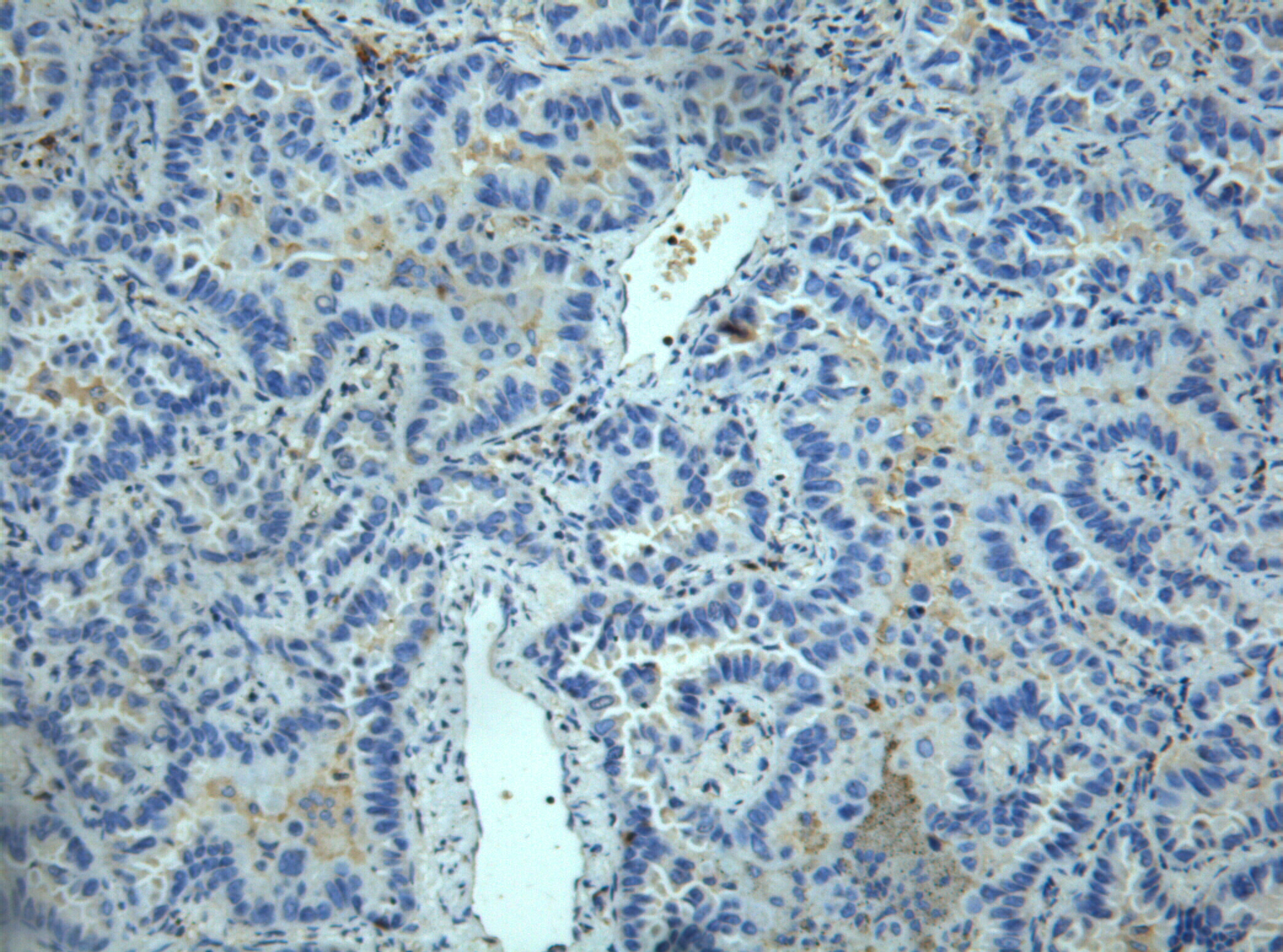

Supplement: S1 File — (ZIP) [file pone.0315242.s001.zip › IHC-TRPC6/1c-.jpg]

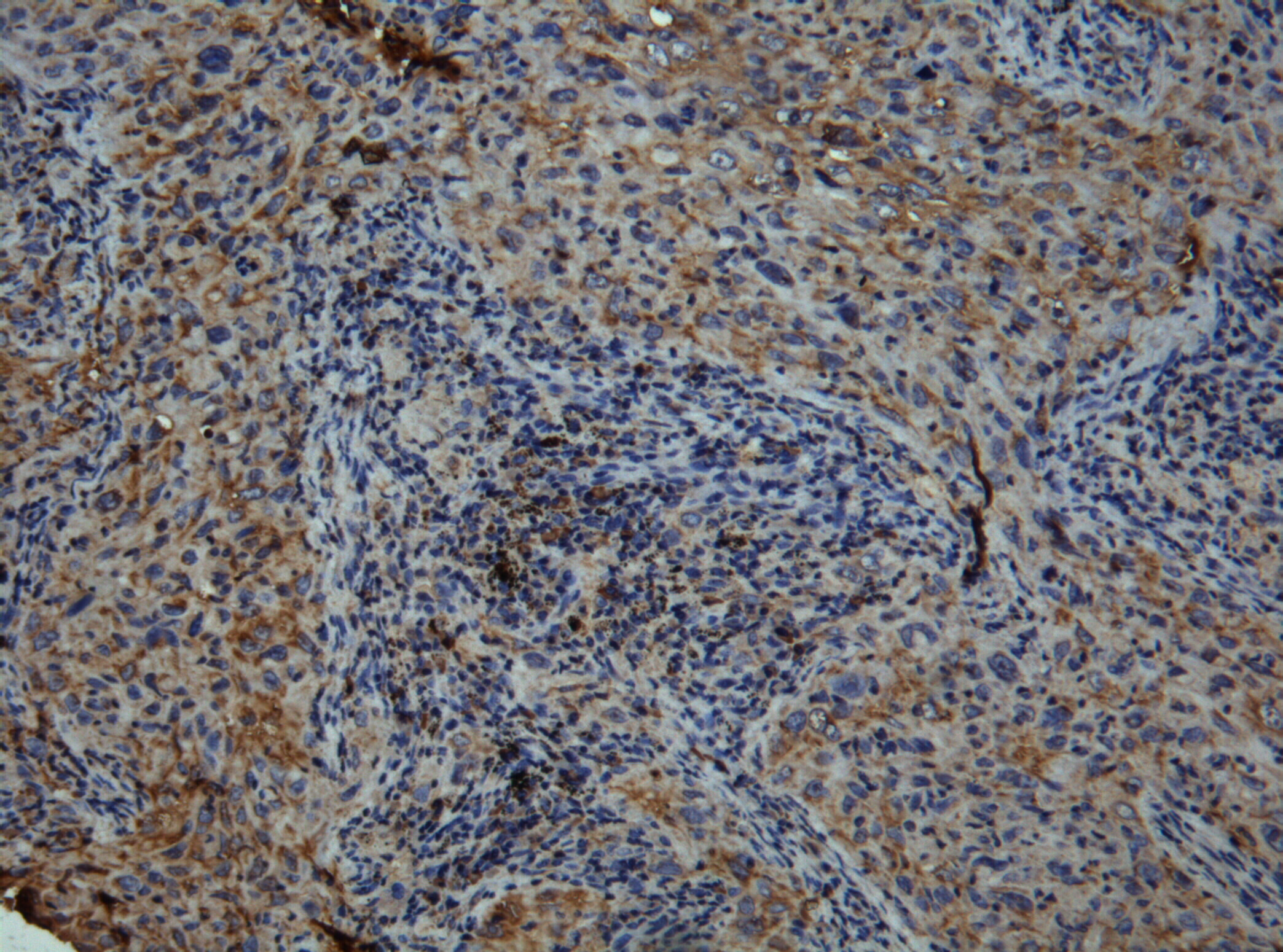

Supplement: S1 File — (ZIP) [file pone.0315242.s001.zip › IHC-TRPC6/20C++.jpg]

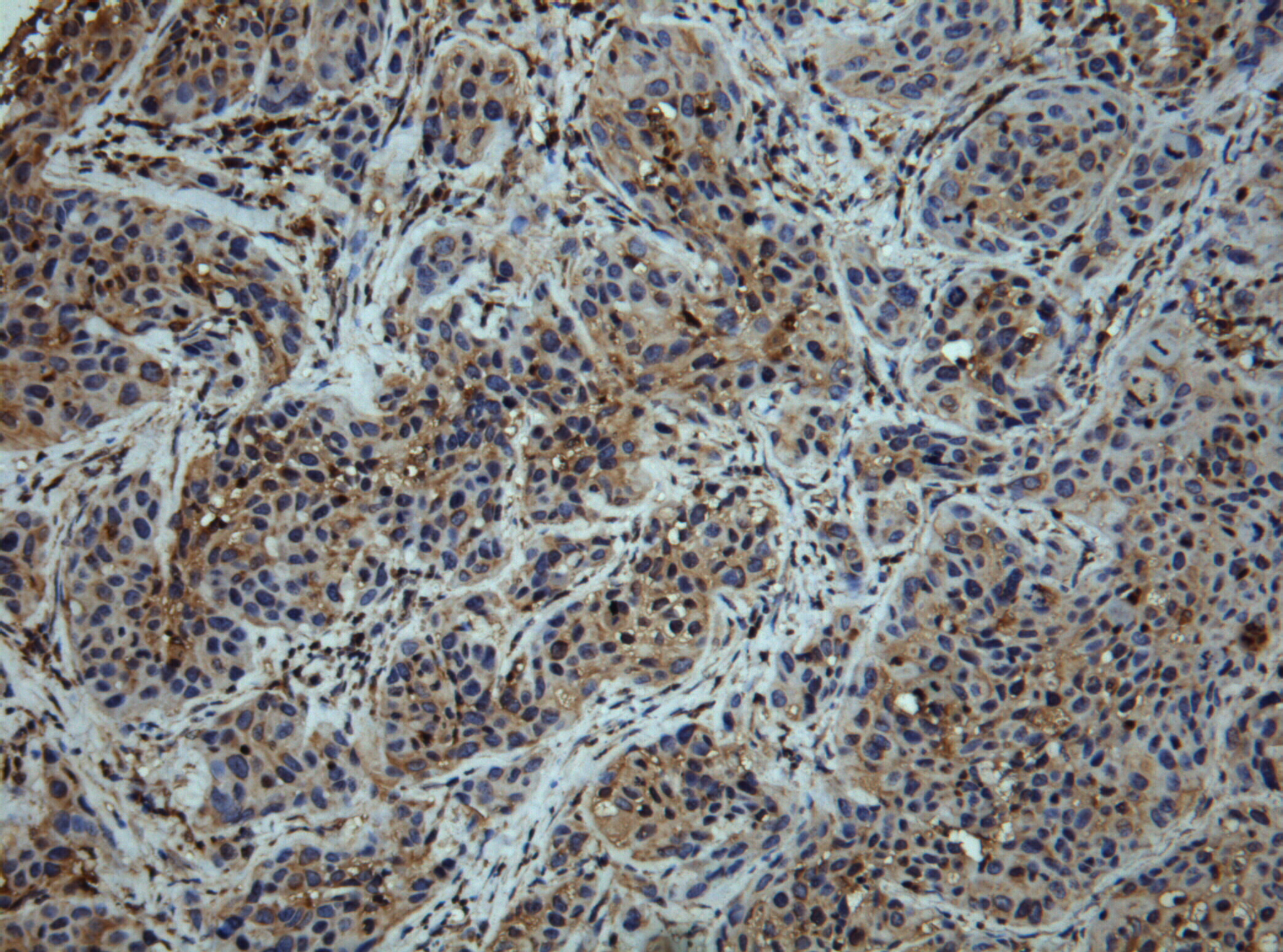

Supplement: S1 File — (ZIP) [file pone.0315242.s001.zip › IHC-TRPC6/21c++.jpg]

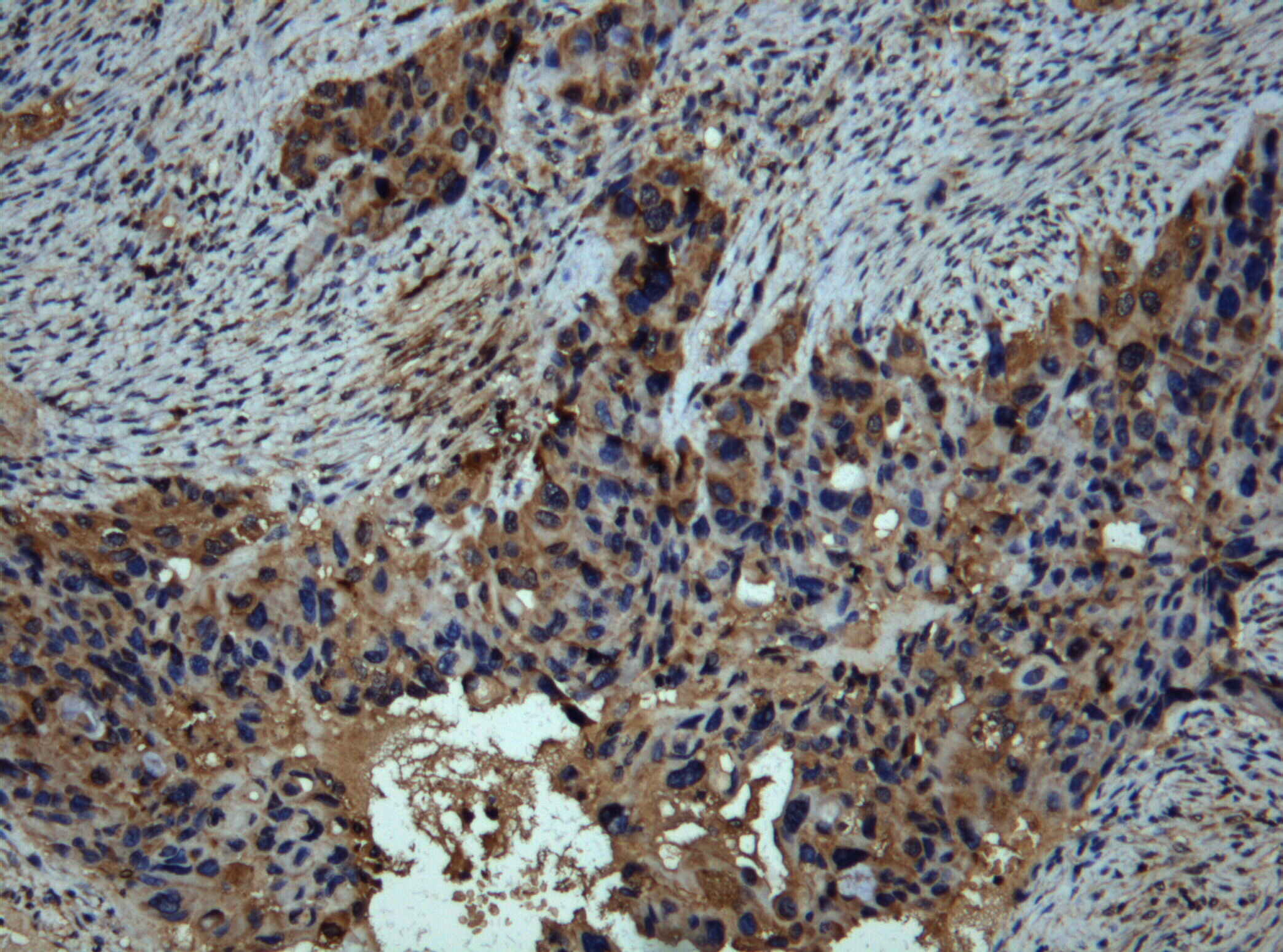

Supplement: S1 File — (ZIP) [file pone.0315242.s001.zip › IHC-TRPC6/22c++.jpg]

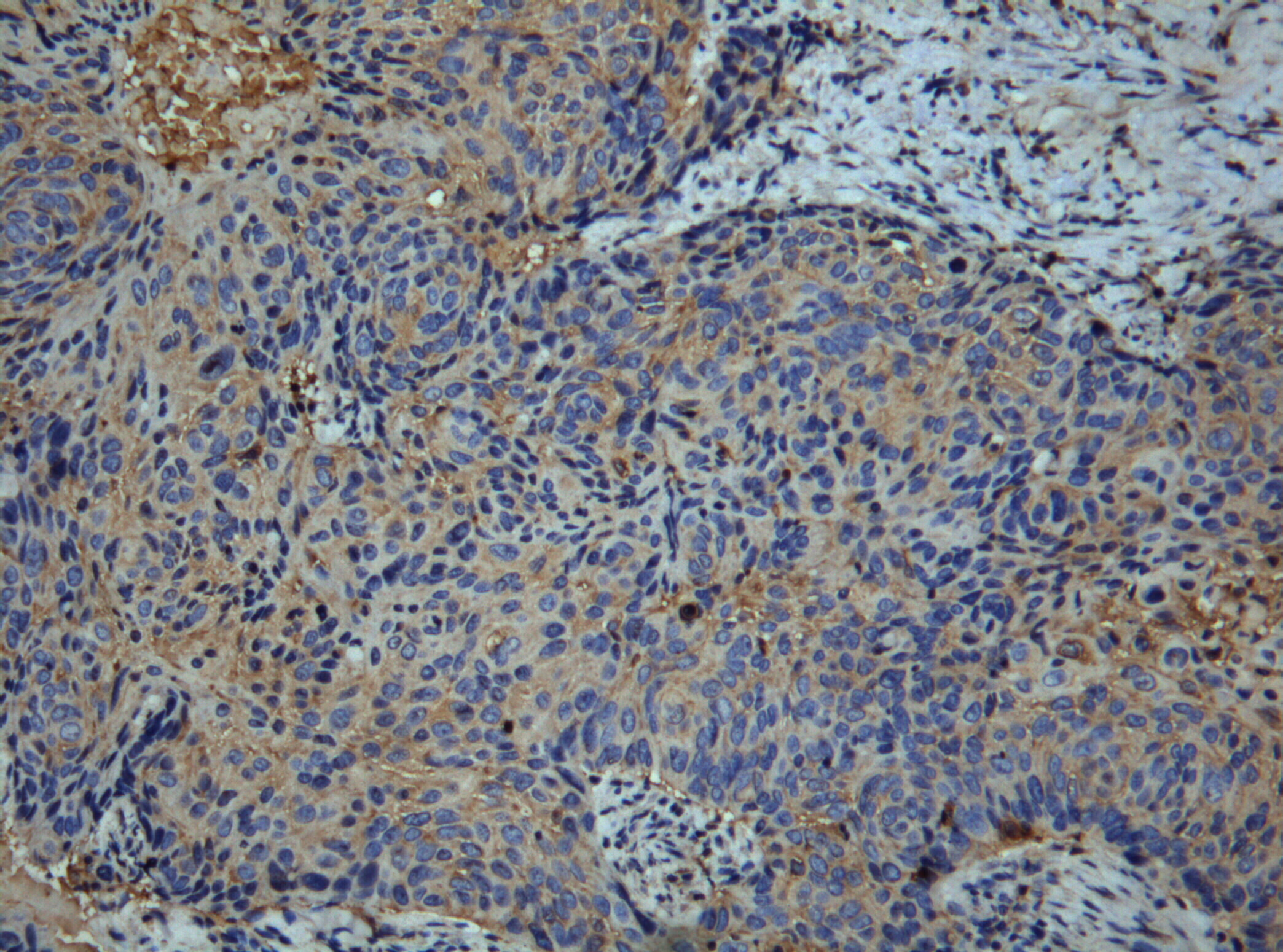

Supplement: S1 File — (ZIP) [file pone.0315242.s001.zip › IHC-TRPC6/23c++.jpg]

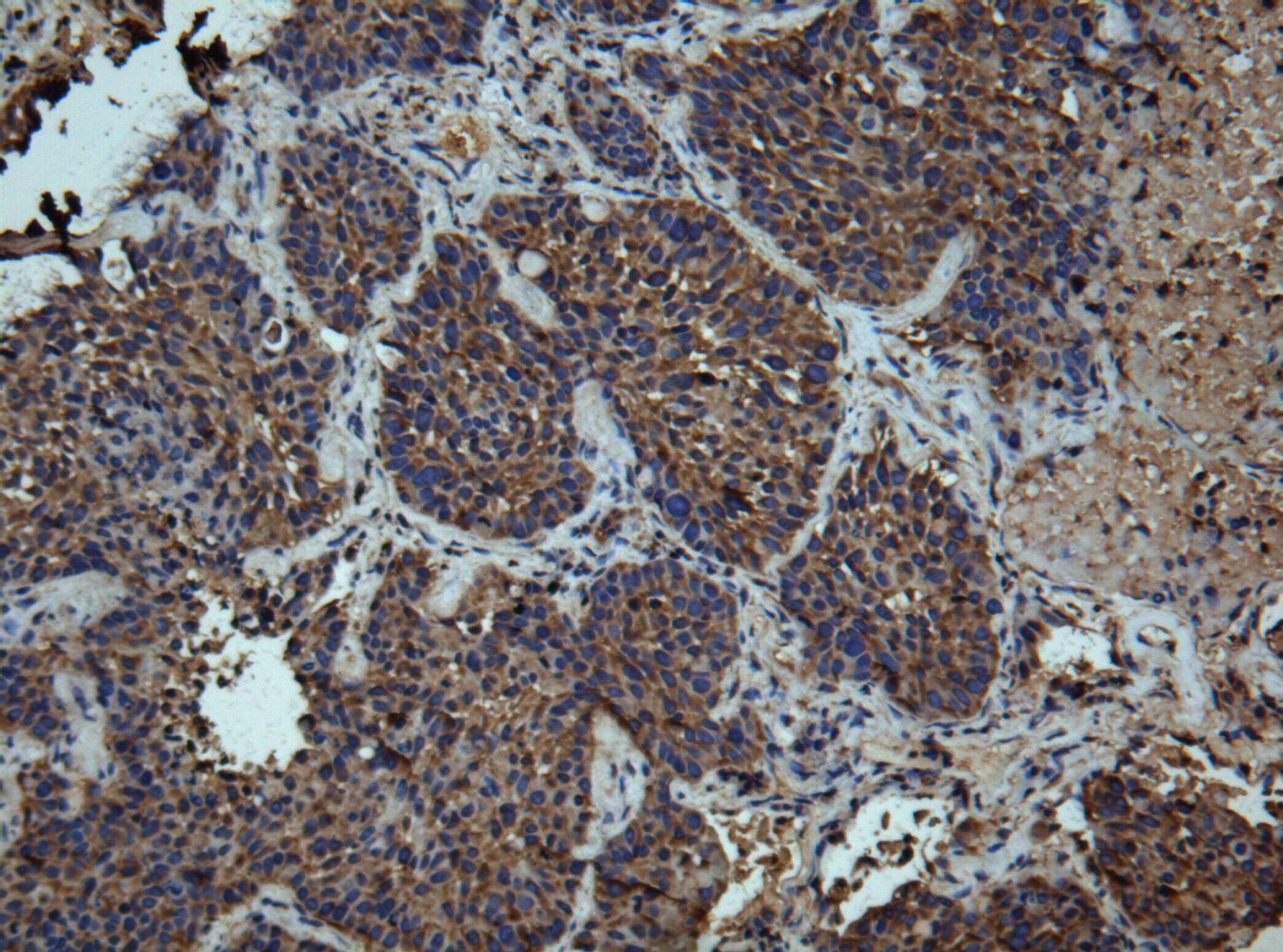

Supplement: S1 File — (ZIP) [file pone.0315242.s001.zip › IHC-TRPC6/24c++.jpg]

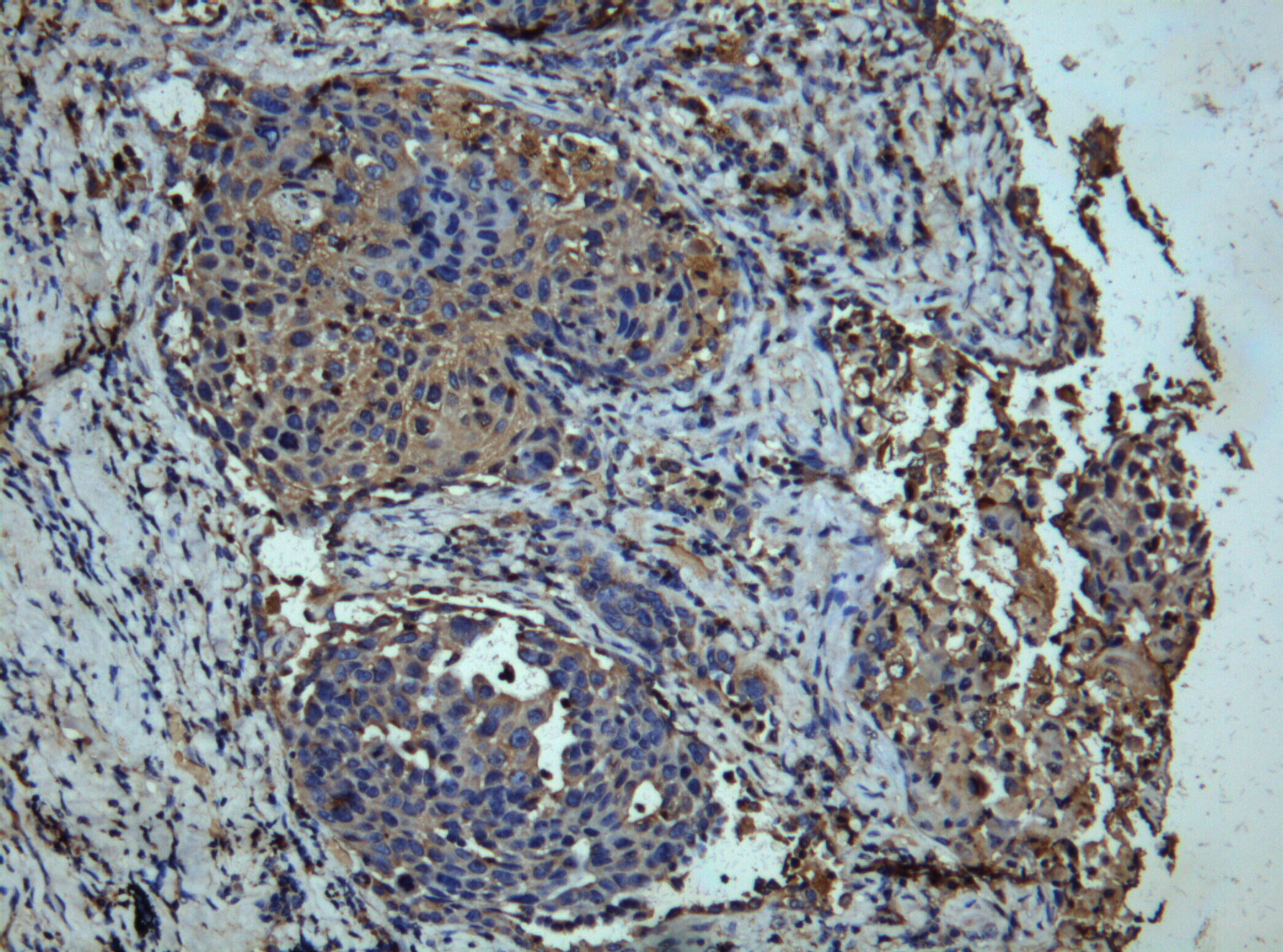

Supplement: S1 File — (ZIP) [file pone.0315242.s001.zip › IHC-TRPC6/25c+.jpg]

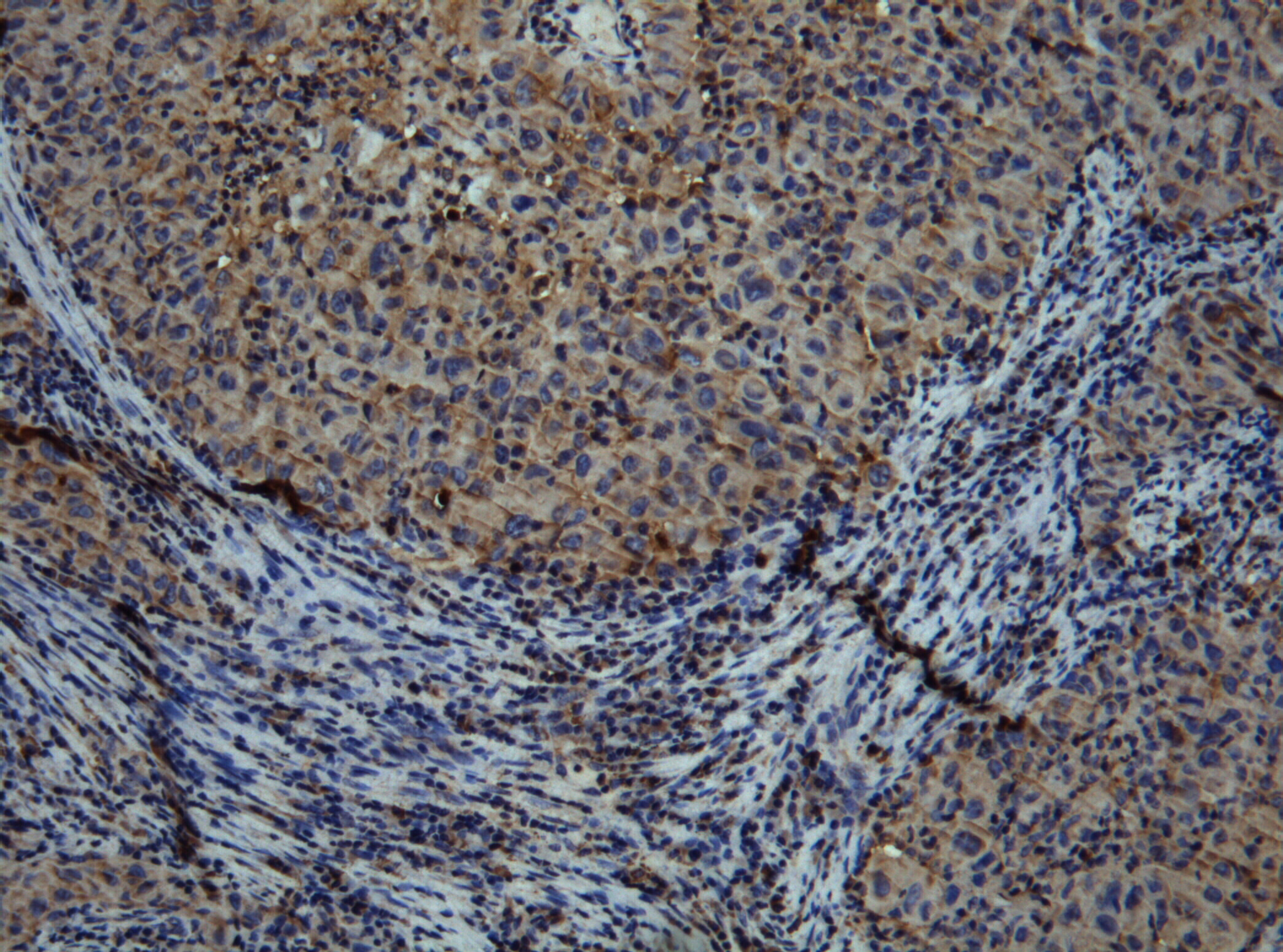

Supplement: S1 File — (ZIP) [file pone.0315242.s001.zip › IHC-TRPC6/27c++.jpg]

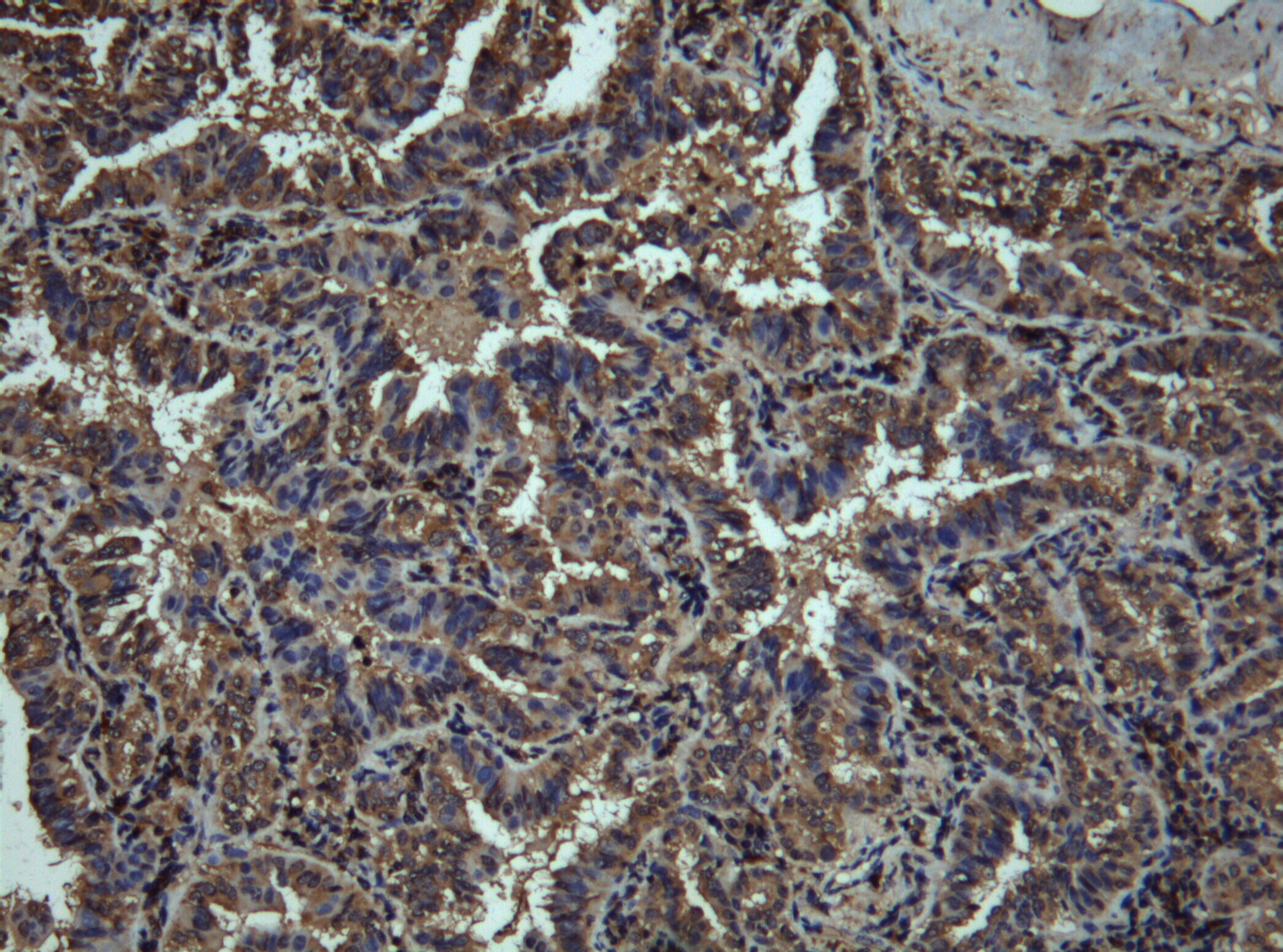

Supplement: S1 File — (ZIP) [file pone.0315242.s001.zip › IHC-TRPC6/28c++~+++.jpg]

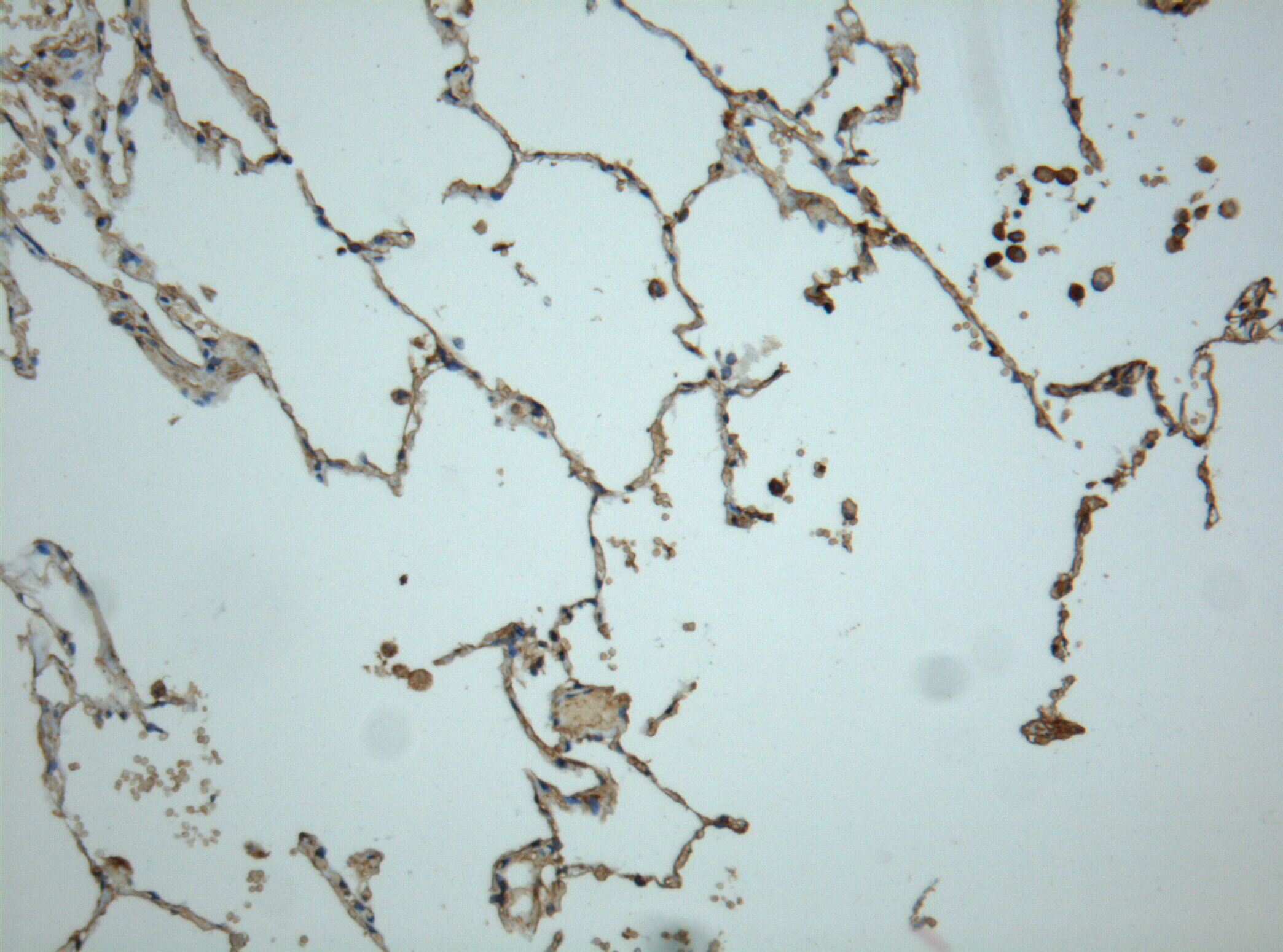

Supplement: S1 File — (ZIP) [file pone.0315242.s001.zip › TRPC1-N.jpg]

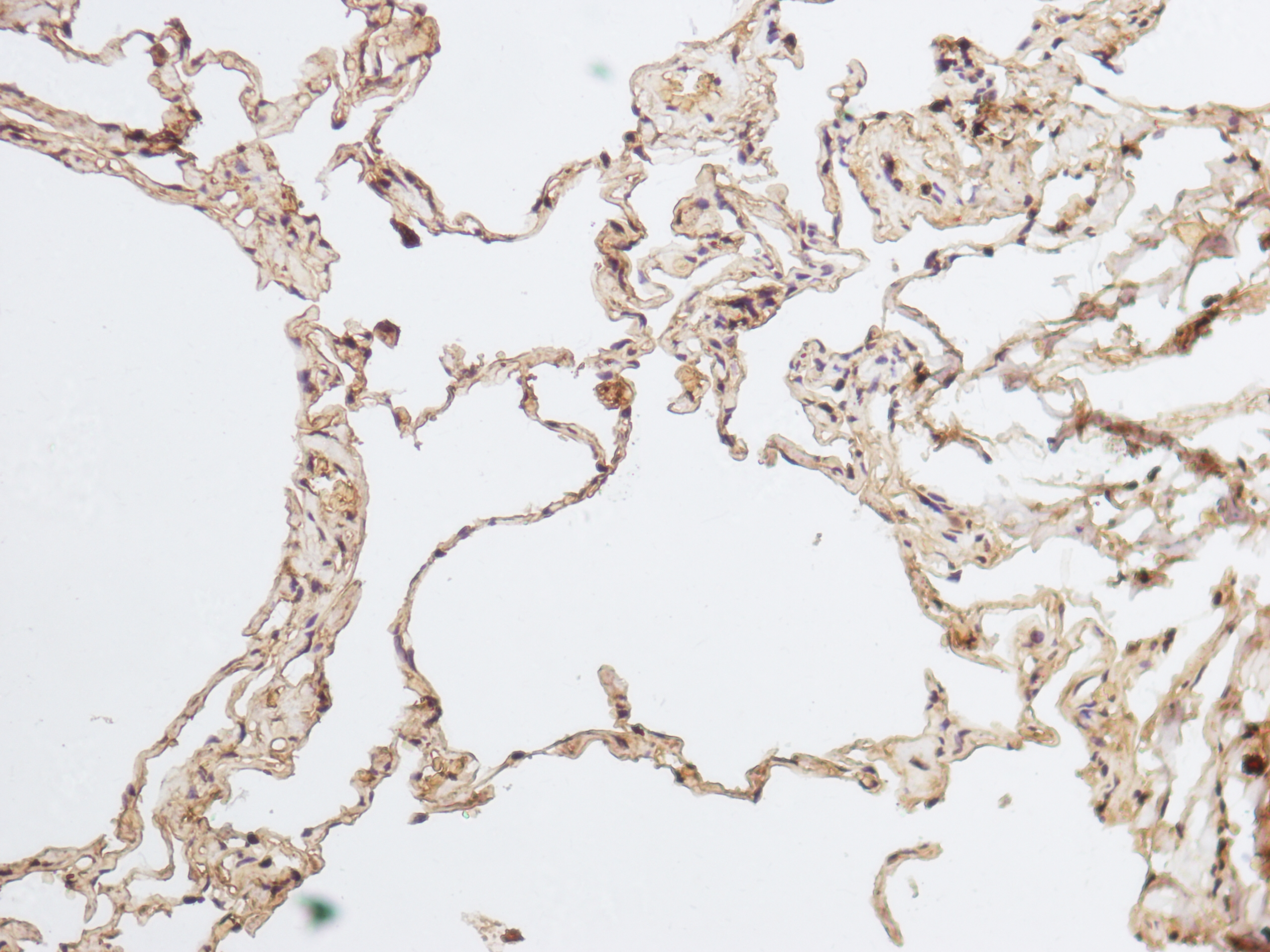

Supplement: S1 File — (ZIP) [file pone.0315242.s001.zip › TRPC3-N.jpg]

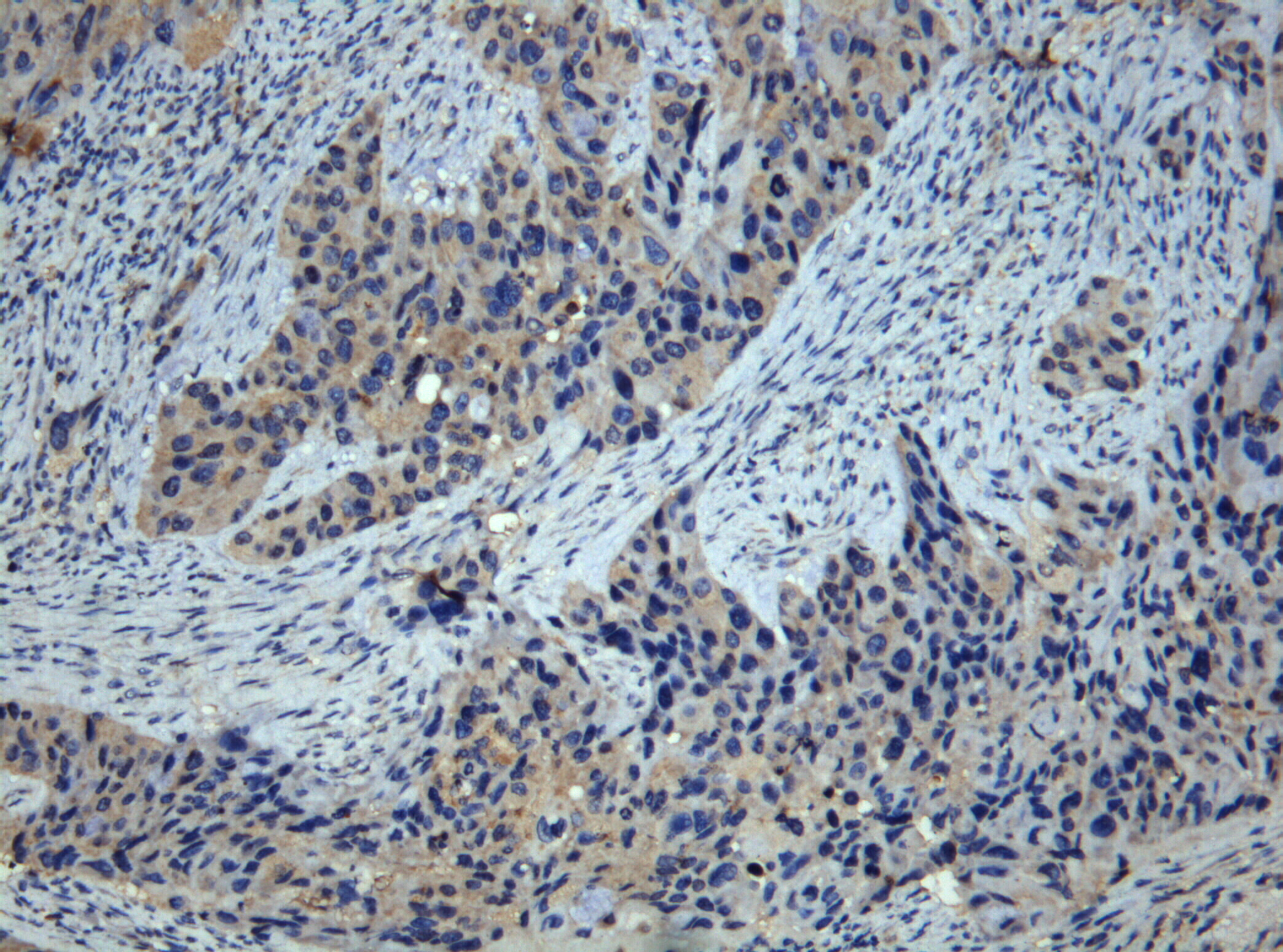

Supplement: S1 File — (ZIP) [file pone.0315242.s001.zip › TRPC4-AC.jpg]

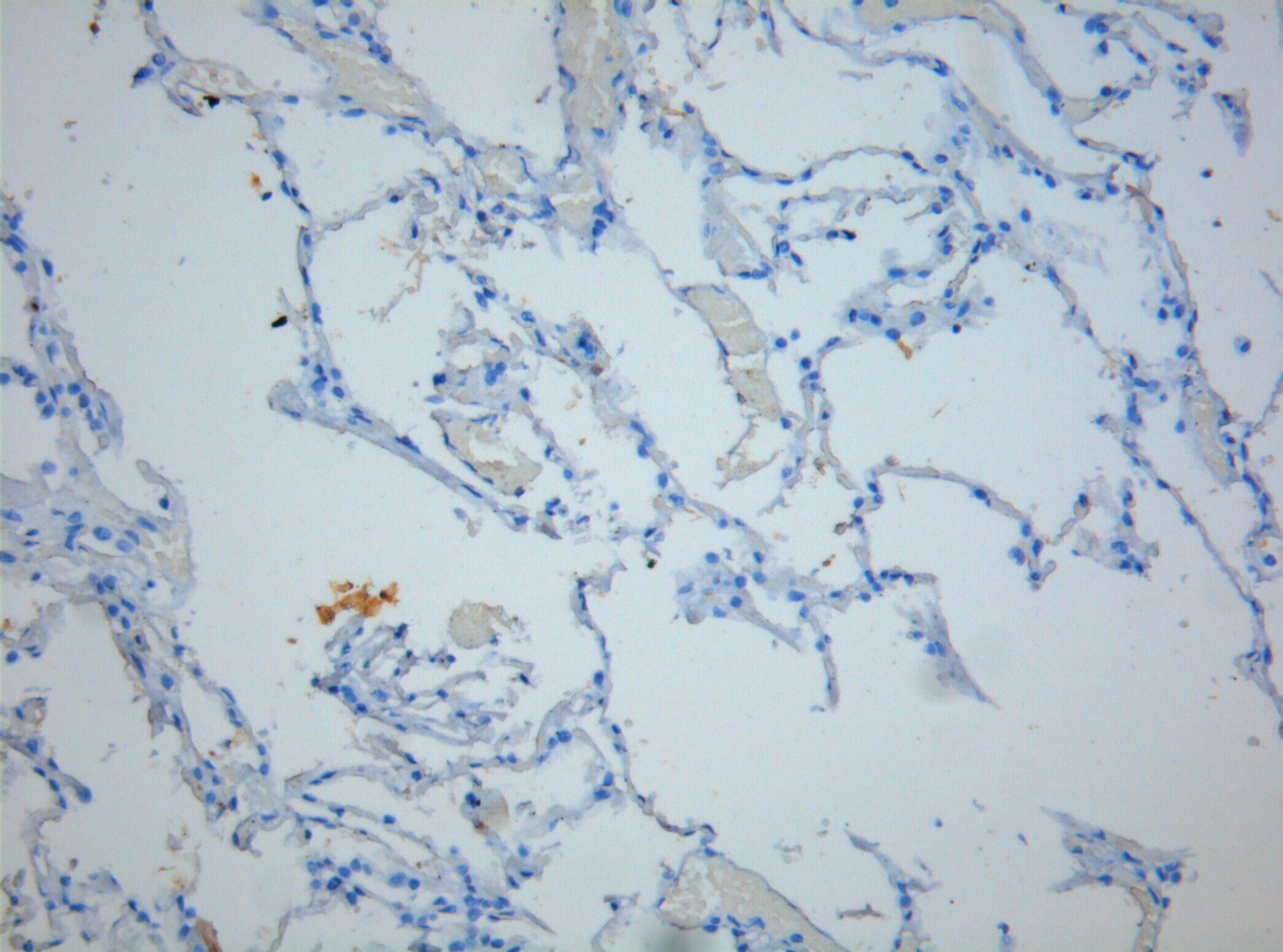

Supplement: S1 File — (ZIP) [file pone.0315242.s001.zip › TRPC4-N.jpg]

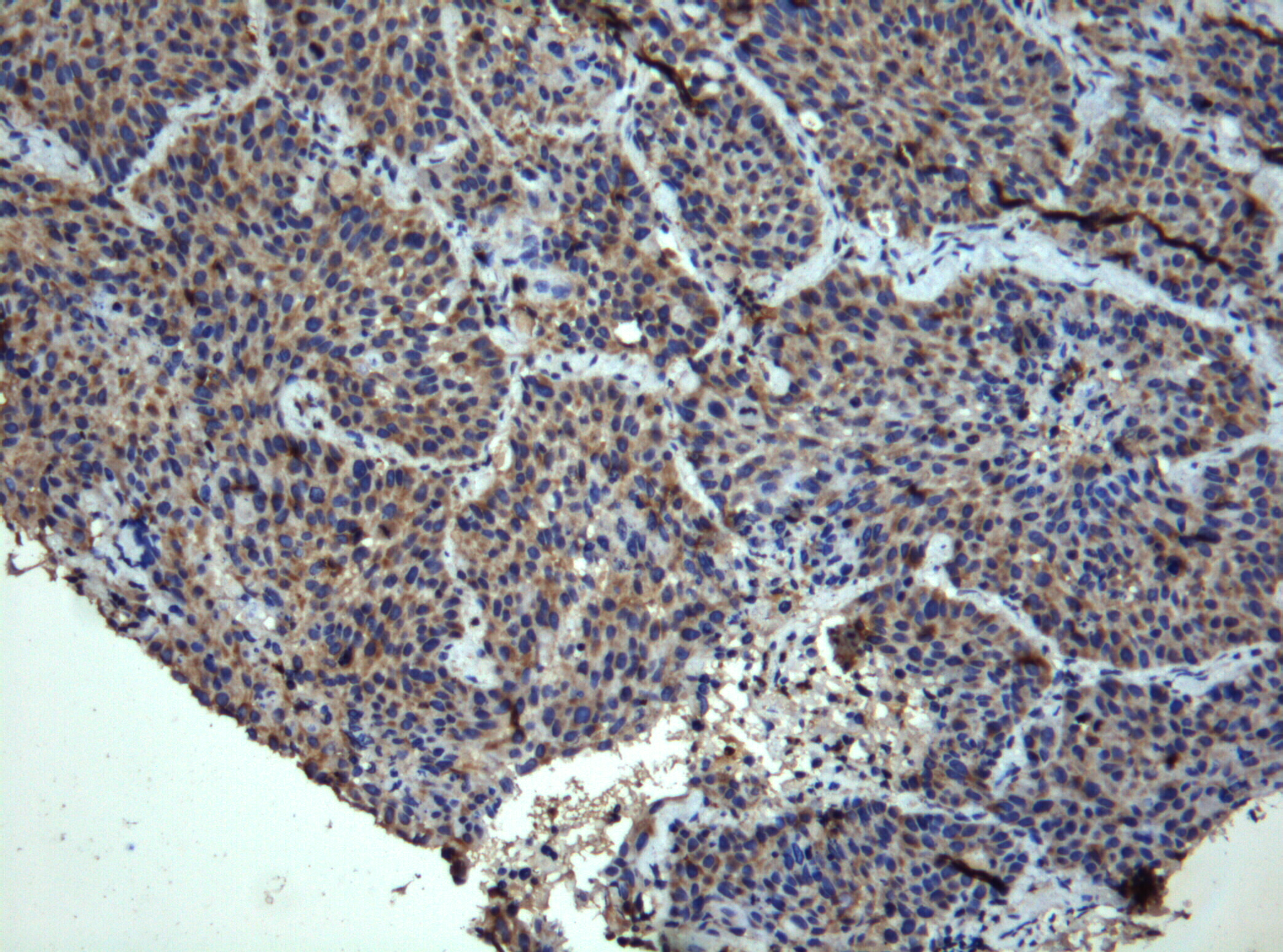

Supplement: S1 File — (ZIP) [file pone.0315242.s001.zip › TRPC4-SCC.jpg]

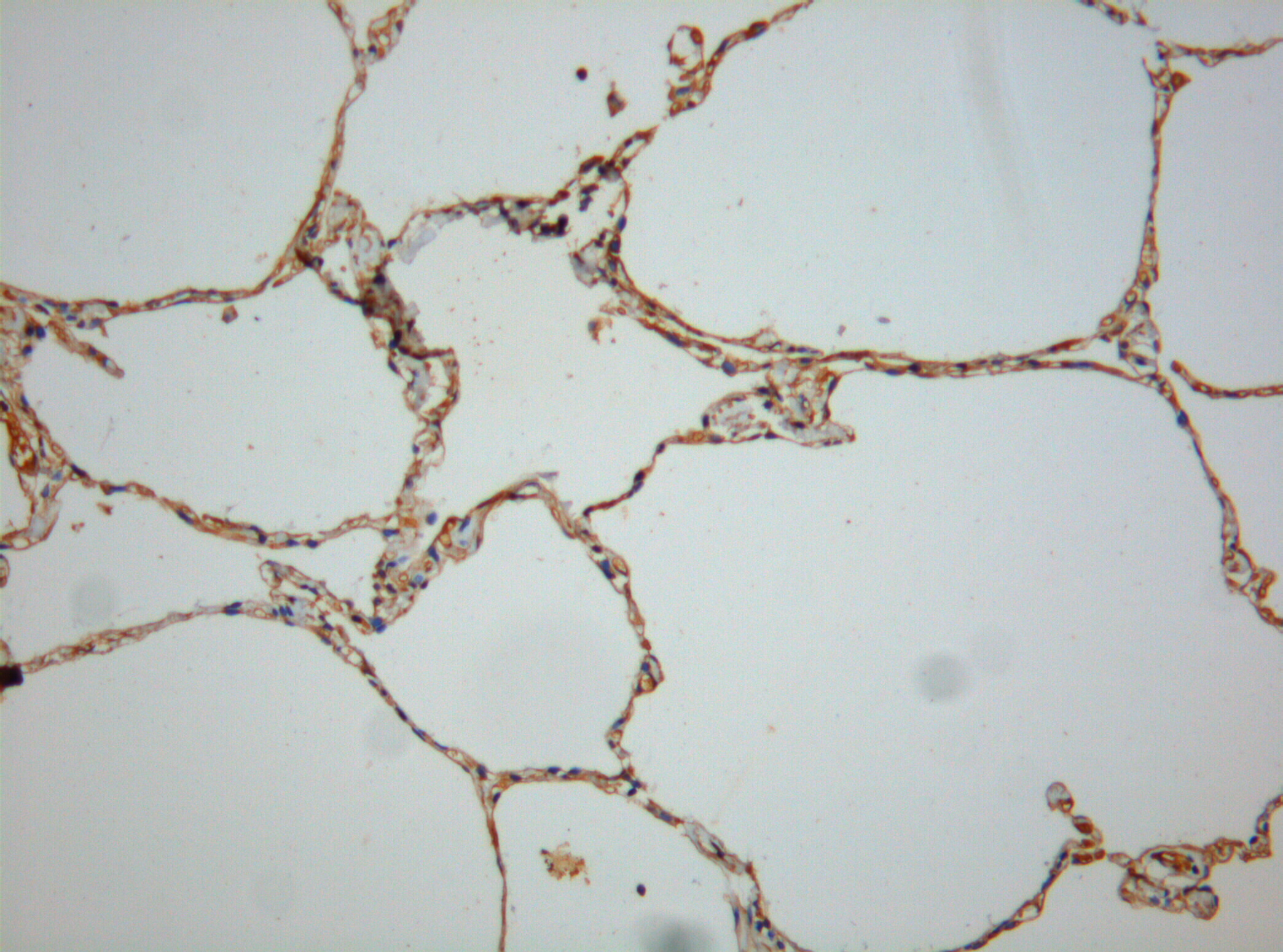

Supplement: S1 File — (ZIP) [file pone.0315242.s001.zip › TRPC6-N.jpg]

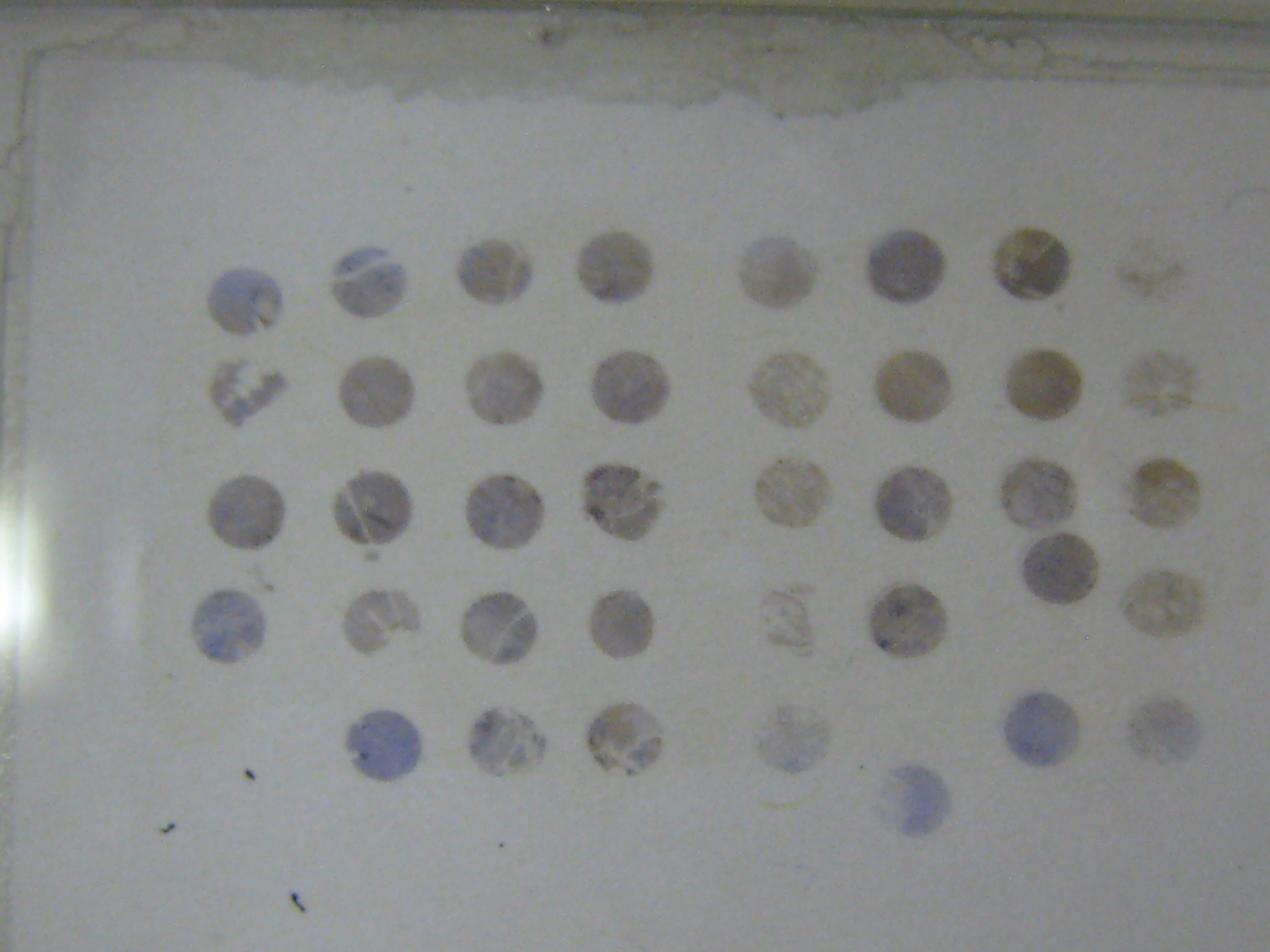

Supplement: S3 File — (ZIP) [file pone.0315242.s003.zip › TRPC1-chip1.JPG]

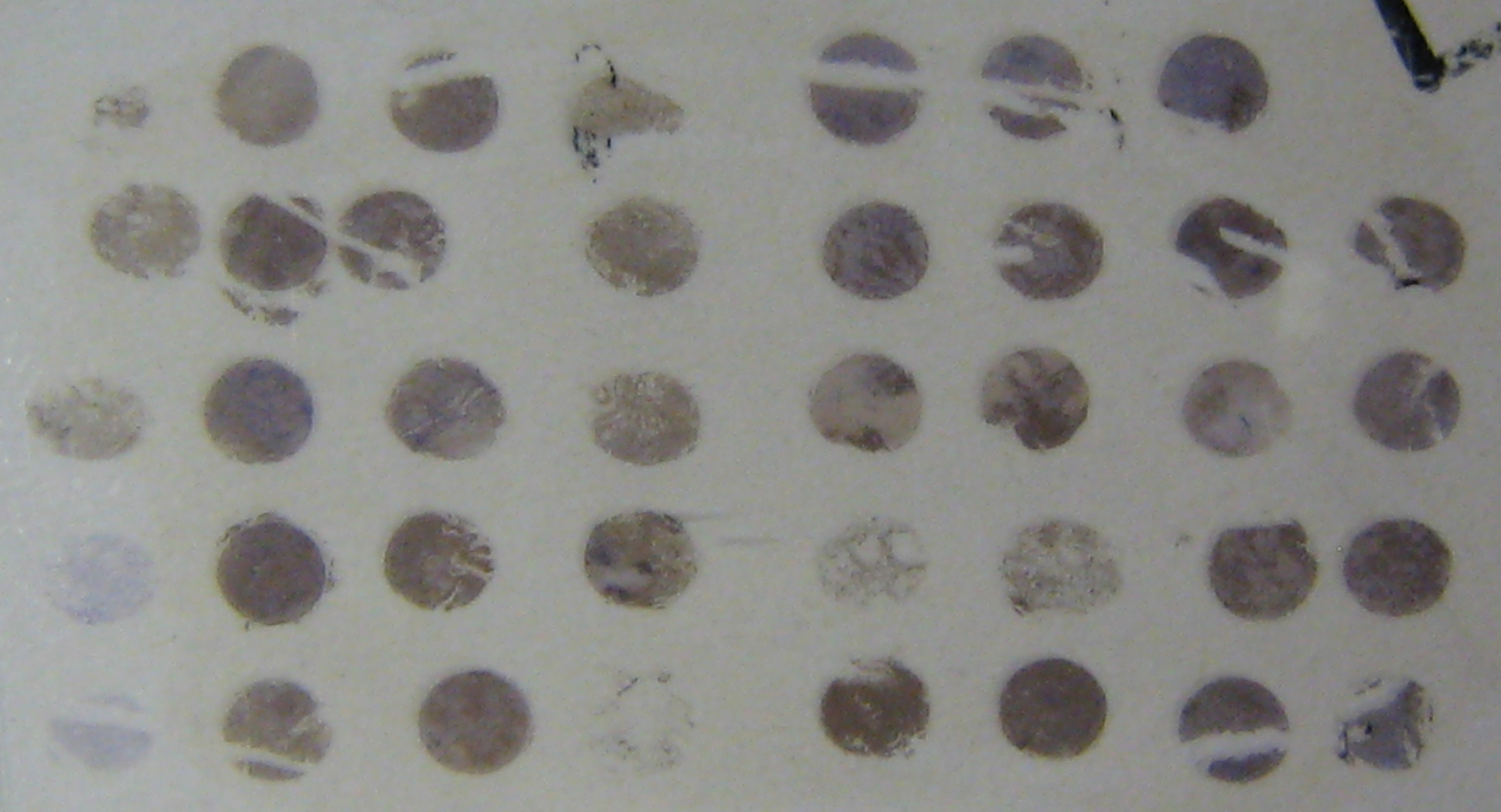

Supplement: S3 File — (ZIP) [file pone.0315242.s003.zip › TRPC1-chip2.jpg]

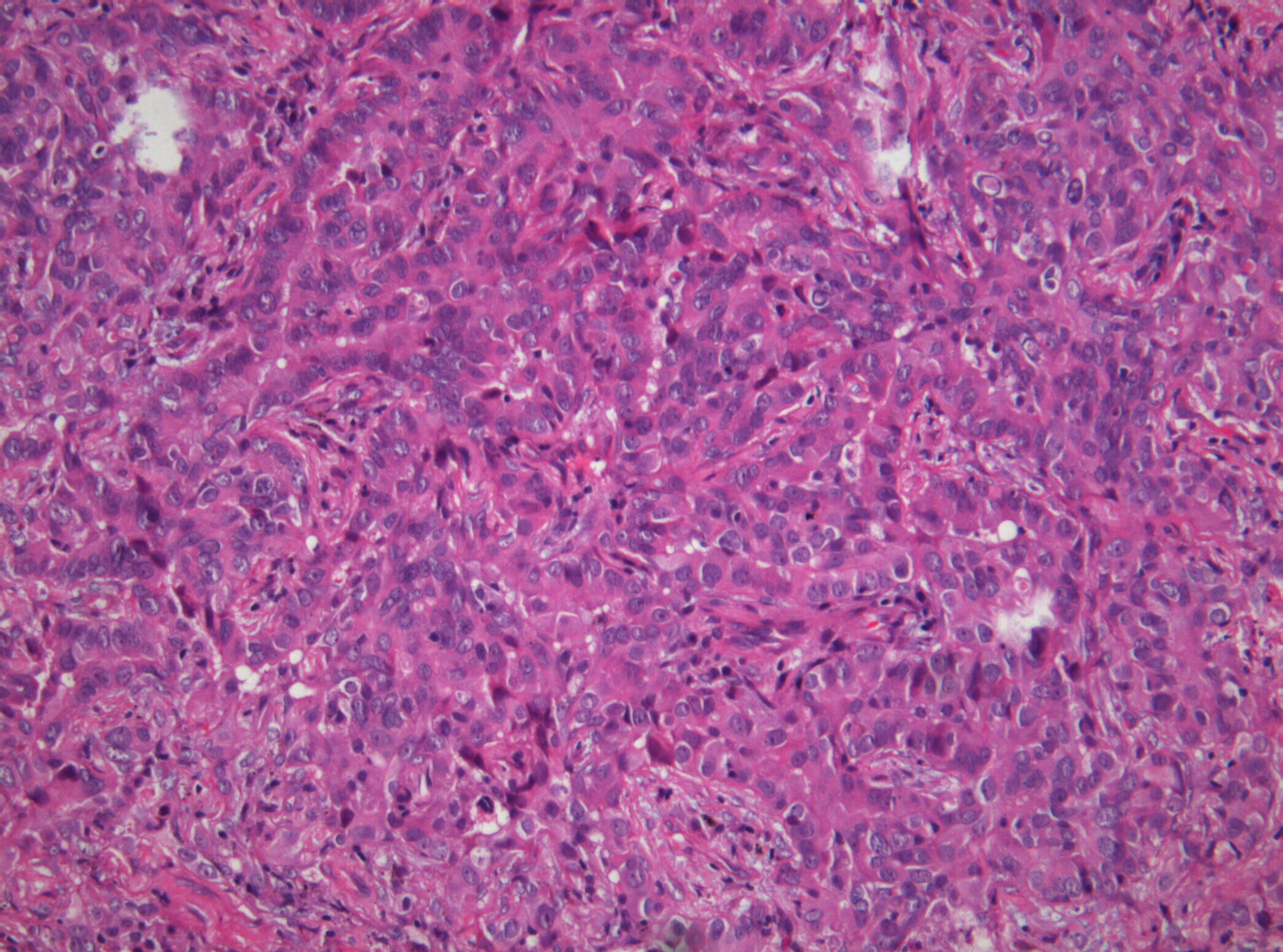

Supplement: S3 File — (ZIP) [file pone.0315242.s003.zip › HE-9C-AC.jpg]

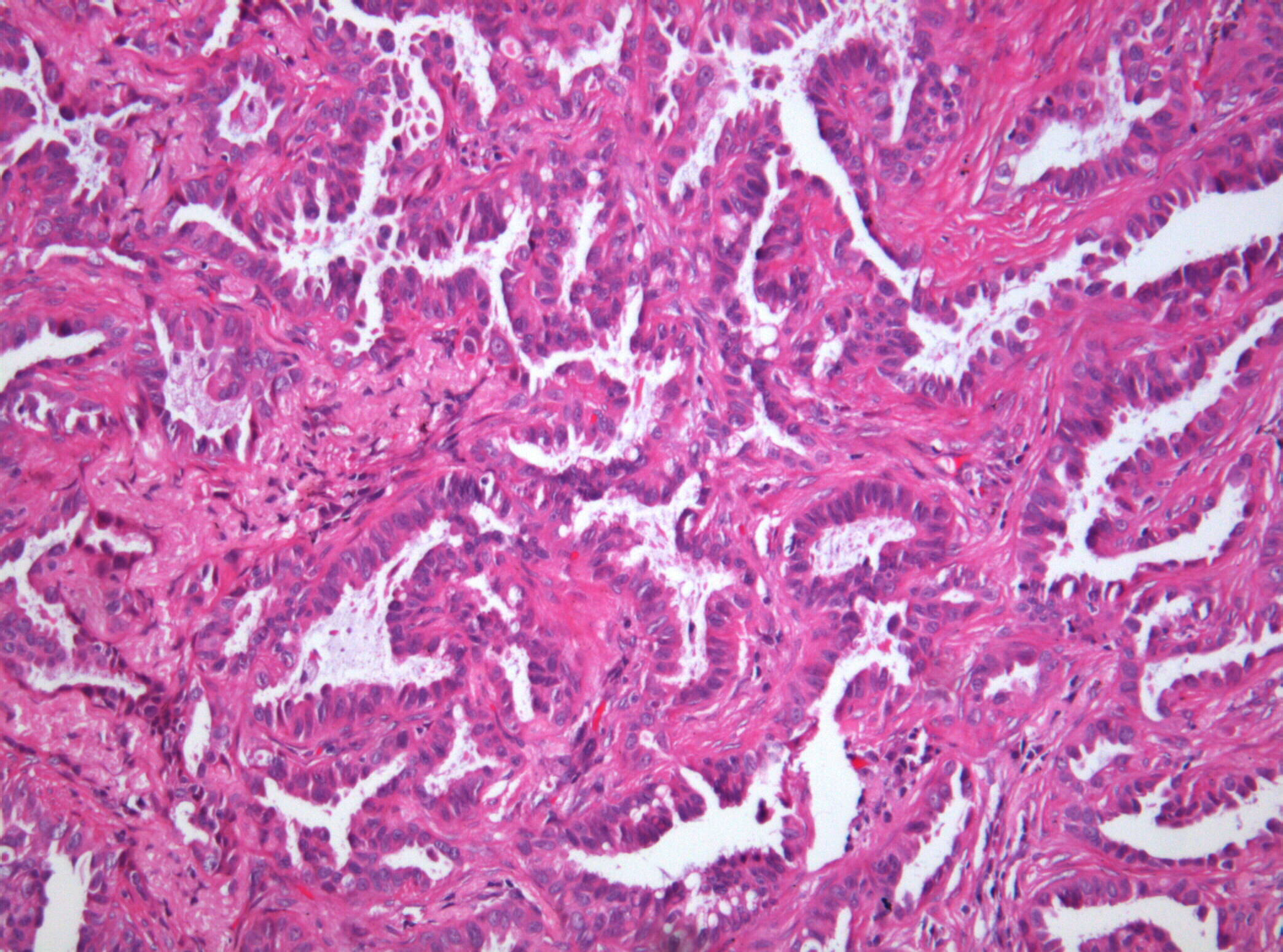

Supplement: S3 File — (ZIP) [file pone.0315242.s003.zip › HE-11C-AC.jpg]

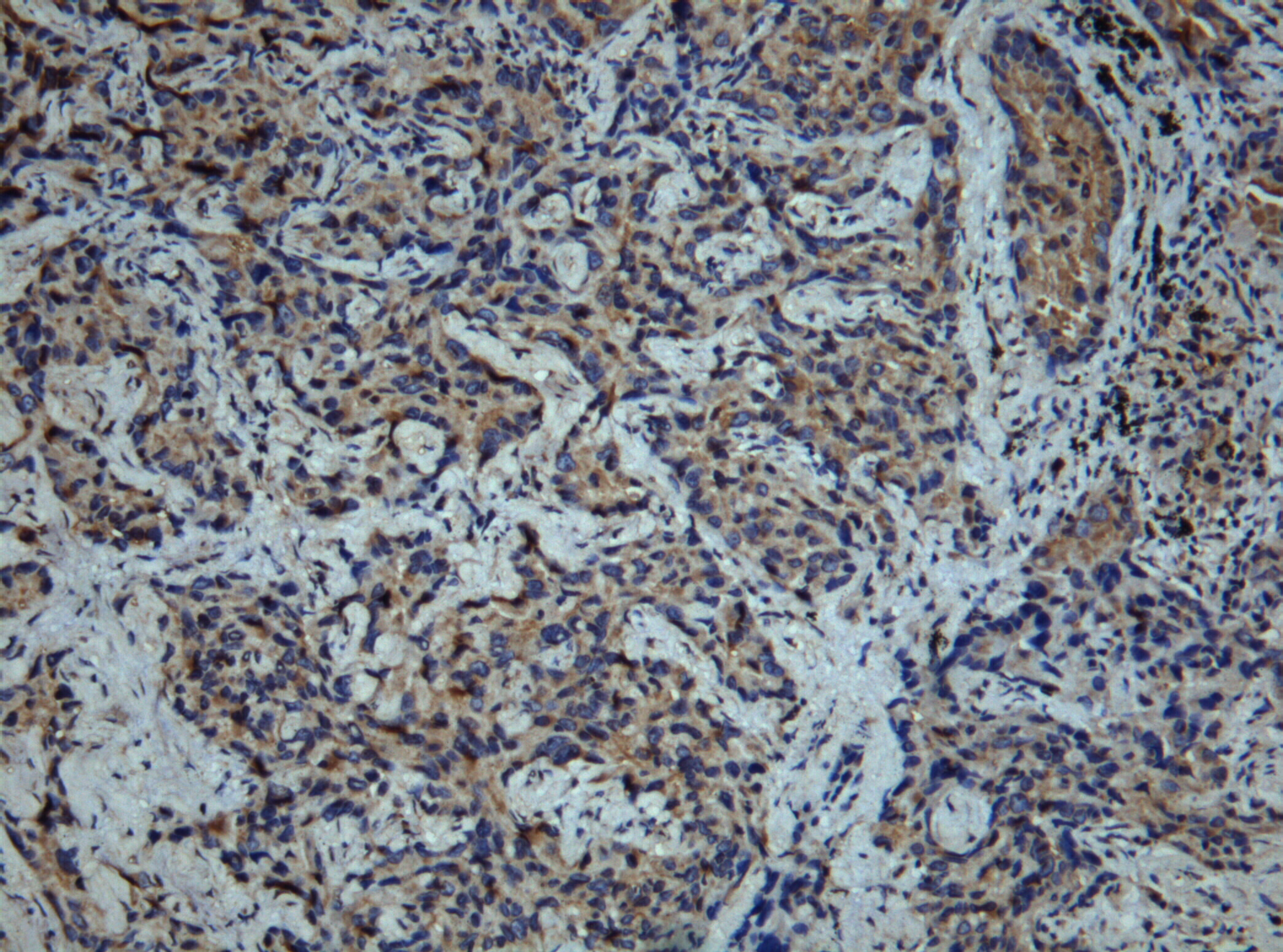

Supplement: S3 File — (ZIP) [file pone.0315242.s003.zip › TRPC1-9C-AC.jpg]

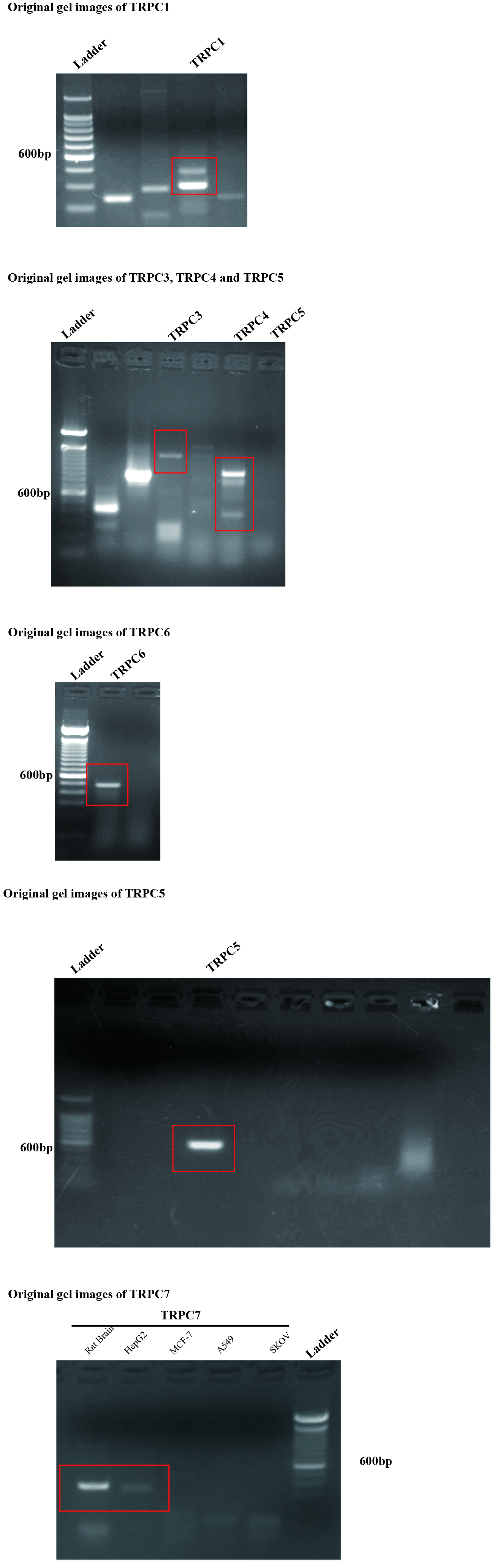

Supplement: S5 File — (JPG) [file pone.0315242.s005.jpg]
