# Supplementary material for: Correction: Involvement of TRPC Channels in Lung Cancer Cell Differentiation and the Correlation Analysis in Human Non-Small Cell Lung Cancer
Source: PLoS One. 2024 Dec 5;19(12):e0315242. doi: 10.1371/journal.pone.0315242 (PMC11620368; doi:10.1371/journal.pone.0315242)
Supplement: S6 File — (PPTX) [file pone.0315242.s006.pptx]

## Slide 1
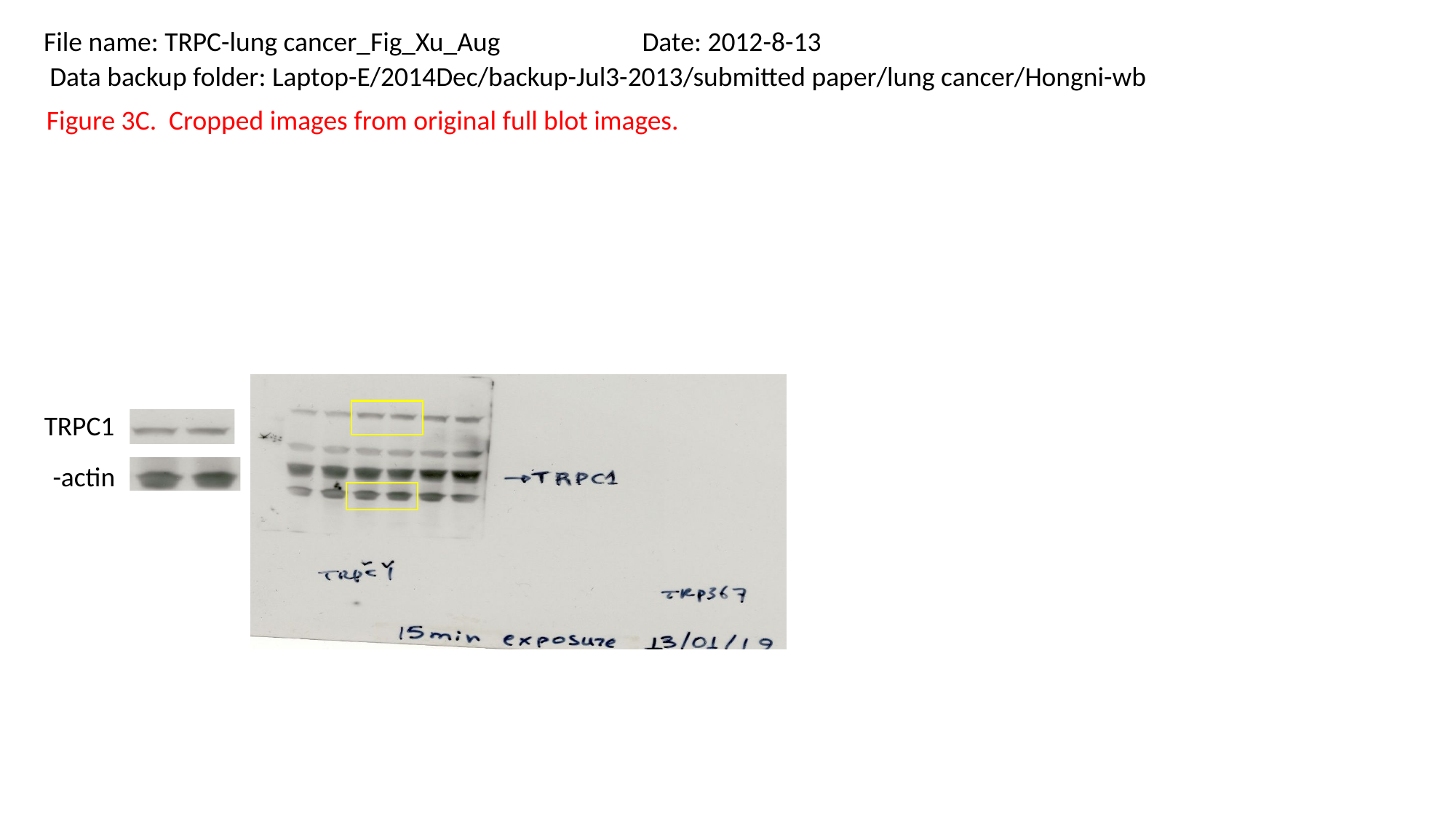

File name: TRPC-lung cancer_Fig_Xu_Aug Date: 2012-8-13
Data backup folder: Laptop-E/2014Dec/backup-Jul3-2013/submitted paper/lung cancer/Hongni-wb
Figure 3C. Cropped images from original full blot images.
TRPC1

## Slide 2
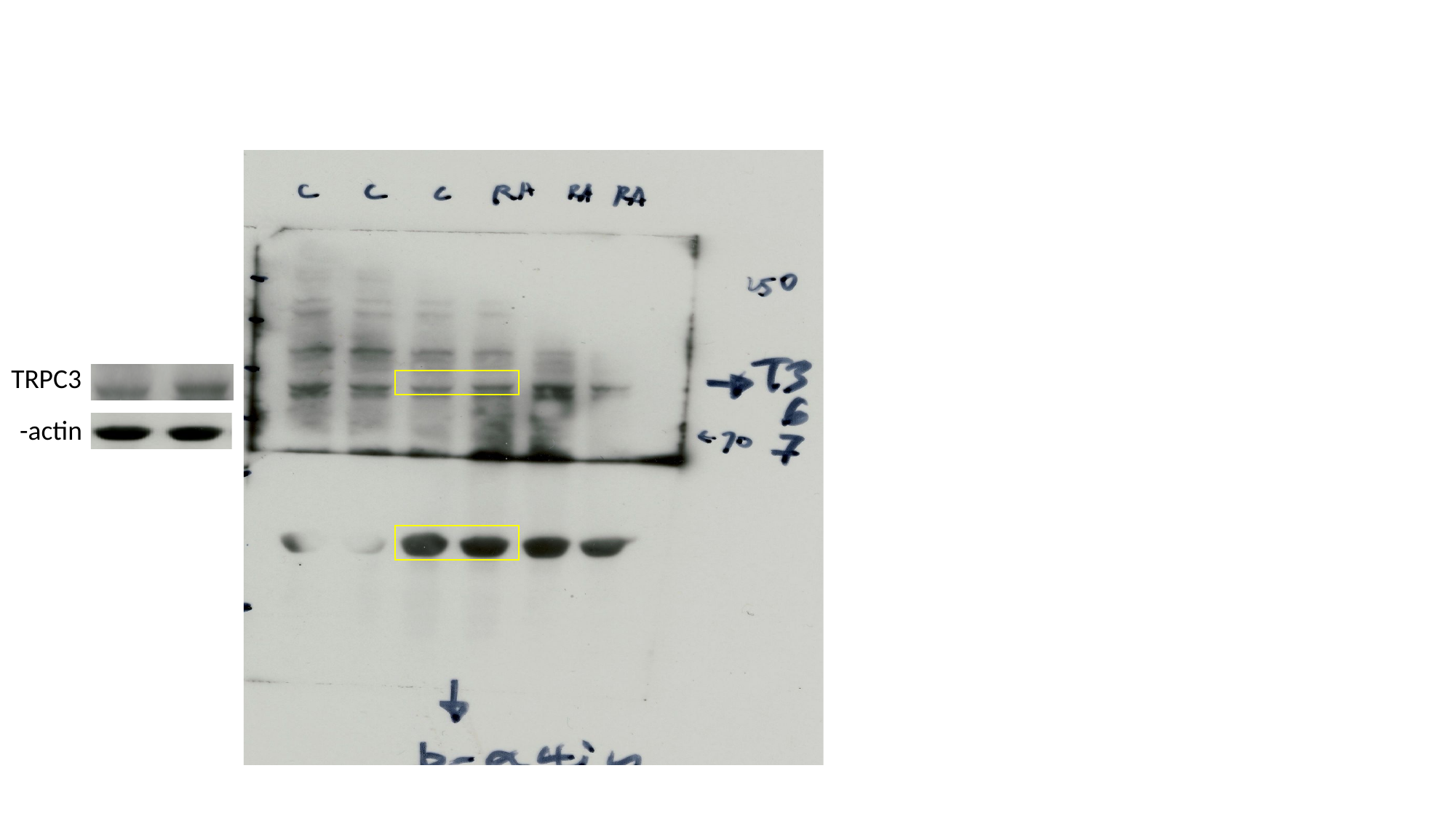

TRPC3

## Slide 3
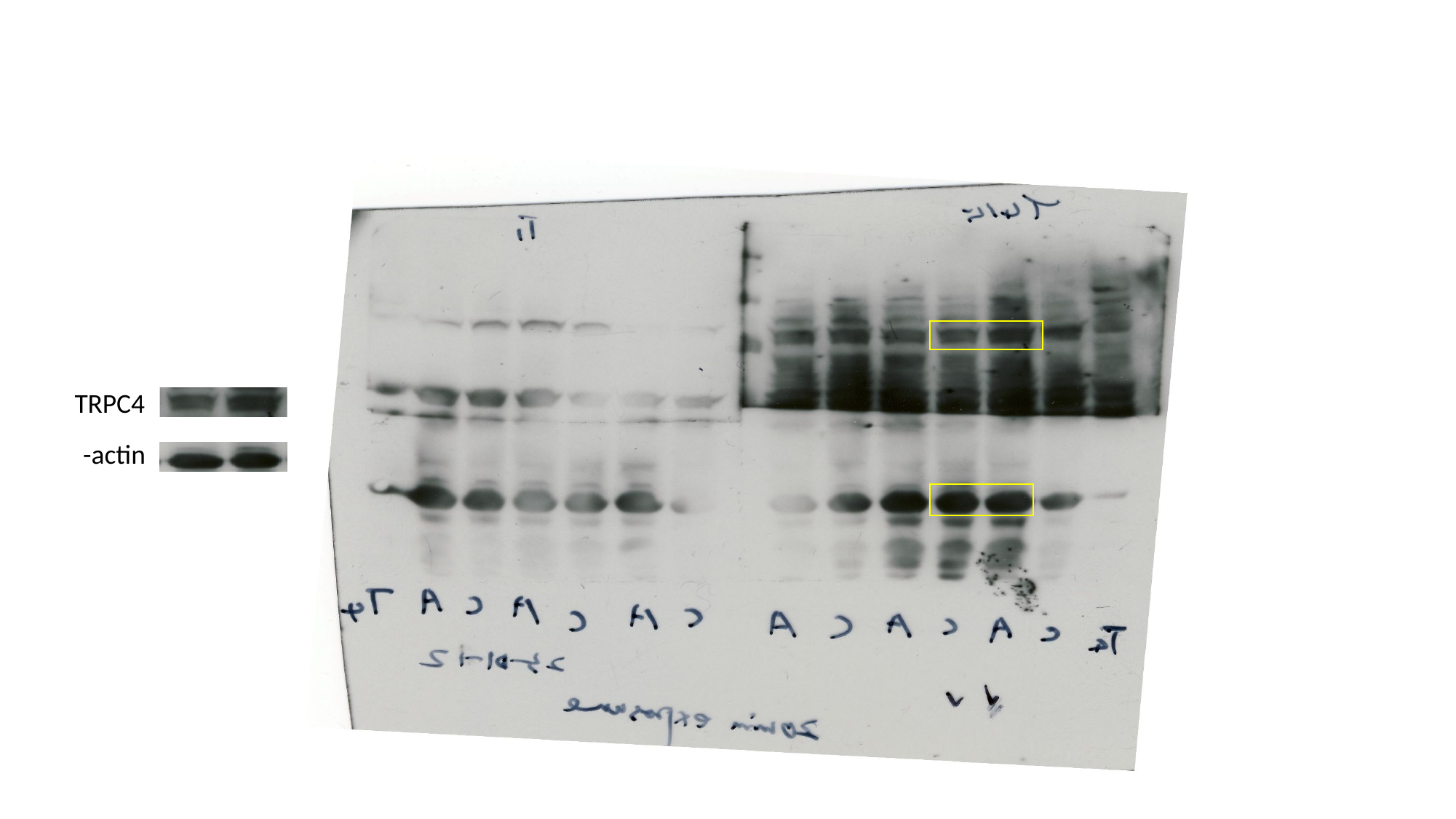

TRPC4

## Slide 4
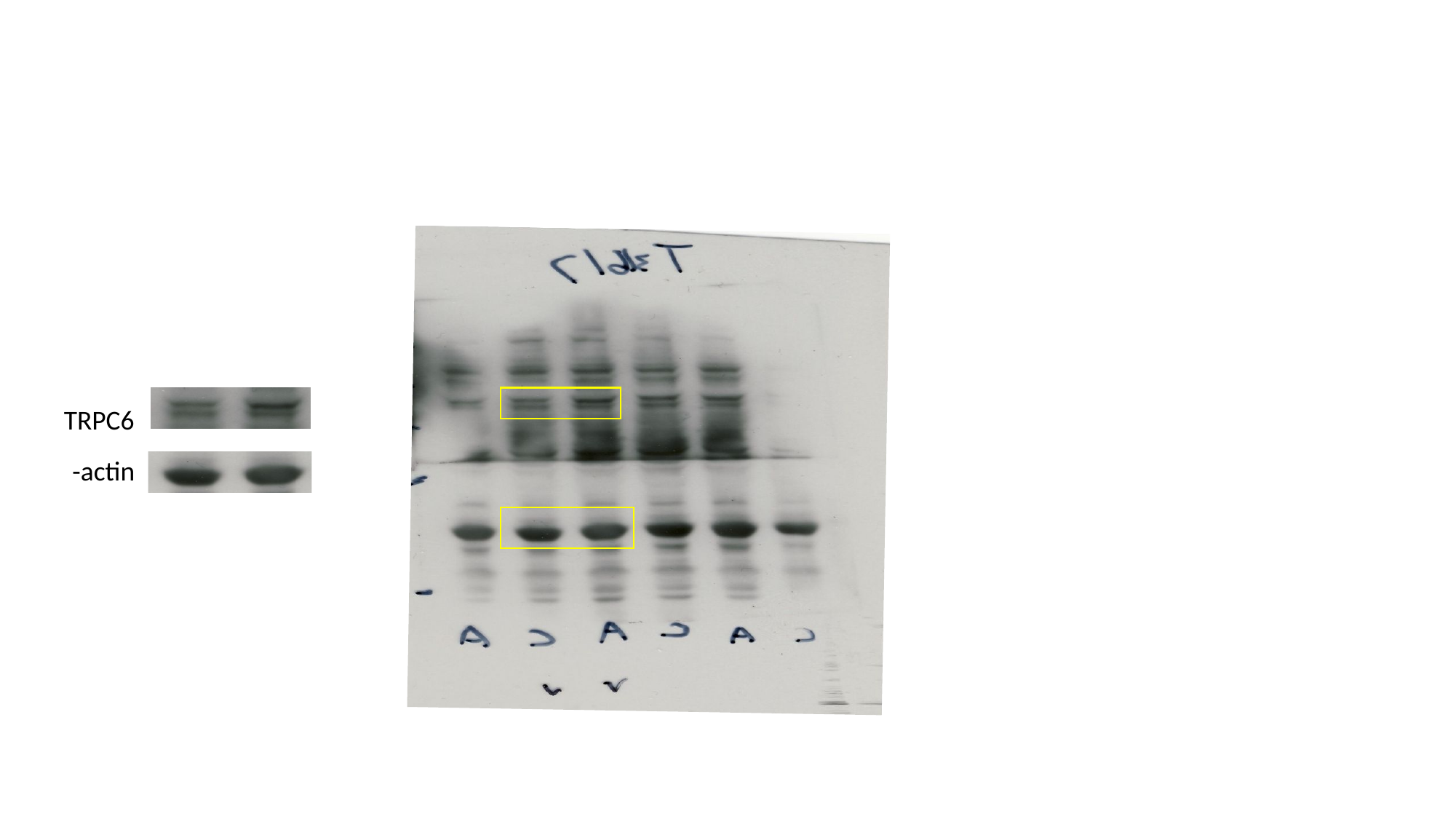

TRPC6
